# Genome-wide meta-analysis identifies nine loci associated with higher risk of hepatocellular carcinoma development

**DOI:** 10.1016/j.jhepr.2025.101485

**Published:** 2025-06-11

**Authors:** Jonas Ghouse, Helene Gellert-Kristensen, Colm J. O’Rourke, Anne-Sofie Seidelin, Gudmar Thorleifsson, Gardar Sveinbjörnsson, Vinicius Tragante, Chigoziri Konkwo, Joseph Brancale, Silvia Vilarinho, Tim M. Eyrich, Gustav Ahlberg, Johan S. Bundgaard, Søren A. Rand, Pia R. Lundegaard, Erik Sørensen, Christina Mikkelsen, Jacob Træholt, Christian Erikstrup, Khoa M. Dinh, Mie T. Bruun, Bitten Aa. Jensen, Jakob T. Bay, Søren Brunak, Karina Banasik, Henrik Ullum, DBDS Genomic Consortium, Estonian Biobank Research Team, Triin Laisk, Reedik Mägi, Lincoln D. Nadauld, Kirk U. Knowlton, Stacey Knight, Lise L. Gluud, Kirsten Vistisen, Einar S. Björnsson, Magnus O. Ulfarsson, Patrick Sulem, Hilma Holm, Ole B. Pedersen, Sisse R. Ostrowski, Daniel F. Gudbjartsson, Thorunn Rafnar, Kari Stefansson, Ulrik Lassen, Hans-Christian Pommergaard, Jens G. Hillingsø, Jesper B. Andersen, Henning Bundgaard, Stefan Stender

**Affiliations:** 1 Department of Cardiology, Rigshospitalet, Copenhagen University Hospital, Copenhagen, Denmark; 2 Cardiac Genetics Group, Department of Biomedical Sciences, University of Copenhagen, Copenhagen, Denmark; 3 Department of Clinical Biochemistry, Rigshospitalet, Copenhagen University Hospital, Copenhagen, Denmark; 4 Biotech Research & Innovation Centre (BRIC), Department of Health and Medical Sciences, University of Copenhagen, Copenhagen, Denmark; 5 deCODE genetics/Amgen, Inc, Reykjavik, Iceland; 6 Section of Digestive Diseases, Department of Internal Medicine, Yale School of Medicine, New Haven, CT, USA; 7 Department of Genetics, Yale School of Medicine, New Haven, CT, USA; 8 Department of Pathology, Yale School of Medicine, New Haven, CT, USA; 9 Department of Clinical Immunology, Rigshospitalet, Copenhagen University Hospital, Copenhagen, Denmark; 10 Novo Nordisk Foundation Center for Basic Metabolic Research, Faculty of Health and Medical Science, Copenhagen University, Copenhagen, Denmark; 11 Department of Clinical Immunology, Aarhus University Hospital, Aarhus, Denmark; 12 Department of Clinical Immunology, Odense University Hospital, Odense, Denmark; 13 Department of Clinical Immunology, Aalborg University Hospital, Aalborg, Denmark; 14 Department of Clinical Immunology, Zealand University Hospital, Køge, Denmark; 15 Translational Disease Systems Biology, Novo Nordisk Foundation Center for Protein Research, Faculty of Health and Medical Sciences, University of Copenhagen, Copenhagen, Denmark; 16 Department of Obstetrics and Gynaecology, Copenhagen University Hospital Hvidovre, Hvidovre, Denmark; 17 Statens Serum Institut, Copenhagen, Denmark; 18 Estonian Genome Centre, Institute of Genomics, University of Tartu, Tartu, Estonia; 19 Intermountain Health, Salt Lake City, UT 84111, USA; 20 Gastro Unit, Copenhagen University Hospital Hvidovre, Hvidovre, Denmark; 21 Department of Clinical Medicine, University of Copenhagen, Copenhagen, Denmark; 22 Department of Oncology, Copenhagen University Hospital-Herlev and Gentofte, Herlev, Denmark; 23 Faculty of Medicine, University of Iceland, Reykjavik, Iceland; 24 Internal Medicine and Emergency Services, Landspitali – The National University Hospital of Iceland, Reykjavik, Iceland; 25 Faculty of Electrical and Computer Engineering, University of Iceland, Reykjavik, Iceland; 26 School of Engineering and Natural Sciences, University of Iceland, Reykjavik, Iceland; 27 Department of Oncology, Rigshospitalet, Copenhagen University Hospital, Copenhagen, Denmark; 28 Hepatic Malignancy Surgical Research Unit (HEPSURU), Department of Surgery and Transplantation, Rigshospitalet, Copenhagen University Hospital, Copenhagen, Denmark

**Keywords:** HCC, Cirrhosis, MASLD, Biliary tract cancer, PNPLA3

## Abstract

**Background & Aims:**

The genetic underpinnings of hepatocellular carcinoma (HCC) remain largely unknown. Thus, we aimed to identify new genetic risk loci for HCC.

**Methods:**

We performed a genome-wide association study (GWAS) meta-analysis of 11 cohorts with validation in two independent cohorts. The identified variants were tested for effects on other hepatobiliary endpoints, and on incident HCC stratified by underlying risk factors. Mendelian randomization was used to assess the causal effects of a range of traits on the risk of HCC.

**Results:**

In meta-analyses totaling 6,540 cases and 2,096,759 controls, we identified 10 associations with HCC, of which five (in *KLF15*, *HSD17B13*, *APOE*, *HFE*, and *MTARC1*) have not previously been implicated in HCC at genome-wide statistical significance. Known associations in *PNPLA3*, *TM6SF2*, *TERT*, *IFNL4*, and *HLA-DP1* were confirmed. All associations except *KLF15* were validated in independent cohorts totaling 7,630 cases and 733,689 controls. The largest per-allele effect was seen for *TM6SF2* (beta = 0.61) followed by *PNPLA3* (0.55), *HFE* (0.45), *IFNL4* (0.31), *APOE* (0.27), *HSD17B13*, *HLA-DP1*, and *TERT* (all 0.21), and *MTARC1* (0.17). The identified variants had comparable effects on incident HCC in individuals with prevalent obesity, a high alcohol intake, diabetes, or cirrhosis. Mendelian randomization analyses confirmed the causal role of obesity in HCC. We found strong correlations between genetic effects on HCC and hepatic steatosis (r^2^ = 0.75), and HCC and cirrhosis (r^2^ = 0.69), whereas only three loci (*APOE*, *HFE*, and *TERT*) had concordant effects on HCC and biliary tract cancer.

**Conclusions:**

We identified and validated nine genetic variants associated with an increased risk of HCC development.

**Impact and implications:**

The genetic underpinnings of HCC remain largely unknown. In this genome-wide association meta-analysis totaling 6,540 cases with HCC and 2.1 million controls, we identified and validated nine genetic loci to associate with the risk of HCC. A deeper insight into genetic factors that affect the risk of HCC could improve our ability to predict and ultimately prevent or treat this deadly cancer.

## Highlights

• The genetic underpinnings of HCC remain largely unknown.
• We conducted a GWAS meta-analysis of 6,540 HCC cases and 2,096,759 controls, with replication in two independent cohorts.
• We identified and validated nine loci associated with HCC, including five previously unreported loci.
• Genetic effects on HCC and steatosis or cirrhosis were strongly correlated.

## Graphical abstract

**Figure:**
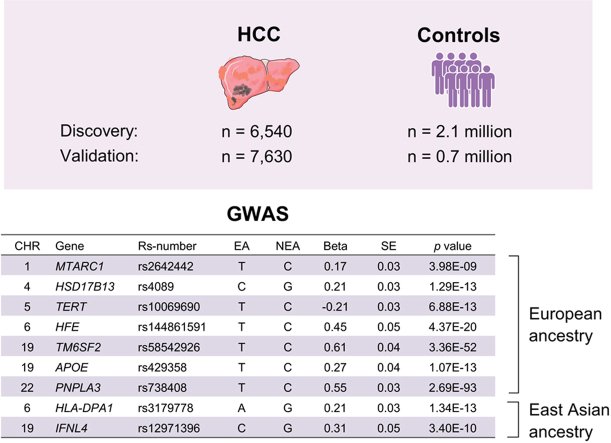

## Introduction

Hepatocellular carcinoma (HCC) is the third most common cause of cancer-related mortality in the world.^1^^,^^2^ The development of HCC occurs most often in cirrhotic livers, major causes of which include chronic viral hepatitis infection, alcohol consumption, and metabolic dysfunction-associated steatotic liver disease (MASLD).^2^ The prognosis of patients with HCC depends on the size and malignancy grade of the tumor and whether it has metastasized locally in the liver or to adjacent tissues and organs, as well as the severity of underlying liver disease.^3^ Overall, the 5-year survival rate in patients with HCC is <20%.^2^

In addition to environmental factors, genetics also has an important role in HCC.^4^ Several rare monogenic diseases confer a substantially higher risk of HCC, including α-1 antitrypsin deficiency, hemochromatosis, and glycogen storage disease, among others.^4^ Moreover, common genetic variants have been linked to HCC through genome-wide association studies (GWAS) conducted over the past decade.[5], [6], [7], [8] The most recent and largest of these, which included 1,872 HCC cases and 2,907 controls in the discovery cohort, identified variants in five different genetic regions to be associated with HCC.^5^ However, the number of common genetic variants found to be associated with the risk of HCC remains low compared with the multitude of risk variants identified for other cancer types.^9^

A better understanding of the genetic factors that affect the risk of HCC could yield new insights into the pathology of the disease. In addition, whether genetic variants, individually or combined into polygenic risk scores (PRS), can be used to predict the onset or prognosis of HCC is a topic of major clinical interest.[10], [11], [12]

In this study, we aimed to identify genetic associations with HCC through GWAS meta-analysis of 11 cohorts, which, in total, included 6,540 cases with HCC and 2.1 million controls.

## Methods

### Cohorts, association testing, and meta-analysis

Cases were defined using hospital or registry records (International Classification of Diseases [ICD]-9 or ICD-10). Controls were defined as individuals without a known history of HCC. A full description of the cohorts and case and control definitions is provided in the supplementary data online and Table S1. Details on genotyping methods are also provided in Table S1. Each study performed a GWAS of HCC using logistic regression with at least age (or year of birth), sex, and principal components (PCs) used as covariates. We conducted three fixed-effect inverse-variance weighted (IVW) meta-analyses using METAL. The first included individuals of European ancestry from nine studies, the second from two cohorts of East Asian ancestry, and, finally, a cross-ancestry meta-analysis. Genomic inflation factors were calculated for each cohort and the full meta-analyses. Genome-wide significance was set at *p* <5x10^-8^.

### Validation

To validate our findings in independent cohorts, we looked up associations with HCC in the publicly available data from the Million Veteran Program (MVP) cohort^13^ and the Taiwan Precision Medicine Initiative (TPMI) cohort.^14^ Variants identified in the European ancestry GWAS were sought validated in European ancestry participants from MVP, and variants identified in the East Asian ancestry GWAS were sought validated in TPMI. Summary statistics for the outcome ‘Phe_155_1: Malignant neoplasm of liver, primary’ in MVP were from https://ftp.ncbi.nlm.nih.gov/dbgap/studies/phs002453/analyses/GIA/. Associations for the same outcome were looked up in the TPMI (https://pheweb.ibms.sinica.edu.tw/). All GWAS-identified variants except rs12971396 were available. A proxy variant in high linkage disequilibrium (rs1042434, r^2^ = 0.94 with rs12971396) was used for validating rs12971396 in TPMI. Successful validation was defined as *p* <0.05 and consistent direction of effect with that observed in the discovery GWAS meta-analysis.

### Concordance between genetic effects on steatosis, cirrhosis, biliary tract cancer, and HCC

Steatotic liver disease and cirrhosis are known risk factors for HCC, while biliary tract cancer is located within or in direct connection with the liver. Intrahepatic biliary tract cancer is strongly associated with cirrhosis.^15^ We investigated the concordance of variant effects between steatosis, cirrhosis, biliary tract cancer, and HCC. For steatosis, we used GWAS results on MRI-measured proton density fat fraction (PDFF) from European ancestry participants in the UK Biobank (UKB).^16^ For cirrhosis, we used data from a recent cirrhosis cross-ancestry GWAS meta-analysis.^17^ In each GWAS, the beta-coefficients and 95% CIs for lead single nucleotide polymorphisms (SNPs) were extracted. When the GWASs and HCC results collectively had multiple SNPs at a locus, we included the lead SNP from the HCC GWAS. If a variant was missing from one of the GWASs, we used a proxy in high linkage disequilibrium (LD; r^2^ >0.8) when available. No GWAS exists for biliary tract cancer. Instead, we extracted genetic associations with biliary tract cancer in European ancestry participants from UKB, defined by ICD10 codes C22.1 (intrahepatic cholangiocarcinoma), C23 (cancer of gallbladder), C24 (cancer of the extrahepatic bile duct), or ICD9 codes 1551 (intrahepatic cholangiocarcinoma), or 1561/1562/1568/1569/1560 (cancer of gallbladder or extrahepatic bile duct). The effect of each HCC variant on risk of biliary tract cancer was attained through logistic regression adjusted for age, sex, and PCs 1–10. The effects of the genetic variants on steatosis, cirrhosis, and biliary tract cancer were then plotted against their effects on HCC. Heterogeneity of effects was assessed using Cochran’s Q. To provide more detail on potential biological reasons underlying the discordant and concordant effects, we examined the cellular expression patterns of the HCC-associated genes in publicly available single cell RNA-seq data from five human livers.^18^

### Genetic effects on incident HCC in at-risk subgroups

To assess the effect of the genetic variants identified in the HCC GWAS in individuals with different prevalent risk factors for cirrhosis and/or HCC, we selected individuals of white European ancestry with prevalent obesity (BMI >30 kg/m^2^), high alcohol intake (>21/14 units per week in men/women), type 2 diabetes (ICD10: E11, ICD9: 250), cirrhosis (ICD10: K70.3, K4.6; ICD9: 5712, 5715), chronic HBV (CHB) or HCV (ICD10: B17.0, B17.1, B18.2; ICD9: 07021, 07022, 07023, 07031, 07032, 07033, 07041, 07042, 07044, 07051, 07052, 07054, 07070, 07071), or chronic HCV (ICD10: B17.1, B18.2; ICD9: 07041, 07044, 07051, 07054, 07070, 07071) at the time of inclusion into the UKB. In each of these subgroups, Cox regression (adjusted for sex, age, and 10 PCs) was used to test associations of the eight variants identified in the European ancestry HCC GWAS.

### Mendelian randomization

We investigated the potential causal role of 37 plasma biomarkers, as well as BMI and alcohol intake, on the risk of HCC. To avoid overlapping samples, we conducted a meta-analysis on all available HCC cohorts except the UKB, because all the exposure traits were either completely or partly derived from the UKB. We evaluated instrument strength by calculating the F-statistic. To ensure comparable LD structure between exposure and outcome datasets, only exposures derived from samples of European ancestry were used. We selected independent variants with genome-wide significance (*p* <5x10^-8^), *r*^2^ <0.001 and non-missing rs-identifiers to serve as instrumental variables (IVs) for our MR analyses using the clumping method internal to the TwoSampleMR software and LD estimates from the European samples from the 1000G project.^19^^,^^20^ We used two different Mendelian randomization (MR) methods: the IVW model as our primary model and the weighted median model as a sensitivity analysis. MR-Egger-intercept was used to test for pleiotropy. To test whether the results were driven by individual variants, we conducted leave-one-out analyses. Only associations that passed *p* <1.3x10^-3^ (0.05/39 traits) in the primary analyses (IVW) and had a *p* <0.05 in our sensitivity analysis (weighted median) were considered significant. We performed multivariable MR (MVMR) to further investigate significantly associated biomarkers, aiming to assess whether the association might be secondary to cirrhosis. In the MVMR analysis, cirrhosis was included as an exposure alongside the biomarker, and we calculated the conditional F-statistic, Q-statistic, and IVW-estimate adjusting for sample overlap in exposures with a phenotypic correlation matrix estimated from summary statistics.^21^ The MVMR IVW-estimate is the remaining effect of the biomarker on HCC when accounting for cirrhosis.

### Polygenic risk scores

We generated a PRS to investigate its potential to identify individuals at higher risk of progressing from cirrhosis to HCC. We also investigated whether individuals with HCC and a high PRS had a worse prognosis compared with those with a lower PRS. We created a weighted score based on the eight variants that reached genome-wide significance in the European ancestry meta-analysis. The PRS was weighted using effect estimates derived from meta-analysis excluding the CHB cohort, in which we evaluated the PRS. We used two models to evaluate disease progression: (1) from cirrhosis to HCC; and (2) from HCC to death from HCC. For each model, we estimated 10-year risks using Fine-Gray regression, which accounts for the competing risk of death from non-liver cancer causes. Time zero corresponded to the first occurrence of the exposure, and individual follow-up time ended in case of the event of interest, death, or end of follow-up.

### Gene expression analyses

We analyzed transcriptomic data from HCC and non-tumor liver tissues using publicly available data from the Cancer Genome Atlas Program Liver Hepatocellular Carcinoma (TCGA-LIHC) cohort^22^ and a cohort of HCC cases from China, referred to here as GSE14520.^23^ For the TCGA-LIHC cohort, we downloaded level 3 RNA-seq data from Broad GDAC Firehose (https://gdac.broadinstitute.org/). Normalized transcriptome data calculated by RNA-Seq by Expectation Maximization (RSEM) software were available for 373 tumor and 50 non-tumor tissues collected during surgical resection in the USA. Clinical data were downloaded from cBioPortal.^24^ Disease-free and overall survival data were available for 313 and 365 patients, respectively. For the GSE14520 cohort, normalized expression array (Affymetrix) data were downloaded from the Gene Expression Omnibus.^23^ Probes were collapsed to individual gene values based on median signal intensity, quantified in arbitrary units. Transcriptome data were available for 225 tumor and 220 paired non-tumor hepatic tissues collected during surgical resection in a predominantly Asian population. Disease-free and overall survival data were available for 221 patients. Differentially expressed genes between groups were identified using Wilcoxon rank-sum test with continuity correction. Tumors were stratified into high (>median) and low (≤median) gene expression groups. Associations with disease-free and overall survival were determined by log-rank test, with visualization as Kaplan-Meier curves (‘survival’ and ‘survminer’ packages in R).

## Results

### Genome-wide association results

**Fig. 1:**
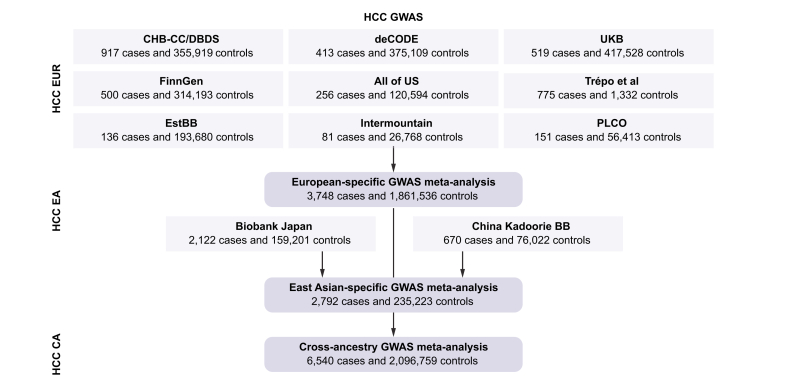
Overview of the study design. CA, cross-ancestry; CHB-CC/DBDS, Copenhagen Hospital Biobank Cancer Cohort and Danish Blood Donor Study; EA, East Asian ancestry; EUR, European ancestry; HCC, hepatocellular carcinoma; UKB, UK Biobank; EstBB, Estonian Biobank; PLCO, The Prostate, Lung, Colorectal and Ovarian Cancer Screening Trial.

**Fig. 2:**
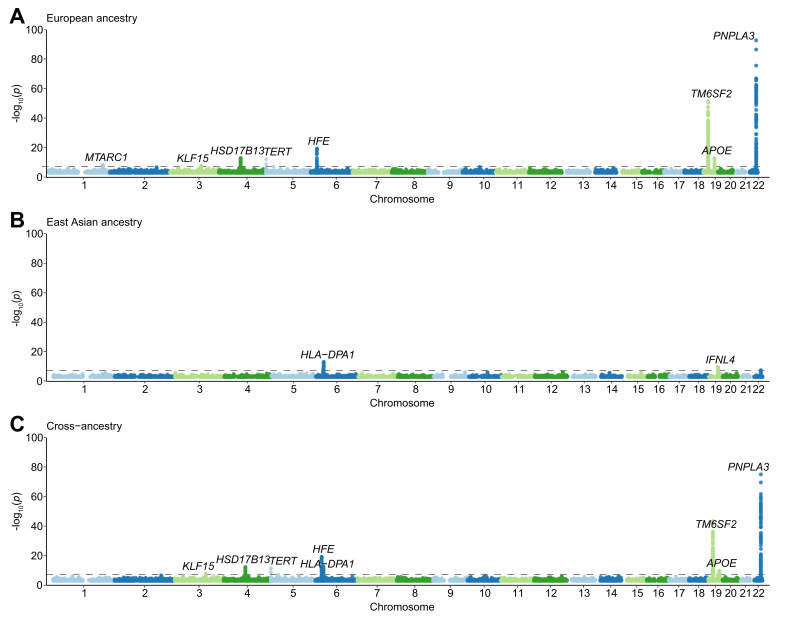
Manhattan plots for HCC GWAS meta-analyses in individuals with European ancestry (A), East Asian ancestry (B), and cross-ancestry (C). The dashed horizontal lines depict the threshold for genome-wide significance, *p* <5x10^-8^. GWAS, genome-wide association study; HCC, hepatocellular carcinoma.

We included nine European ancestry GWAS and two East Asian ancestry GWAS in our meta-analyses (Fig. 1). Baseline characteristics of cases from the included studies are shown in Table S2. In the European ancestry GWAS meta-analysis (n = 9 studies, 3,748 cases, and 1,861,536 controls), we identified eight genome-wide significant variants (Fig. 2 and Table 1), of which five (*KLF15*, *HFE*, *HSD17B13*, *APOE*, and *MTARC1*) have not previously been implicated in HCC at genome-wide statistical significance. In the East Asian ancestry meta-analysis (n = 2 studies, 2,792 cases, and 235,223 controls), we identified two variants that reached genome-wide significance (in or near *HLA-DPA1* and *IFNL4*). A cross-ancestry meta-analysis that combined all studies (6,540 cases and 2,096,759 controls) did not yield any additional loci. We observed no evidence of genomic inflation in the European or East Asian ancestry meta-analyses (*λ*_GC_EUR_ 1.03 and *λ*_GC_EA_ 1.00). We found that the SNP-based heritability estimates in Europeans and Asians were 3.4% (SE: 1.1%) and 0.7% (SE: 1.0%), respectively.

**Table 1:** Risk loci for HCC identified in the GWAS meta-analyses.

Chr	Position	Gene	Location	rs number	EA	NEA	Beta	SE	*p* value
**European ancestry GWAS meta-analysis of 3,748 cases and 1,861,536 controls**									
1	220973563	*MTARC1*	Intronic	rs2642442	T	C	0.1735	0.0295	3.98E^-09^
3	126095012	*KLF15*	Intergenic	rs7628416	C	G	0.1701	0.0302	1.77E^-08^
4	88221345	*HSD17B13*	Intergenic	rs4089	C	G	0.2121	0.0286	1.29E^-13^
5	1279790	*TERT*	Intronic	rs10069690	T	C	-0.2085	0.029	6.88E^-13^
6	26072992	*HFE*	Intergenic	rs144861591	T	C	0.4533	0.0494	4.37E^-20^
19	19379549	*TM6SF2*	Exonic	rs58542926	T	C	0.6074	0.04	3.36E^-52^
19	45411941	*APOE*	Exonic	rs429358	T	C	0.2691	0.0362	1.07E^-13^
22	44324730	*PNPLA3*	Exonic	rs738408	T	C	0.5538	0.027	2.69E^-93^

**East Asian ancestry GWAS meta-analysis of 2,792 cases and 235,223 controls**									
6	33035974	*HLA-DPA1*	UTR3	rs3179778	A	G	0.2084	0.0281	1.34E^-13^
19	39737866	*IFNL4*	Exonic	rs12971396	C	G	0.314	0.05	3.40E^-10^

Positions are on human genome build 37 (GRCh37). Chr, chromosome; EA, effect allele; GWAS, genome-wide association study; HCC, hepatocellular carcinoma; NEA, non-effect allele.

Of the 10 unique variants identified in the European or East Asian specific meta-analysis, eight were mainly driven by associations in Europeans, whereas two were only observed in East Asians (Table 1). The lead variant rs144861591 at the *HFE* locus was nearly 100-fold more common in European populations compared with individuals of East Asian ancestry (Table S3). The variant rs12971396 in *IFNL4* is in LD (*r*^*2*^ = 1.0 in East Asian populations) with another variant at the same locus (rs12979860) that has been strongly associated with chronic HCV infection, a main driver of both cirrhosis and HCC in East Asia.^25^ *IFNL4* rs12971396 was associated with HCC in Biobank Japan (*p* = 2.9x10^-12^) but not in the China Kadoorie Biobank (*p* = 0.88).^26^ The lead variants at *PNPLA3*, *TM6SF2*, and *APOE* were either coding or in high LD with a coding variant and have all been previously implicated in the full spectrum of MASLD, including cirrhosis and HCC.^8^^,^^11^ The lead *TERT*-variant rs10069690 is in LD (*r*^*2*^ = 0.64 in Europeans) with rs2242652, a variant that has previously been associated with HCC.^8^ The rs2242652-variant was also associated with HCC in our study (*p* = 2.1x10^-9^; Table S4). We also re-evaluated eight variants that have been associated with HCC in previous GWAS or candidate gene studies and not detected in the present GWAS (Table S4). Of these eight variants, three were associated with HCC: *SERPINA1* rs28929474 (*p* = 5.5x10^-7^), *MBOAT7* rs641738 (*p* = 1.5x10^-4^), and *HLA-DQB1* rs9275224 (*p* = 6.3x10^-3^).

### Validation

A total of 10 loci were identified through HCC GWAS meta-analyses. We sought to validate the associations in independent cohorts, namely the MVP (n = 2,852 cases and 447,587 controls) for the eight loci identified in European ancestry GWAS, and the TPMI cohort (n = 4,778 cases and 286,102 controls) for the two loci identified in individuals of East Asian ancestry. Of the 10 associations in total, all except the one near *KLF15* were replicated with consistent direction of effect and *p* <0.05 (Table S5). Seven of the 10 associations (all but *KLF15*, *MTARC1*, and *IFNL4*) were replicated with a more stringent Bonferroni corrected threshold of *p* <0.005 (0.05/10 tested variants).

### Comparison of genetic effects on steatosis, cirrhosis, biliary tract cancer, and HCC

**Fig. 3:**
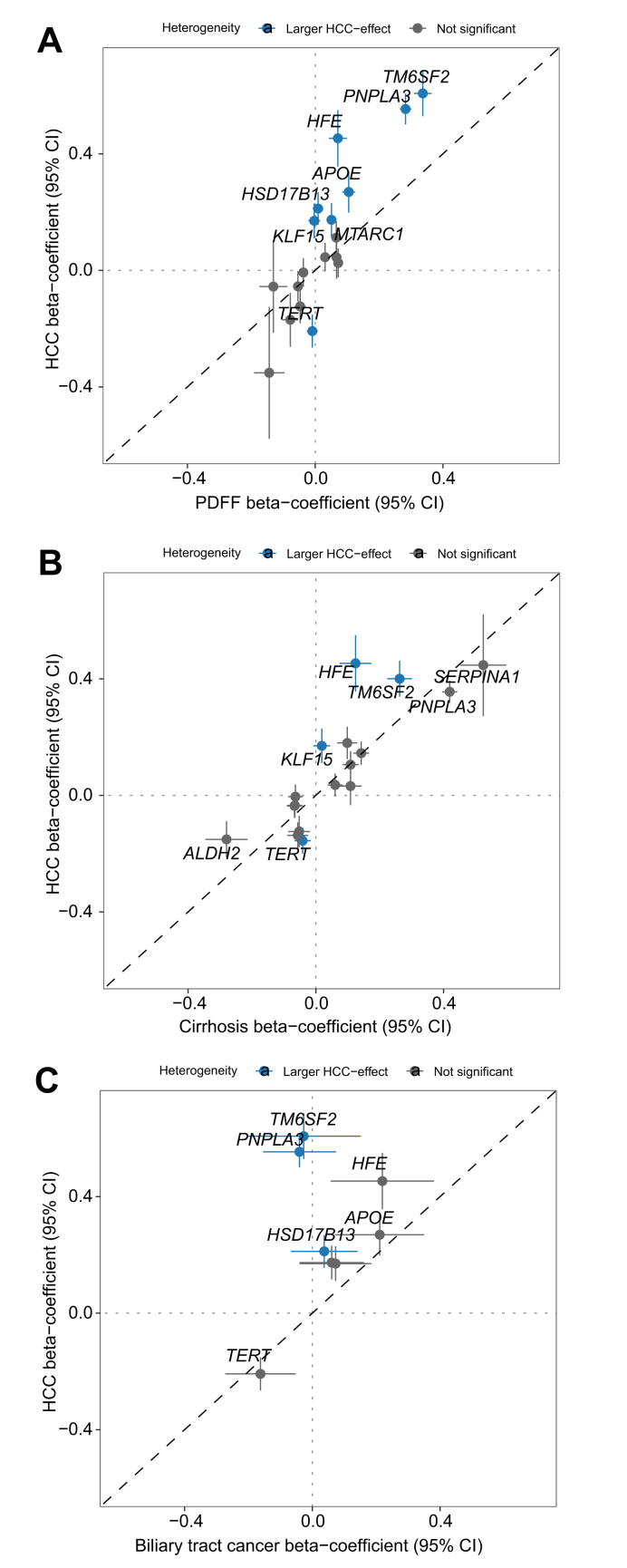
Comparison of genetic associations with HCC, hepatic steatosis, cirrhosis, and biliary tract cancer. Variants that had stronger effects (*p* <0.05 by Cochran’s Q test) on HCC compared with steatosis, cirrhosis, or biliary tract cancer, respectively, are in blue. In all analyses, HCC effects were derived from the cross-ancestry meta-analysis in the present study. (A) Effects of 15 previously reported hepatic steatosis variants and eight HCC variants identified in this study, totaling 18 distinct signals. The proton density fat fraction (PDFF) effects were derived from hepatic magnetic resonance imaging (MRI) in UKB.^16^ (B) Effects of 15 previously reported cirrhosis variants and 10 HCC variants from this study, totaling 16 distinct signals. The cirrhosis effects were derived from a previous GWAS.^17^ (C) Effects of eight HCC variants identified in the present study. Effects on biliary tract cancer were derived from European-ancestry UKB participants (893 cases and 458,134 controls). The dashed identity line (*y*  =  *x*) is shown for reference in each panel. GWAS, genome-wide association study; HCC, hepatocellular carcinoma; UKB, UK Biobank.

Among 18 distinct variants (15 previously associated with steatosis, eight associated with HCC in European-ancestry individuals), we found a strong correlation (*r*^*2*^ = 0.75, *p* = 2x10^-6^) between effects on steatosis and HCC (Fig. 3A). Concordantly, using a set of 16 unique variants previously associated with cirrhosis (15 variants) and HCC (10 variants) in cross-ancestry analyses, we observed a strong correlation (*r*^*2*^ = 0.69, *p* = 2x10^-5^, Fig. 3B). We observed some notable differences in variant effects. For instance, five variants in or near *TM6SF2*, *PNPLA3*, *HFE*, *APOE*, and *MTARC1* had comparably larger effects on HCC than on hepatic steatosis (*p* <0.05 by Cochran’s Q test), while HCC-associated variants in *HSD17B13, KLF15,* and *TERT* did not significantly associate with steatosis. In the comparison between cirrhosis and HCC, we found that variants in *HFE, TM6SF2,* and *TERT* displayed larger effects on HCC compared with their effects on cirrhosis, while the *KLF15* variant had no effect on cirrhosis and only affected the risk of HCC. There was no significant overlap between genetic effects on HCC and biliary tract cancer (r^2^ = -0.035, *p* = 0.42), with three exceptions, namely *TERT*, *HFE*, and *APOE*, which all had concordant effects on both HCC and biliary tract cancer (*p* = 0.004, 0.008, and 0.003, respectively, for association with biliary tract cancer) (Fig. 3C). To shed more light on the discordant and concordant effects between HCC and biliary tract cancer, we analyzed single cell RNA-seq data from five human livers (Table S6). No clear differences in cellular expression patterns emerged. *APOE,* which affects both HCC and biliary tract cancer, was primarily expressed in hepatocytes. *TERT* and *HFE*, which also affect both cancers, were expressed at low levels across hepatic cell types.

### Genetic effects on incident HCC in at-risk subgroups

We sought to assess whether the effect of the genetic variants on HCC differed by underlying risk factors (Table S7). Each of the eight variants identified in the European ancestry HCC GWAS was tested for association with incident HCC in UKB participants with prevalent obesity, high alcohol consumption, type 2 diabetes, cirrhosis, or chronic hepatitis. Across the 40 tests (eight variants tested in five subgroups), there was a high (35/40 or 88%) concordance of effects with those seen in the GWAS. Of the 40 tests, 21 were nominally statistically significant (*p* <0.05) and nine were significant using a more stringent Bonferroni-adjusted threshold of 0.001 (0.05/40 tests). The magnitude of the effect estimates tended to be larger in the at-risk subgroups compared with the GWAS. Of the 40 tested associations, 27 (68%) had numerically higher effects than the GWAS estimate for that variant. Associations in individuals with hepatitis C specifically were similar to those seen for any chronic hepatitis (Table S7).

### Mendelian randomization

We used MR to evaluate the potential causal effects of a range of biochemical, metabolic, and behavioral exposures on the risk of HCC. We first tested a panel of 37 blood biomarkers (Fig. S1). Of these, increasing levels of alanine transaminase (ALT), aspartate transaminase (AST), and gamma-glutamyl transferase (GGT) were associated with HCC (*p* <1.3x10^-3^ [0.05/39 tests]), but ALT and AST showed evidence of pleiotropy (Egger intercept *p* <0.05). Cirrhosis explained most of the effects of ALT, AST, and GGT on HCC, with overall strong attenuation in effect estimates when accounting for the genetic effects of cirrhosis in MVMR analyses (Table S8).

**Fig. 4:**
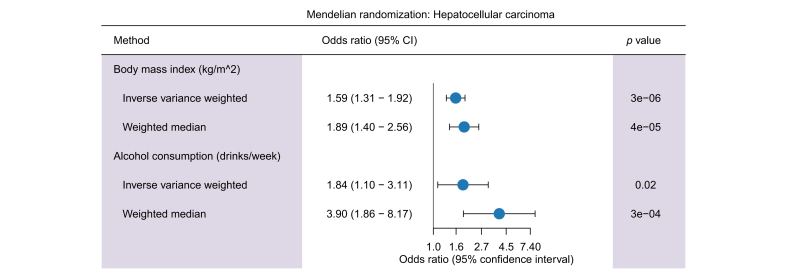
Mendelian randomization analyses of the effect of BMI and alcohol intake on HCC. The HCC odds ratios are for a genetically proxied 1-SD increase in BMI (top) or alcohol consumption (bottom), respectively. HCC, hepatocellular carcinoma.

We then tested the effect of metabolic and behavioral exposures on the risk of HCC. A higher BMI increased the risk of HCC (Fig. 4). We also found a nominally significant association between alcohol intake and risk of HCC (*p* = 0.03). In sensitivity analyses, the association with alcohol was found to be largely driven by *AHD1B* rs1229984, which affects alcohol intake, but with consistent direction of effects after exclusion of the variant (Fig. S2). Variant-level effect plots for ALT, AST, GGT, BMI, and alcohol are shown in Fig. S3.

### Polygenic risk scores and HCC

We evaluated whether a PRS could aid in identifying individuals with cirrhosis who were more likely to progress to HCC, as well as those with poorer survival once diagnosed with HCC. Among 4,258 individuals with cirrhosis in CHB, 315 developed HCC during follow-up. A higher PRS was associated with an increased risk of HCC after the diagnosis of cirrhosis. Individuals with cirrhosis and a high PRS (top 20%) had a 10-year HCC risk of 13.0% (95% CI 11.0–15.0) compared with 6.9% (95% CI 6.0–7.8; *p* for difference <0.001) for individuals in the bottom 80% of the PRS. However, we found no significant association between a higher PRS and HCC survival. Among 918 individuals with HCC, 395 died from HCC during follow-up, with individuals in the top 20% of the PRS having a similar 10-year risk (53.0%, 95% CI 45.0–61.0) to those in the bottom 80% (48.0%, 95% CI 44.0–52.0; *p* for difference = 0.60).

### Gene expression analyses

We sought to determine whether expression of genes harboring or located near HCC-associated SNPs was affected in HCC, and whether transcriptional activity of these genes may yield prognostic value. To assess this, we conducted analyses of transcriptome data of liver tissue from two cohorts of patients with HCC, totaling 373 tumors and 50 non-tumoral tissues, and 225 tumors and 220 non-tumoral tissues, respectively.^22^^,^^23^ First, we assessed differences in baseline expression levels of the nine genes with available data (all but *IFNL4*). *HSD17B13*, *TM6SF2*, *PNPLA3*, *MTARC1*, and *HLA-DP1* exhibited lower expression in HCC compared with non-tumor liver tissues (Fig. S4). Second, we tested whether the overall or disease-free survival differed by transcriptional level. The only significant association was seen for *HSD17B13,* for which RNA levels below compared with above the median were associated with lower overall survival (Bonferroni corrected *p* <0.005; Figs S5 and S6). For comparison, we also tested a panel of 541 genes with liver-specific expression and found that these were, on average, substantially downregulated in HCC compared with normal liver tissue (Fig. S7).

## Discussion

This study identified 10 variants associated with HCC based on data from GWAS meta-analyses including more than 6,500 cases and 2 million controls. Nine of the associations were validated in independent cohorts totaling 7,630 cases and 733,689 controls. Most of the risk variants were found to also associate with the risk of cirrhosis, a major predisposing factor for the development of HCC. The identified variants had comparable effects on incident HCC in individuals with prevalent obesity, a high alcohol intake, diabetes, or cirrhosis.

Our findings align with those from previous HCC GWASs. The most recent of these studies included 1,872 HCC cases and 2,907 controls of European descent and identified five loci in or near *PNPLA3*, *TM6SF2*, *TERT*, *HLA*, and *MOBP* to be associated with HCC.^5^ Another study of 1,866 HCC cases and 197,745 controls from Biobank Japan identified an association at the *IFNL4* locus.^26^ We replicated associations in *PNPLA3*, *TM6SF2*, *TERT*, *HLA*, and *IFNL4*, but could not confirm associations at the *MOBP* locus. We also report five variants near genes that have not previously reached genome-wide significance for association with HCC: *KLF15*, *HFE*, *HSD17B13*, *APOE*, and *MTARC1*. Finally, we confirm associations with HCC for the candidate genes *SERPINA1* and *MBOAT7*.

The lead variant at the *HFE* locus is in perfect LD (*r*^*2*^ = 1.0) with *HFE* p.Cys282Tyr, the most common cause of hemochromatosis and a known risk factor for HCC.^27^ In other words, although *HFE* has not been implicated in previous HCC GWASs, it should be viewed as a known risk locus for this cancer. The variant at *HSD17B13* affects the risk of steatohepatitis and cirrhosis and has also previously been associated with HCC, albeit at sub-genome-wide significance.^7^^,^^28^ Variation at *APOE*, *MTARC1,* and *MBOAT7* has been found to confer risk of hepatic steatosis and cirrhosis.^29^^,^^30^ The effects of these loci on HCC are likely secondary to their effects on cirrhosis. The *SERPINA1* variant is a known cause of α-1 antitrypsin deficiency, which confers a higher risk of cirrhosis and, in turn, HCC. The lead variant at *TERT*, rs10069690, differs from the lead variant in previous studies, rs2242652. While the two variants are in moderate linkage (r^2^ = 0.62), recent functional studies indicate that they exert distinct biological effects, with rs10069690 (but not rs2242652) causing a change in *TERT* splicing.^31^ The association between the *IFNL4* locus and HCC was only observed in the East Asian samples from Biobank Japan.^26^ A potential reason for this is that *IFNL4* mediates its risk-increasing effect on HCC via its primary effect on increased susceptibility to chronic HCV.^25^ Approximately 50% of the HCC cases in Biobank Japan were positive for HCV, whereas HBV was the predominant form of hepatitis in cases from the China Kadoorie Biobank. Intriguingly, Hassan *et al.* did not detect associations with *IFNL4* in their recent HCC GWAS of non-Hispanic white Americans, despite 39% of the HCC cases in that study having HCV.^5^ Therefore, it remains possible that the *IFNL4* locus exerts effects on HCC that are distinct from its effects on HCV. Other studies have reported complex relationships between *IFNL4* and liver disease. Eslam *et al.*^32^ found that *IFNL4* variants associated with higher IFN treatment response rates were also associated with increased inflammation and fibrosis regardless of liver disease etiology. In other words, *IFNL4* variants that increase HCV clearance may also increase fibrosis. Thus, the risk-increasing allele may differ depending on what outcome the study is focusing on. *KLF15* encodes Kruppel-like factor 15, a transcription factor that is highly expressed in liver tissue.^33^^,^^34^ The *KLF15* association was not replicated in the MVP cohort and, therefore, should be viewed as preliminary, requiring replication in other cohorts. Taken together, the GWAS associations reported here highlight the importance of MASLD, cirrhosis, hemochromatosis, α-1 antitrypsin deficiency, and chronic viral hepatitis in the development of HCC.

The genetic effects on steatosis and cirrhosis were generally concordant with their effects on HCC, with some heterogeneity. For example, *TM6SF2* and *HFE* had larger effects on HCC than on steatosis and cirrhosis, while *TERT* and *KLF15* had no effects on steatosis and only small or no effects on cirrhosis. By contrast, there was remarkably little overlap between effects on HCC and biliary tract cancer.

The two loci with the strongest effects on HCC, *PNPLA3* and *TM6SF2*, did not show any evidence of association with biliary tract cancer. Overall, there was no correlation between genetic effects on HCC and cholangiocarcinoma. Interestingly, the variants in *HFE*, *APOE*, and *TERT* displayed concordant effects on HCC and biliary tract cancer. The lead variant at the *HFE* locus is a known cause of hemochromatosis, a disorder that has been associated with both HCC and biliary tract cancer.^35^ *APOE* encodes an apolipoprotein that binds to circulating very low-density lipoprotein (VLDL) cholesterol. *TERT* encodes telomerase, which is known to have a key role in carcinogenesis across tumor types. TERT, and telomeres, has been implicated in both HCC and biliary tract cancer in previous studies.[36], [37], [38]

Most HCC cases develop in cirrhotic livers, major causes of which include chronic viral hepatitis, a high intake of alcohol, and MASLD. We found that the risk variants had effects on incident HCC that were similar in individuals with cirrhosis, obesity, type 2 diabetes, or a high alcohol intake. The variants were not associated with HCC in chronic viral hepatitis cases, but the small sample size of this subgroup limited the statistical power. The magnitude of the effects on HCC tended to be larger in these at-risk subgroups than in the overall GWAS meta-analyses, in alignment with previous findings of gene–environment synergistic effects influencing the entire spectrum of MASLD, including HCC.^17^

MR analyses supported a causal effect of adiposity on higher risk of HCC. Although not reaching the threshold for statistical significance, the estimates for alcohol intake were also consistent with a causal, risk-increasing effect on HCC. Moreover, we found that genetically proxied higher liver enzymes were associated with a higher risk of HCC. However, when accounting for the genetic effects of liver cirrhosis, the associations of liver enzymes with HCC were substantially attenuated. These findings underline that plasma liver enzymes *per se* do not cause HCC, but that the association is driven by chronic liver disease. We did not detect evidence supporting a causal association with HCC for a range of other circulating biomarkers, including plasma lipids and lipoproteins, C-reactive protein, and glucose.

We found that individuals with cirrhosis and a PRS in the top 20% had a twofold higher risk of HCC compared with a PRS in the lowest 80%. Whether patients with cirrhosis and a high PRS should undergo screening for HCC at shorter intervals compared with patients with similar environmental risk factors and a low PRS warrants further investigation.

Several of the genes identified in the present GWAS had lower transcriptional activity in HCC tissue than in non-tumor hepatic tissues. This was most notable for *HSD17B13*. Other genes with lower expression in HCC included *MTARC1*, *TM6SF2*, and *PNPLA3*. It is unclear whether these associations reflect causality. We speculate that at least some of the associations reflect that HCC leads to loss of normal hepatocyte function. Of the downregulated genes, *HSD17B13* is exclusively expressed in the liver, while *PNPLA3*, *MTARC1*, and *TM6SF2* have high liver expression. All four genes have a role in hepatic lipid metabolism. The HCC-associated downregulation of these four genes is likely to reflect loss of normal metabolic functions due to the dedifferentiation of hepatocytes to cancer cells in HCC. In support of this notion, we found that a panel of 541 liver-specific genes had substantially lower transcriptional activity in HCC compared with normal liver tissue.

Our study has limitations that should be considered. First, the heterogenous nature of the included cohorts and HCC cases is a limitation. This was particularly evident in the comparison between European-ancestry and Asian cohorts, where MASLD and alcohol were major risk factors in the former, while chronic hepatitis predominated in the latter. This fundamental difference was reflected in the HCC-associated genetic loci identified, with established MASLD loci enriched in Europeans and loci associated with susceptibility to chronic viral hepatitis enriched in Asians. We did not have access to information about antiviral treatment status for the participants with chronic hepatitis. Given that participants in the cohorts were enrolled from ∼2000 to 2025, and that cases could be defined retrospectively via registry codes, some of the chronic hepatitis cases are likely to have been diagnosed before the advent of modern antiviral drugs. Another source of heterogeneity is that the designs of the cohorts ranged from prospective general population cohorts to cross-sectional hospital-based cohorts to case-control studies. A limitation of the risk-group analyses in the UKB was that some of the subgroups were relatively small, limiting statistical power. This was most notable for the chronic hepatitis subgroup (n = 202). The lack of association between risk alleles and increased HCC risk in patients with viral hepatitis should be interpreted cautiously and may reflect insufficient power, rather than true heterogeneity. However, Hassan *et al.* also found that variants in *TM6SF2* and *PNPLA3* were not significantly associated with HCC in patients with HCV.^5^ Misclassification of cases and controls is an inherent limitation to ICD-defined case definitions. Moreover, individuals defined as controls in the prospective cohorts may have developed HCC if followed for longer. These limitations related to misclassification are likely minor, considering that HCC is a hard clinical endpoint with well-defined diagnostic criteria and the long follow-up in the included cohorts (*i.e.* UKB currently has a median follow-up of 14 years). In any case, misclassification would bias associations toward the null hypothesis and, thus, cannot explain the positive associations reported here. Finally, while our GWAS represents one of the largest HCC GWASs to date, the number of HCC cases, although substantial, still limits the statistical power to detect variants with smaller effects, as well as the effects of rare variants. Therefore, future studies with larger sample sizes are required.^39^ Notably, the overall heritability of HCC remains unclear. Hassan *et al.* reported heritability estimates of HCV-negative and HCV-positive HCC of 26% and 30%, respectively.^5^ Another study reported an estimated heritability of 6.3% in HBV-associated HCC.^40^ Among 124 monozygotic twins of patients with HCC, none developed the disease, suggesting a modest contribution of germline genetic variation to this cancer. However, the small number of cases and absent information on shared exposures limit the interpretation of this twin study.^41^

In conclusion, we identified and validated nine genetic variants to be associated with HCC. These results expand the catalog of genes to interrogate mechanistically in future studies. A deeper insight into the genetic factors that underpin HCC may improve our ability to predict and ultimately prevent or treat this deadly cancer.

## Abbreviations

ALT, alanine transaminase; AST, aspartate transaminase; CA, cross-ancestry; CHB-CC/DBDS, Copenhagen Hospital Biobank Cancer Cohort and Danish Blood Donor Study; CHB, chronic HBV; Chr, chromosome; EA, East Asian ancestry; EA, effect allele; EstBB, Estonian Biobank; EUR, European ancestry; GGT, gamma-glutamyl transferase; GWAS, genome-wide association study; HCC, hepatocellular carcinoma; HCC, hepatocellular carcinoma; IVs, instrumental variables; IVW, inverse-variance weighted; LD, linkage disequilibrium; MASLD, metabolic dysfunction-associated steatotic liver disease; MR, Mendelian randomization; MRI, magnetic resonance imaging; MVMR, multivariable Mendelian randomization; PCs, principal components; PDFF, proton density fat fraction; PLCO, The Prostate, Lung, Colorectal and Ovarian Cancer Screening Trial; PRS, polygenic risk score; RNA-seq, RNA-sequencing; RSEM, RNA-Seq by Expectation Maximization; SNP, single nucleotide polymorphism; UKB, UK Biobank; VLDL, very low-density lipoprotein.

## Financial support

This research was conducted using the UKB resource under application 43247. This work was supported by BRIDGE—Translational Excellence Program (NNF20SA0064340 to JG), Beckett Fonden (23-2-10636 to JG), Independent Research Fund Denmark (9060-00012B to SS), The 10.13039/100012774Innovation Fund Denmark (PM Heart to HB), NordForsk (to HB), 10.13039/501100009925Villadsen Family Foundation (to HB), The 10.13039/100007460Arvid Nilsson Foundation, and 10.13039/501100009708Novo Nordisk Foundation (grants NNF17OC0027594 and NNF14CC0001 to KB and SB; NNF22OC0074956 to JBA; NNF22OC0075038 to SS). The All of Us Research Program is supported by the 10.13039/100000002National Institutes of Health (NIH), Office of the Director: Regional Medical Centers: 1 OT2 OD026549; 1 OT2 OD026554; 1 OT2 OD026557; 1 OT2 OD026556; 1 OT2 OD026550; 1 OT2 OD 026552; 1 OT2 OD026553; 1 OT2 OD026548; 1 OT2 OD026551; 1 OT2 OD026555; IAA #: AOD 16037; Federally Qualified Health Centers: HHSN 263201600085U; Data and Research Center: 5 U2C OD023196; Biobank: 1 U24 OD023121; The Participant Center: U24 OD023176; Participant Technology Systems Center: 1 U24 OD023163; Communications and Engagement: 3 OT2 OD023205; 3 OT2 OD023206; and Community Partners: 1 OT2 OD025277; 3 OT2 OD025315; 1 OT2 OD025337; 1 OT2 OD025276. The research was also supported by NIH HHS under grant 5T32GM136651-03 (to JB). This work was also supported by the NIH/NIDDK (R01 DK131033-01A1 to SV). The Estonian Biobank was funded by the European Union through the European Regional Development Fund (project 2014-2020.4.01.15-0012 GENTRANSMED) and by the Estonian Research Council (grant PRG1911). Computations were performed in the High-Performance Computing Center, University of Tartu. The funders had no role in any of the following: analysis or interpretation of data, design of the study, writing of the manuscript, or the decision to submit it for publication.

## Authors’ contributions

JG, HG, TR, HB, SS: conceived the study. JG, HG, GT, JB, CK, TL, EF, DFG, SS: performed analyses in the respective cohorts. TR, SV, TL, JBA, HB, SS: supervised analyses in their respective cohorts. JG performed meta-analyses. JG, HG, CJO, AS, SS: performed analyses and created figures and tables. JG, HG, SS: drafted the manuscript. JG, HG, CJO, AS, GT, GS, VT, JB, CK, SV, TME, GA, JSB, SAR, PRL, ES, CM, JT, CE, KMD, MTB, BAJ, JTB, SB, KB, HU, TL, RM, LDN, KUK, SK, LLG, KV, ESB, MOU, PS, HH, OBP, SRO, DFG, TR, KS, UL, HP, JGH, JBA, HB, SS: interpreted the results, and reviewed and commented on the manuscript. JG, HG, SS: directly accessed and verified the underlying data reported in the manuscript.

## Data availability statement

Summary association results will be made freely available to all others via the GWAS catalog (www.ebi.ac.uk/gwas/) upon publication.

## Conflicts of interest

The authors who are affiliated with deCODE genetics/Amgen declare competing financial interests as employees. JG has received lecture fee from Illumina. SV has served as consultant for Albireo and received research funding from Moderna Therapeutics, with no relevance to this study. SB is a board member for Proscion A/S and Intomics A/S. JBA has received consulting fees from AstraZeneca (Nordic), QED Therapeutics, and Flagship Pioneering as well as project funding from Incyte Corp and ADCendo (not related to this study). HB receives lecture fees from Bristol-Myers Squibb, Merck Sharp and Dohme. SS has served as consultant for Regeneron and received a lecture fee from Amgen. All other authors have no conflict of interest to declare.

Please refer to the accompanying ICMJE disclosure forms for further details.
