# Supplementary material for: Genome-wide meta-analysis identifies nine loci associated with higher risk of hepatocellular carcinoma development
Source: JHEP Rep. 2025 Jun 11;7(9):101485. doi: 10.1016/j.jhepr.2025.101485 (PMC12355075; doi:10.1016/j.jhepr.2025.101485)
Supplement: Multimedia component 1 [file mmc1.pdf]

Genome-wide meta-analysis identifies nine loci associated with higher risk of hepatocellular carcinoma development

Jonas Ghouse, Helene Gellert-Kristensen, Colm J. O'Rourke, Anne-Sofie Seidelin, Gudmar Thorleifsson, Gardar Sveinbjörnsson, Vinicius Tragante, Chigoziri Konkwo, Joseph Brancale, Silvia Vilarinho, Tim M. Eyrich, Gustav Ahlberg, Johan S. Bundgaard, Søren A. Rand, Pia R. Lundegaard, Erik Sørensen, Christina Mikkelsen, Jacob Træholt, Christian Erikstrup, Khoa M. Dinh, Mie T. Bruun, Bitten Aa. Jensen, Jakob T. Bay, Søren Brunak, Karina Banasik, Henrik Ullum, DBDS Genomic Consortium, Estonian Biobank Research Team, Triin Laisk, Reedik Mägi, Lincoln D. Nadauld, Kirk U. Knowlton, Stacey Knight, Lise L. Gluud, Kirsten Vistisen, Einar S. Björnsson, Magnus O. Ulfarsson, Patrick Sulem, Hilma Holm, Ole B. Pedersen, Sisse R. Ostrowski, Daniel F. Gudbjartsson, Thorunn Rafnar, Kari Stefansson, Ulrik Lassen, Hans-Christian Pommergaard, Jens G. Hillingsø, Jesper B. Andersen, Henning Bundgaard, Stefan Stender

Table of contents

Supplementary materials and methods..2

Supplementary tables..6

Supplementary figures..14

Supplementary references...21

Consortia members...23

Supplementary materials and methods

Cohort information, case-control ascertainment, ethics and genotyping and imputation details

Copenhagen Hospital Biobank Cancer Cohort (CHB-CC) and Danish Blood Donor Study (DBDS)

Case-control ascertainment: The Copenhagen Hospital Biobank (CHB) Cancer Cohort (CHB-CC) includes genome-wide genotype data for ~319,000 subjects admitted to general hospitals in the capital area of Denmark between 2009 and 2020[1]. The Danish Blood Donor Study is a cohort including blood donors from Denmark.[2] As of 2024, ~114,000 individuals with genome-wide genotype data were available. HCC cases were defined using the following ICD-10 codes: C22.0 (Liver cell carcinoma) or C22.9 (Malignant neoplasm of liver, not specified as primary or secondary).

Ethics: Since the biological samples stored in CHB-CC were based on leftover material from routine blood analyses, the patients were not asked for informed consent before inclusion. However, patients were informed by electronic mail (E-Boks) or normal mail about the research and the opt-out possibility to have their biological specimens excluded from use in research in the research conducted or in general. Thus, since 2004 a national Register on Tissue Application (Vævsanvendelsesregistret) lists all individuals who have chosen to opt out and whose samples cannot be used for research purposes. Before initiating this study, individuals listed in the Register on Tissue Application were excluded. For DBDS, informed consent was obtained from all participants. Both CHB- CC and DBDS are approved by the National Committee on Health Research Ethics (NVC 1708829 and NVC 1700407) and the Danish Data Protection Agency (P-2019-93 and P-2019-99).

Genotyping and imputation: Samples from 276,114 Danes from the CHB and DBDS were genotyped using Illumina Global Screening Array chips and long-range phased together with ~238,000 genotyped samples from North-western Europe using Eagle. Samples and variants with less than 98% yield were excluded. A haplotype reference panel was prepared in the same manner as for the Icelandic data (see below) by phasing whole-genome sequence genotypes of 15,576 individuals from Scandinavia, the Netherlands, and Ireland using the phased chip data. Graphtyper was used to call the genotypes which were subsequently imputed into the phased chip data. Whole genome sequencing, chip-typing, quality control, long-range phasing, and imputation from which the data for this analysis were generated was performed at deCODE genetics.

deCODE

Case-control ascertainment: Data from the deCODE study included 413 HCC cases and 375,109 controls[3]. Cases were defined using the ICD-10 code C22.0. The information on HCC diagnosis is obtained from the Icelandic Cancer Registry (ICR).[4] In this study we utilize registered diagnoses from 1955 to 2022.

Ethics: The study was approved by the National Bioethics Committee (VSN-18-148) and the Icelandic Data Protection Authority. Written informed consent was obtained from all participants who donated blood samples to research. All sample identifiers were encrypted in accordance with the regulations of the Icelandic Data Protection Authority.

Genotyping and imputation: The genome of the Icelandic population was characterized by whole-genome sequencing of 49,708 Icelanders using Illumina standard TruSeq methodology to a mean depth of 35x (SD 8x) with subsequent long-range phasing,[5] and imputing the information into 166,281 individual chip-genotyped employing multiple Illumina platforms.[6] Using genealogic information, we further imputed sequence variants into 285,664 relatives of the genotyped individuals to increase the sample sizes and power to detect associations. In total, we characterized up to 35.3 million variants in the Icelandic population with imputation score > 0.8.

Intermountain Healthcare

Case-control ascertainment: The HerediGene Population study is a large-scale collaboration between Intermountain Healthcare, deCODE genetics, and Amgen, Inc.[3] Participants in the HerediGene Population study are voluntary US residents over the age of 18 years, who gave permission to link anonymized genotypic data with EHRs. Cases were defined using the following ICD-10 codes: C22.0. The remaining individuals of the Intermountain Healthcare cohort were included as controls. In total, 81 cases and 26,768 controls were identified.

Ethics: The Intermountain Healthcare Institutional Review Board approved this study, and all participants provided written informed consent prior to enrollment.

Genotyping and imputation: The Intermountain dataset was derived from a cohort created by whole-genome sequencing of 16,661 Americans of European ancestry living in Utah, to an average coverage of at least 20x, performed at deCODE genetics. These samples served as a reference panel for long-range phasing and imputation of 60,397 chip-typed individuals enrolled at multiple Intermountain Healthcare facilities.

UK Biobank

Case-control ascertainment: The UK Biobank (UKB) is a prospective cohort of more than 500,000 individuals living in the United Kingdom who were 40-79 years of age at recruitment (2006-2010).[7] The median age at enrollment was 58 years and median follow-up time in 2024 was ~14 years. Cases were defined using the following ICD-10 codes: C22.0. All other participants were set as controls. Data from the UKB included 519 HCC cases and 417,528 controls.

Ethics: The UK Biobank cohort has been approved by the Northwest Multicenter Research Ethics Committee, UK (Ref: 16/NW/0274). Written informed consent has been obtained from all study participants.

Genotyping and imputation: Genotypic data were available for 488,380 individuals and were imputed to the HRC, UK10K and 1,000 Genomes Phase 3 reference panels using IMPUTE4 to identify ≈ 93M variants for 487,409 individuals. Using the genotyped SNPs, persons were excluded if they had: high levels of missingness or heterozygosity, SNP genotype call rate < 98%, or if phenotypic and genotypic gender information was discordant.

Estonian Biobank

Case-control ascertainment: The Estonian Biobank is a volunteer-based sample of the Estonian adult population aged ≥18 years[8]. Baseline measurements included a standardized health examination, health-related questionnaires, blood samples for DNA, white blood cells and plasma tests, and clinical diagnoses defined by ICD-10 codes. HCC cases were defined using the following ICD-10 codes: C22.0 or C22.9 extracted from health records. Median follow-up was ~10 years in 2024.

Ethics: Analyses in the Estonian Biobank were carried out under ethical approval 1.1-12/624 from the Estonian Committee on Bioethics and Human Research (Estonian Ministry of Social Affairs), using data according to Estonian Biobank release 6-1/GI/47. All participants provided written informed consent.

Genotyping and imputation: At present, more than 200,000 participants have undergone genotyping by a genome-wide SNP array which includes more than 700,000 SNPs (Illumina GSA microchip).

FinnGen Freeze 10

Case-control ascertainment: The summary statistics for 500 cases and 314,193 controls from Finngen were downloaded from https://www.finngen.fi/en/access_results. Cases were defined using the following ICD-10 codes: ICD10: C22.0.

Ethics: Patients and control subjects in FinnGen provided informed consent for biobank research, based on the Finnish Biobank Act. Separate research cohorts, gathered before the Finnish Biobank Act was initialized (in September 2013) and start of FinnGen (August 2017), were collected based on study-specific consents and later assigned to the Finnish biobanks after approval by Fimea, the National Supervisory Authority for Welfare and Health. The Coordinating Ethics

Committee of the Hospital District of Helsinki and Uusimaa (HUS) approved the FinnGen study protocol Nr HUS/990/2017. The FinnGen study is approved by Finnish Institute for Health and Welfare (THL), approval number THL/2031/6.02.00/2017, amendments THL/1101/5.05.00/2017, THL/341/6.02.00/2018, THL/2222/6.02.00/2018, THL/283/6.02.00/2019, THL/1721/5.05.00/2019, Digital and population data service agency VRK43431/2017-3, VRK/6909/2018-3, VRK/4415/2019-3 the Social Insurance Institution (KELA) KELA 58/522/2017, KELA 131/522/2018, KELA 70/522/2019, KELA 98/522/2019, and Statistics Finland TK-53-1041-17.7

Genotyping and imputation: A custom-made FinnGen ThermoFisher Axiom array (>650,000 SNPs) was used to genotype FinnGen samples. Genotype calls were made with AxiomGT1 algorithm. Individuals with ambiguous gender, high genotype missingness (>5%), excess heterozygosity (±4 SD), and non-Finnish ancestry were excluded. Variants with high missingness (>2%), low Hardy-Weinberg equilibrium ($< 1\times10^{-6}$), or low minor allele count (<3) were excluded. High coverage (25–30×) WGS data were used to develop the Finnish population-specific SISu v3 imputation reference panel with Beagle 4.1. More than 16 million variants have been imputed (https://finngen.gitbook.io/documentation/methods/genotype-imputation).

All of US

Case-control ascertainment: A total of 256 cases with cirrhosis and 120,594 controls were included from the All o fUS cohort[9]. HCC cases were defined using SNOMED-25370001.

Ethics: Ethical approval was received from National Institute of Health All of Us Institutional Review Board. Participants provided written informed consent.

Genotyping and imputation: Genotyping was by whole genome sequencing (Illumina Whole Genome Sequencing. All of Us Research and Data Center whole genome sequencing Hail Matrix Table (v.6) was imported into Hail (v. 0.2.107). Samples were stratified by self-reported race or ethnicity into Black, Hispanic, and White subgroups. Only individuals of White European ancestry were included in our study owing to too few samples in the other subgroups. Samples were filtered for heterozygosity, sex mismatch, and genetic-ancestry outliers.

French/Belgian HCC GWAS (Trépo et al)

Case-control ascertainment: The summary statistics from a previously published GWAS[10] of 775 HCC cases and 1332 controls (all self-reported French or Belgian ancestry) were downloaded from the GWAS catalog at https://www.ebi.ac.uk/gwas/studies/GCST90092003. Cases were defined as patients with HCC based on imaging or histology on a background of alcohol-related cirrhosis. Control individuals had alcohol-related liver disease but no HCC.

Ethics: Patients gave written, informed consent and the study received approval from the ethics committees of all participating centers.

Genotyping and imputation: Genotyping was done with the Global Screening Array, version 1.0 (Illumina; San Diego, CA, USA), which contains 618,564 variants before quality control. Samples that had genetic sex discordance, genotype call rates less than 95% or outlying heterozygosity for autosomal chromosomes (i.e., ±3 SDs away from the sample mean), or had an estimated identity by descent value of more than 0·1875 were excluded. Ancestry was identified by principal component analysis, and individuals with non-European ancestry were excluded because of small numbers. SNPs with a genotype call rate lower than 95%, a minor allele frequency less than 0·1%, different missing genotype rates in cases and controls (p<10E−5), and that did not meet Hardy-Weinberg equilibrium (p<10E−6) in controls were excluded. Based on the genotyped SNPs that passed the above-described quality control, a total of 7,962,325 SNPs were imputed.

PLCO

Case-control ascertainment: The summary statistics from 151 HCC cases and 56,413 controls from The Prostate, Lung, Colorectal and Ovarian (PLCO) Cancer Screening Trial[11] were downloaded from https://exploregwas.cancer.gov/plco-atlas/#/downloads

Ethics: The PLCO Cancer Screening Trial is a randomized, controlled trial to determine whether certain screening exams reduce mortality from prostate, lung, colorectal and ovarian cancer. Approximately 155,000 participants were enrolled between November 1993 and July 2001. PLCO has the following five ClinicalTrials.gov registration numbers: NCT00002540 (Prostate), NCT01696968 (Lung), NCT01696981 (Colorectal), NCT01696994 (Ovarian), and NCT00339495 (EEMS). All participants provided written informed consent.

Genotyping and imputation: Genotyping was by Illumina arrays: Global Screening Array (GSA) OncoArray, Omni2.5 M, or OmniExpress (OmniX). Quality control included filtering for high missingness of phenotypes, low genotype call rate, and checks for sex mismatches, contamination, assay concordance, duplicates, relatedness, and abnormal heterozygosity[11]. The TopMed reference panel was used for genotype imputation.

Biobank Japan

Case-control ascertainment: The summary statistics for 2,122 cases and 159,201 controls from Biobank Japan[12] were downloaded from https://pheweb.jp/pheno/HepC. Cases were defined using ICD-10 code C22.0 or phecode 155.1, as described in Sakaue et al.[13]

Ethics: Biobank Japan received ethical approval from Research ethics committees at the Institute of Medical Science, the University of Tokyo, the RIKEN Yokohama Institute, and the 12 cooperating hospitals. All participants provided written informed consent.

Genotyping and imputation: Genotyping was done using the Illumina HumanOmniExpressExome BeadChip or a combination of the Illumina HumanOmniExpress and HumanExome BeadChips. Quality control (QC) of samples included exclusion of those with call rate < 0.98 and outliers from East Asian clusters identified by principal component analysis. For QC of genotypes, variants meeting any of the following criteria were excluded: (i) call rate < 99%, (ii) P value for HWE < 1.0×10^{-6}, and (iii) number of heterozygotes less than five. Using 939 samples whose genotypes were also analyzed by whole genome sequencing (WGS), additional QC was done based on the concordance rate between genotyping array and WGS. Variants with a concordance rate < 99.5% or a non-reference discordance rate ≥ 0.5% were excluded. Imputation was by SHAPEIT (v2.778) and minimac3 (v2.0.1) and used data from 1000 Genomes Project Phase 3 (version 5) as a reference.

China Kadoorie Biobank

Case-control ascertainment: The summary statistics from 670 cases with HCC and 76,022 controls from China Kadoorie Biobank[14] were downloaded from https://pheweb.ckbiobank.org/pheno/c22. Cases were defined by ICD10 code C22.

Ethics: All participants provided written informed consent. Ethical approval was obtained from the Oxford Tropical Research Ethics Committee, the Ethical Review Committees of the Chinese Center for Disease Control and Prevention, Chinese Academy of Medical Sciences, and the Institutional Review Board (IRB) at Peking University. The Chinese Ministry of Health approved the study at the start in 2004 (including export of plasma samples to Oxford) and approved electronic linkage to health insurance records in 2011. Raw genotyping data were exported from China to the Oxford CKB International Coordinating Center under Data Export Approvals 2014-13 and 2015-39 from the Office of Chinese Human Genetic Resource Administration.

Genotyping and imputation: Genotyping was done by a custom-designed Affymetrix Axiom array. Genotyping and QC followed Affymetrix best practice workflow. Imputation was performed based on EAS population data in the 1000 Genomes Phase 3 reference.

Supplementary tables

Table S1. Cohorts, phenotyping, genotyping, statistical analysis, and references.

Cohort	N cases	N controls	HCC definition	Genotyping platform	Statistical analysis			Ref.
					Software	Model	Adjustments	
CHB-CID/DBDS	917	355919	ICD10: C22.0 or C22.9	Illumina Human Global Screening Array and Illumina Human Omni Screening Array	Regenie v2.2.4	Logistic	Year of birth, sex and first 10 PCs	1
deCODE genetics	413	375109	ICD10: C22.0	Illumina HumanHap300, HumanCNV370, HumanHap610, HumanHap1M, HumanHap660, Omni-1, Omni 2.5 or Omni Express bead chips	deCODE's	Logistic	Sex, year of birth, country, lifespan, first 10 PCs	3
UK Biobank	519	417528	ICD10: C22.0	UK BiLEVE Axiom Array/ UK Biobank Axiom Array	Regenie v2.2.4	Logistic	Year of birth, sex and first 10 PCs	7
Finngen R10	500	314193	ICD10: C22.0	Illumina chip arrays<br>Affymetrix chip arrays	SAIGE 0.36.3.2	Logistic	Age, sex, genotype batch and first 10 PCs	15
AllofUS (European)	256	120594	ICD10: C22.0, C22.8, C22.9	Illumina Whole Genome Sequencing	Hail	Logistic	Age, sex, first 10 PCs	9
Trépo et al	775	1332	Imaging or histology	Illumina Human Global Screening Array	Plink v. 1.9	Logistic	First 10 PCs	10
Estonian Biobank	136	193680	ICD10: C22.0 or C22.9	Illumina Human Global Screening Array	Regenie v3.0.3	Logistic	Age at enrollment, sex, 10 PCs	8
Intermountain	81	77000	ICD10: C22.0	Illumina Global Screening array family	deCODE's	Logistic	Year of birth, sex and first 10 PCs	3
PLCO	151	56413	ICD10: C22.0	OncoArray, Omni2.5 M, OmniExpress, and Illumina Global Screening Array	SAIGE 0.43.2	Logistic	Age, sex, study center, and top 20 PCs	11
BioBank Japan	2122	159201	ICD10: C22.0<br>phecode: 155.1	Illumina HumanOmniExpressExome v.1.0/v.1.2, HumanOmniExpress v.1.0 and Human Exome BeadChip v.1.0/v.1.1	Plink v. 2.0.0	Logistic	Age, sex and first 20 PCs	12
Kadoorie Biobank	670	76022	ICD10: C22	Custom-designed Affymetrix Axiom	SAIGE 0.42.1	Logistic	Array, sex, age, age^2, region, first 11 PCs	14

Abbreviations: HCC; Hepatocellular carcinoma, Ref; reference, CHB-CC/DBDS; Copenhagen Hospital Biobank Cancer Cohort and Danish Blood Donor Study, PLCO; The Prostate, Lung, Colorectal and Ovarian (PLCO) Cancer Screening Trial.

Table S2. Baseline characteristics of hepatocellular carcinoma cases in the included cohorts.

Cohort	N cases	Male (%)	Age (y)	Obese (%)	Smoking (%)	Alcohol drinker (%)	Chronic viral hepatitis (%)
CHB-CID/DBDS	917	78	73	-	-	-	-
deCODE genetics	413	69	-	47	79	-	5
UK Biobank	519	76	70	49	73	97	7.5
Finngen R10	500	88	73	-	-	-	~1
AllofUS (European)	256	57	64	26	29	22	30
Trépo et al	775	90	65	-	-	100	0
Estonian Biobank	136	50	-	-	-	-	17
Intermountain	81	-	-	-	-	-	-
PLCO	151	89	-	-	-	-	-
BioBank Japan	2122	76	68	23	M: 82, F: 24	M: 79, F: 34	HBV: M: 12, F: 6<br>HCV: M: 44, F: 53
Kadoorie Biobank	670	63	~60	4	52	31	22 (HBsAg+)

Grey cells indicate that the information was not available. Smoking and alcohol was defined as current smoking or current alcohol consumer. Obese was defined as participants with a body mass index of more than 30 kg/m^2. Chronic viral hepatitis was defined by ICD-code except in BioBank Japan and Kadoorie Biobank, where serology was used. Baseline information of HCC cases in BioBank Japan and Kadoorie Biobank was extracted from published studies[16,17].

Table S3. Frequencies of the hepatocellular carcinoma variants in individuals of European and East Asian ancestry.

CHR	Position	Gene	Location	rsnumber	EA	NEA	EAF_EUR	EAF_EA	EAF_EUR / EAF_EA
**European ancestry GWAS meta-analysis**									
1	220973563	*MTARC1*	Intronic	rs2642442	T	C	0.6974	0.8083	0.86
3	126095012	*KLF15*	Intergenic	rs7628416	C	G	0.2413	0.6232	0.39
4	88221345	*HSD17B13*	Intergenic	rs4089	C	G	0.7252	0.6516	1.11
5	1279790	*TERT*	Intronic	rs10069690	T	C	0.2704	0.1553	1.74
6	26072992	*HFE*	Intergenic	rs144861591	T	C	0.0588	0.0006	98.0
19	19379549	*TM6SF2*	Exonic	rs58542926	T	C	0.0753	0.0697	1.08
19	45411941	*APOE*	Exonic	rs429358	T	C	0.8361	0.9115	0.91
22	44324730	*PNPLA3*	Exonic	rs738408	T	C	0.2239	0.3812	0.59
**East Asian ancestry GWAS meta-analysis**									
6	33035974	*HLA-DPA1*	UTR3	rs3179778	A	G	0.1626	0.6088	0.27
19	39737866	*IFNL4*	Exonic	rs12971396	C	G	0.1993	0.0909	2.19

Abbreviations: CHR, chromosome; EA, effect allele; NEA, non-effect allele; EAF_EUR, effect allele frequency in Europeans; EAF_EA, effect allele frequency in East Asians; EAF_EUR / EAF_EA, the ratio between EAF_EUR and EAF_EA.

Table S4. Look-up of genetic variants previously implicated in hepatocellular carcinoma and not identified in the present GWAS.

CHR	Position	Rs-number	Effect allele	Non-effect allele	Effect allele frequency	Gene	Beta	Standard error	P-value
1	228192753	rs708113	A	T	0.65	*WNT3A-WNT9A*	0.01	0.02	0.61
2	27730940	rs1260326	T	C	0.5	*GCKR*	0.04	0.022	0.07
3	39600177	rs9842969	T	C	0.09	*MOBP*	-0.03	0.045	0.53
4	103188709	rs13107325	T	C	0.06	*SLC39A8*	-0.1	0.059	0.10
5	1280028	rs2242652	A	G	0.21	*TERT*	-0.19	0.03	**2.1×10^{-9}**
6	32659878	rs9275224	A	G	0.37	*HLA-DQB1*	0.08	0.03	**6.3×10^{-3}**
8	9183596	rs4841132	A	G	0.09	*PPP1R3B*	-0.1	0.056	0.07
14	94844947	rs28929474	T	C	0.02	*SERPINA1*	0.45	0.089	**5.5×10^{-7}**
19	54676763	rs641738	T	C	0.42	*MBOAT7*	0.11	0.028	**1.5×10^{-4}**

Abbreviations: CHR, chromosome.

Table S5. Replication of HCC loci in the Million Veteran Program Cohort and the Taiwan Precision Medicine Initiative cohort.

						GWAS meta-analysis (EUR) 3,748 cases 1,861,536 controls			Million Veteran Program (EUR) 2,852 cases 447,587 controls				
**CHR**	**Position**	**Gene**	**rsnumber**	**EA**	**NEA**	**Beta**	**SE**	**P-val**	**Beta**	**95% CI lower**	**95% CI upper**	**P-val**	**Directional concordance?**
1	220973563	*MTARC1*	rs2642442	T	C	0.1735	0.0295	3.98E-09	0.063	0.006	0.119	0.03	yes
3	126095012	*KLF15*	rs7628416	C	G	0.1701	0.0302	1.77E-08	-0.014	-0.081	0.0526	0.68	no
4	88221345	*HSD17B13*	rs4089	C	G	0.2121	0.0286	1.29E-13	0.191	0.130	0.252	7.4E-10	yes
5	1279790	*TERT*	rs10069690	T	C	-0.2085	0.029	6.88E-13	-0.113	-0.176	-0.053	2.6E-4	yes
6	26072992	*HFE*	rs144861591	T	C	0.4533	0.0494	4.37E-20	0.218	0.121	0.316	1.2E-5	yes
19	19379549	*TM6SF2*	rs58542926	T	C	0.6074	0.04	3.36E-52	0.389	0.305	0.474	4.9E-18	yes
19	45411941	*APOE*	rs429358	T	C	0.2691	0.0362	1.07E-13	0.203	0.122	0.284	7.5E-7	yes
22	44324730	*PNPLA3*	rs738408	T	C	0.5538	0.027	2.69E-93	0.403	0.345	0.460	5.4E-40	yes
						**GWAS meta-analysis (East Asian)** 2,792 cases 235,223 controls			**Taiwan Precision Medicine Initiative** 4,778 cases 286,102 controls				
6	33035974	*HLA-DPA1*	rs3179778	A	G	0.2084	0.0281	1.34E-13	0.17	0.121	0.219	1.2E-12	yes
19	39737866	*IFNL4*	rs12971396*	C	G	0.314	0.05	3.40E-10	0.095	0.003	0.187	0.04	yes

The summary statistics for the outcome 'Phe_155_1: Malignant neoplasm of liver, primary' in the Million Veteran Program (MVP) cohort were downloaded from https://ftp.ncbi.nlm.nih.gov/dbgap/studies/phs002453/analyses/GIA/. Associations for the same endpoint were looked up in the Taiwan Precision Medicine Initiative Cohort at https://pheweb.ibms.sinica.edu.tw/. Directional concordance refers to concordance in directions of beta values between the GWAS and the validation cohort. Abbreviations: EA, effect allele; EUR, European ancestry; NEA, non-effect allele; SE, standard error; CI, confidence interval. * A proxy variant in high linkage disequilibrium (rs1042434, r^2=0.94 with rs12971396 in East Asian populations) was used for validating this variant in the Taiwan Precision Medicine Initiative cohort.

Table S6. Human liver single-cell sequencing transcriptomics of the HCC-associated genes.

Cell type	MARC1	KLF15	HSD17B13	TERT	HFE	TM6SF2	APOE	PNPLA3	HLA-DPA1	IFNL4
Hepatocyte_1	0.08	0.18	0.47	0	0.03	0.14	5.42	0.12	0.01	NA
Hepatocyte_2	0.09	0.21	0.42	0	0.03	0.01	3.58	0.13	0.01	NA
Hepatocyte_3	0.12	0.26	0.03	0	0.09	0.01	0.57	0.17	0.06	NA
Hepatocyte_4	0.11	0.25	0.41	0	0.03	0.17	5.22	0.14	0	NA
Hepatocyte_5	0.08	0.16	0.51	0	0.03	0.09	5.51	0.15	0.06	NA
Hepatocyte_6	0.12	0.09	0.13	0	0	0.04	6.09	0.04	0.07	NA
Cholangiocytes	0.01	0.06	0.01	0	0.01	0	0.08	0.04	0.11	NA
Hepatic_Stellate_Cells	0	0.01	0	0	0.03	0	1.02	0	0.34	NA
Periportal_LSECs	0.02	0.03	0.01	0	0.01	0.01	0.54	0	0.15	NA
Central_venous_LSECs	0.02	0.01	0.01	0	0.03	0	0.55	0	0.16	NA
Portal_endothelial_Cells	0	0.03	0.03	0	0.03	0	0.61	0	0.32	NA
Plasma_Cells	0.01	0.01	0	0	0.02	0	0.77	0	0.35	NA
Erythroid_Cells	0	0.01	0.13	0	0.03	0	2.84	0	0.23	NA
alpha-beta_T_Cells	0	0.01	0.01	0	0	0	0.77	0	0.45	NA
gamma-delta_T_Cells_1	0	0.01	0.02	0	0	0	0.89	0	0.32	NA
gamma-delta_T_Cells_2	0	0	0.01	0	0	0	0.53	0	0.03	NA
NK-like_Cells	0	0.01	0.01	0	0	0	0.74	0	0.14	NA
Non-inflammatory_Macrophage	0	0.02	0	0	0.16	0	1.63	0.02	2.19	NA
Mature_B_Cells	0	0	0	0	0	0	0.73	0	0.23	NA
Inflammatory_Macrophage	0.08	0.01	0.01	0	0.02	0	1.08	0	1.78	NA

The values are mean normalized single-cell transcriptomic data from MacParland et al.[18], based on 8444 cells from five human livers. *IFNL4* is not expressed in the liver and therefore not included in the dataset. The fill colors depict hepatocytes (light brown), cholangiocytes (forest green), hepatic stellate cells (yellow), endothelial cells (light green), and immune cells (light blue). LSEC: liver sinuisodal endothelial cells.

Table S7. Associations of GWAS-identified HCC-associated variants with incident HCC in at-risk subgroups in the UK Biobank.

				BMI ≥ 30 kg/m² nTotal = 98,737 nHCC = 279		High alcohol intake nTotal = 87,991 nHCC = 173		Type 2 diabetes nTotal = 6,992 nHCC = 73		Cirrhosis nTotal = 236 nHCC = 40		Hepatitis C nTotal = 167 nHCC = 20		Chronic hepatitis B + C nTotal = 202 nHCC = 22	
Genetic variant	EA	RA	GWAS OR	HR (95% CI)	P	HR (95% CI)	P	HR (95% CI)	P	HR (95% CI)	P	HR (95% CI)	P	HR (95% CI)	P
rs2642442	T	C	1.19	1.42 (1.17-1.72)	0.0003	1.4 (1.1-1.8)	0.006	1.4 (0.98-2.1)	0.06	1.33 (0.83-2.14)	0.24	0.66 (0.37-1.34)	0.25	0.76 (0.40-1.47)	0.42
rs7628416	C	G	1.19	1.24 (1.02-1.51)	0.03	1.4 (1.1-1.8)	0.005	0.84 (0.54-1.29)	0.4	0.85 (0.46-1.56)	0.60	1.36 (0.61-3.05)	0.46	1.84 (0.91-3.73)	0.09
rs4089	C	G	1.23	1.22 (1.01-1.48)	0.04	1.14 (0.89-1.4)	0.3	2.1 (1.36-3.29)	0.0009	1.87 (1.01-3.46)	0.04	1.18 (0.57-2.44)	0.65	1.1 (0.55-2.15	0.80
rs10069690	T	C	0.81	0.7 (0.56-0.86)	0.0006	0.69 (0.52-0.9)	0.006	0.77 (0.51-1.14)	0.19	0.92 (0.54-1.57)	0.76	0.77 (0.32-1.85)	0.56	0.76 (0.34-1.69)	0.50
rs144861591	T	C	1.57	1.19 (0.89-1.6)	0.2	1.43 (1.02-2.02)	0.04	1.85 (1.17-2.92)	0.0086	1.29 (0.78-2.13)	0.32	1.96 (0.76-5.05)	0.16	2.32 (0.92-5.83)	0.07
rs58542926	T	C	1.84	2.35 (1.87-2.96)	$3.3*10^{-13}$	2.59 (1.95-3.43)	$4.4*10^{-11}$	2.58 (1.71-3.91)	$7.3*10^{-6}$	2.1 (1.07-4.12)	0.03	1.27 (0.35-4.56)	0.71	1.14 (0.32-4.07)	0.84
rs429358	T	C	1.31	1.41 (1.08-1.8)	0.01	1.61 (1.13-2.28)	0.008	1.64 (0.95-2.84)	0.078	3.14 (1.22-8.08)	0.02	1.89 (0.64-5.56)	0.25	1.70 (0.63-4.58)	0.29
rs738408	T	C	1.73	2.14 (1.8-2.54)	$4.8*10^{-18}$	1.75 (1.4-2.2)	$1.03*10^{-6}$	2.26 (1.61-3.17)	$2.2*10^{-6}$	0.91 (0.57-1.47)	0.71	0.79 (0.30-2.02)	0.62	0.66 (0.26-1.68)	0.38

Blue shading indicates directional concordance with the effect from the European ancestry GWAS meta-analysis. Green shading indicates P<0.05. Abbreviations: EA, effect allele; RA, reference allele; OR, odds ratio; HR, hazard ratio; BMI, body mass index; HCC, hepatocellular carcinoma. GWAS odds ratios were calculated by exponentiating the per-allele estimates (betas). High alcohol intake was defined as men/women drinking more than 21/14 units per week.

Table S8. Estimates from multivariable Mendelian randomization (MVMR) with liver enzymes and cirrhosis as exposures and HCC as outcome.

Exposure	Beta (SE)	P-value	Conditional F-statistic	Q-statistic
Alanine aminotransferase	0.39 (0.17)	0.024	9.7	0.002
Cirrhosis	1.12 (0.07)	2×10^{-32}	22	
Aspartate aminotransferase	0.02 (0.14)	0.904	9.8	0.001
Cirrhosis	1.17 (0.07)	3×10^{-39}	38	
Gamma glutamyltransferase	0.09 (0.09)	0.297	10	0.016
Cirrhosis	1.18 (0.06)	9×10^{-52}	109	

Shown are the output from three MVMR analyses, each including a liver enzyme (alanine aminotransferase (ALT), aspartate aminotransferase (AST), or gamma glutamyltransferase (GGT)) together with cirrhosis. Betas and SEs are the effects on hepatocellular carcinoma. P-values refer to Betas and (SE); <0.05 is considered significant. F-statistic indicates strength of the genetic instrument (<10 indicates risk of weak instrument bias). Q-statistic assesses pleiotropy in the combined exposure instrument (<0.05 is significant).

Supplementary figures

MR: Effect on risk of HCC per SD

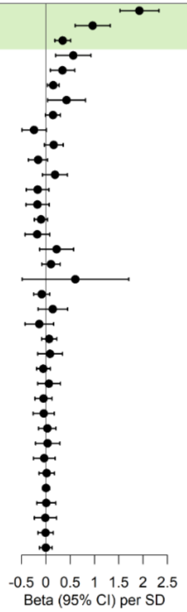

Exposure	n SNPs	IVW Beta (95% CI)	IVW P-value	Weighted median beta (95% CI)	Weighted median p-value	MR-Egger beta (95% CI)	MR-Egger p-value	Egger intercept p-value
Alanine aminotransferase	171	1.93 (1.53 - 2.33)	5.07e-21	1.37 (0.99 - 1.76)	2.06e-12	3.47 (2.76 - 4.18)	1.35e-17	1.35e-06
Aspartate aminotransferase	202	0.97 (0.61 - 1.33)	1.49e-07	0.40 (0.05 - 0.74)	0.024	2.05 (1.38 - 2.71)	7.24e-09	2.28e-04
Gamma glutamyltransferase	243	0.35 (0.19 - 0.51)	2.43e-05	0.29 (0.00 - 0.58)	0.049	0.16 (-0.10 - 0.42)	0.23	0.074
Haemoglobin concentration	239	0.57 (0.20 - 0.93)	2.28e-03	0.35 (-0.02 - 0.72)	0.063	0.76 (0.06 - 1.46)	0.034	0.52
Albumin	183	0.34 (0.09 - 0.59)	7.26e-03	0.07 (-0.27 - 0.41)	0.69	0.22 (-0.25 - 0.69)	0.36	0.54
Mean corpuscular volume	311	0.15 (0.03 - 0.28)	0.012	0.02 (-0.17 - 0.20)	0.87	0.20 (0.00 - 0.40)	0.049	0.56
Haematocrit percentage	222	0.43 (0.04 - 0.82)	0.032	0.15 (-0.21 - 0.52)	0.40	0.51 (-0.26 - 1.28)	0.20	0.80
Reticulocyte count	237	0.15 (-0.00 - 0.30)	0.056	0.04 (-0.20 - 0.28)	0.73	0.30 (0.04 - 0.57)	0.024	0.16
Cholesterol	147	-0.24 (-0.49 - 0.01)	0.063	-0.11 (-0.38 - 0.15)	0.40	-0.41 (-0.78 - -0.05)	0.027	0.19
C-reactive protein	173	0.16 (-0.03 - 0.36)	0.093	0.07 (-0.22 - 0.36)	0.64	0.20 (-0.07 - 0.47)	0.15	0.71
Apolipoprotein B	138	-0.16 (-0.36 - 0.04)	0.12	-0.06 (-0.22 - 0.09)	0.42	-0.26 (-0.49 - -0.02)	0.038	0.16
Calcium	172	0.19 (-0.07 - 0.45)	0.15	0.15 (-0.20 - 0.51)	0.40	0.31 (-0.15 - 0.78)	0.19	0.54
Triglycerides	198	-0.17 (-0.40 - 0.06)	0.16	-0.12 (-0.36 - 0.11)	0.31	-0.57 (-0.89 - -0.25)	6.91e-04	7.98e-04
LDL direct	141	-0.17 (-0.40 - 0.07)	0.16	-0.08 (-0.29 - 0.14)	0.49	-0.36 (-0.66 - -0.05)	0.024	0.062
Platelet count	362	-0.10 (-0.23 - 0.04)	0.17	-0.09 (-0.28 - 0.10)	0.37	-0.17 (-0.42 - 0.08)	0.18	0.48
Creatinine	274	-0.17 (-0.43 - 0.08)	0.18	-0.30 (-0.60 - -0.00)	0.048	-0.35 (-0.94 - 0.24)	0.25	0.52
Mean corpuscular haemoglobin concentration	84	0.22 (-0.12 - 0.57)	0.21	-0.04 (-0.44 - 0.36)	0.84	0.90 (0.26 - 1.55)	7.35e-03	0.017
Neutrophill count	250	0.11 (-0.08 - 0.29)	0.26	0.07 (-0.22 - 0.35)	0.64	0.14 (-0.25 - 0.53)	0.48	0.85
Testosterone	82	0.61 (-0.49 - 1.71)	0.28	0.38 (-0.33 - 1.10)	0.29	0.42 (-1.43 - 2.26)	0.66	0.80
IGF-1	295	-0.08 (-0.25 - 0.08)	0.33	-0.06 (-0.31 - 0.18)	0.61	-0.31 (-0.62 - 0.01)	0.056	0.100
SHBG	218	0.15 (-0.16 - 0.45)	0.34	0.14 (-0.15 - 0.43)	0.35	0.47 (-0.03 - 0.98)	0.067	0.11
Urea	134	-0.13 (-0.43 - 0.16)	0.38	-0.10 (-0.48 - 0.28)	0.60	-0.22 (-0.88 - 0.44)	0.52	0.78
Lymphocyte count	280	0.07 (-0.09 - 0.23)	0.39	0.00 (-0.26 - 0.26)	1.0	-0.02 (-0.39 - 0.35)	0.92	0.60
Urate	213	0.09 (-0.16 - 0.34)	0.49	0.17 (-0.03 - 0.36)	0.099	0.07 (-0.27 - 0.41)	0.68	0.87
Glycated haemoglobin	265	-0.05 (-0.19 - 0.09)	0.51	-0.19 (-0.44 - 0.07)	0.15	-0.35 (-0.57 - -0.12)	2.89e-03	1.06e-03
Direct bilirubin	66	0.07 (-0.17 - 0.30)	0.58	-0.01 (-0.11 - 0.08)	0.76	-0.03 (-0.28 - 0.22)	0.82	0.085
Cystatin C	261	-0.05 (-0.22 - 0.13)	0.60	0.08 (-0.10 - 0.27)	0.38	0.01 (-0.23 - 0.25)	0.91	0.47
Total protein	205	-0.04 (-0.26 - 0.17)	0.70	-0.25 (-0.55 - 0.05)	0.11	0.15 (-0.28 - 0.58)	0.49	0.31
Total bilirubin	120	0.03 (-0.15 - 0.21)	0.72	-0.01 (-0.09 - 0.07)	0.77	-0.03 (-0.22 - 0.16)	0.73	0.074
Glucose	93	0.04 (-0.21 - 0.29)	0.76	0.18 (-0.20 - 0.55)	0.36	0.06 (-0.36 - 0.48)	0.78	0.90
Apolipoprotein A	209	-0.03 (-0.25 - 0.19)	0.78	0.15 (-0.09 - 0.39)	0.23	0.33 (-0.00 - 0.66)	0.052	4.70e-03
Alkaline phosphatase	269	0.02 (-0.14 - 0.18)	0.81	-0.11 (-0.34 - 0.12)	0.35	-0.04 (-0.27 - 0.20)	0.77	0.53
Lipoprotein A	21	0.00 (-0.07 - 0.08)	0.91	-0.00 (-0.08 - 0.07)	0.92	0.05 (-0.03 - 0.14)	0.23	0.069
Red blood cell (erythrocyte) count	297	0.01 (-0.19 - 0.20)	0.93	0.03 (-0.28 - 0.34)	0.85	-0.07 (-0.43 - 0.29)	0.71	0.62
Phosphate	124	-0.01 (-0.23 - 0.22)	0.95	-0.07 (-0.42 - 0.27)	0.68	-0.01 (-0.39 - 0.37)	0.94	0.97
HDL cholesterol	231	-0.00 (-0.16 - 0.15)	0.97	0.14 (-0.08 - 0.36)	0.20	0.15 (-0.08 - 0.38)	0.20	0.070
Monocyte count	278	0.00 (-0.13 - 0.13)	0.99	0.04 (-0.19 - 0.26)	0.74	-0.09 (-0.30 - 0.12)	0.40	0.28

Fig. S1. Mendelian randomization analyses of the causal effect of 37 biomarkers on hepatocellular carcinoma. Genetic instruments for the biomarkers were derived from UK Biobank (UKB). Associations with hepatocellular carcinoma (HCC) were from the present study, with UKB excluded. Effect estimates are from the inverse variance weighted (IVW) method. The green bar indicates biomarkers that passed the Bonferroni-adjusted threshold for statistical significance ($P < 1.3 \times 10^{-3}$). SHBG: sex hormone binding globulin.

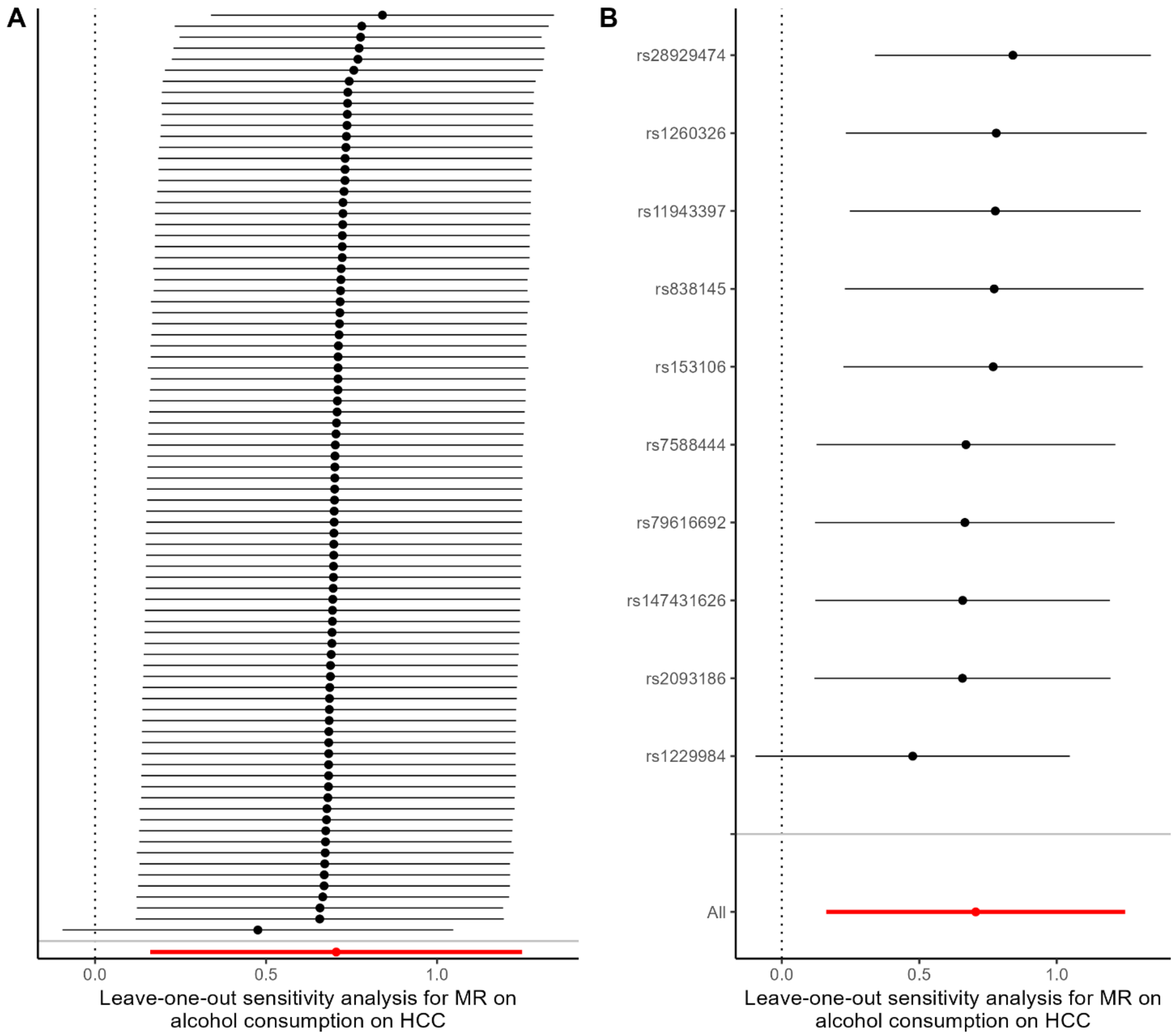

Fig. S2. Leave-one-out Mendelian randomization sensitivity analyses of alcohol consumption on hepatocellular carcinoma. The estimates are the causal estimates after leaving out one genetic variant at a time. A, shown is the leave-one-out estimates for each genetic variant included in the alcohol instrument. B, shown are the ten variants for which leaving them out of the inverse variance weighted has the largest impact.

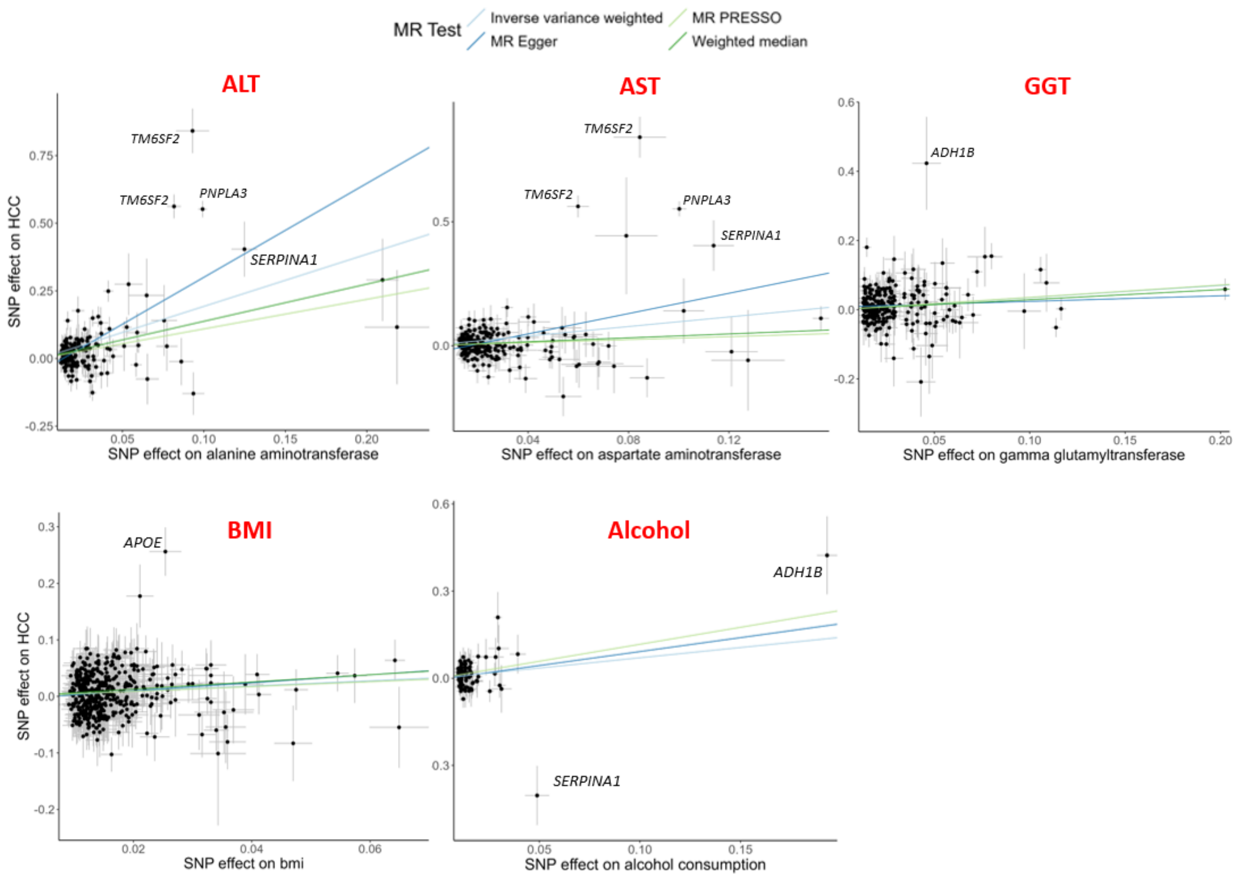

Fig. S3. Effect plots of variants included in Mendelian randomization analyses of ALT, AST, GGT, BMI, and alcohol consumption on HCC. The estimates are the estimates for the individual variants and whiskers are 95% Cis.

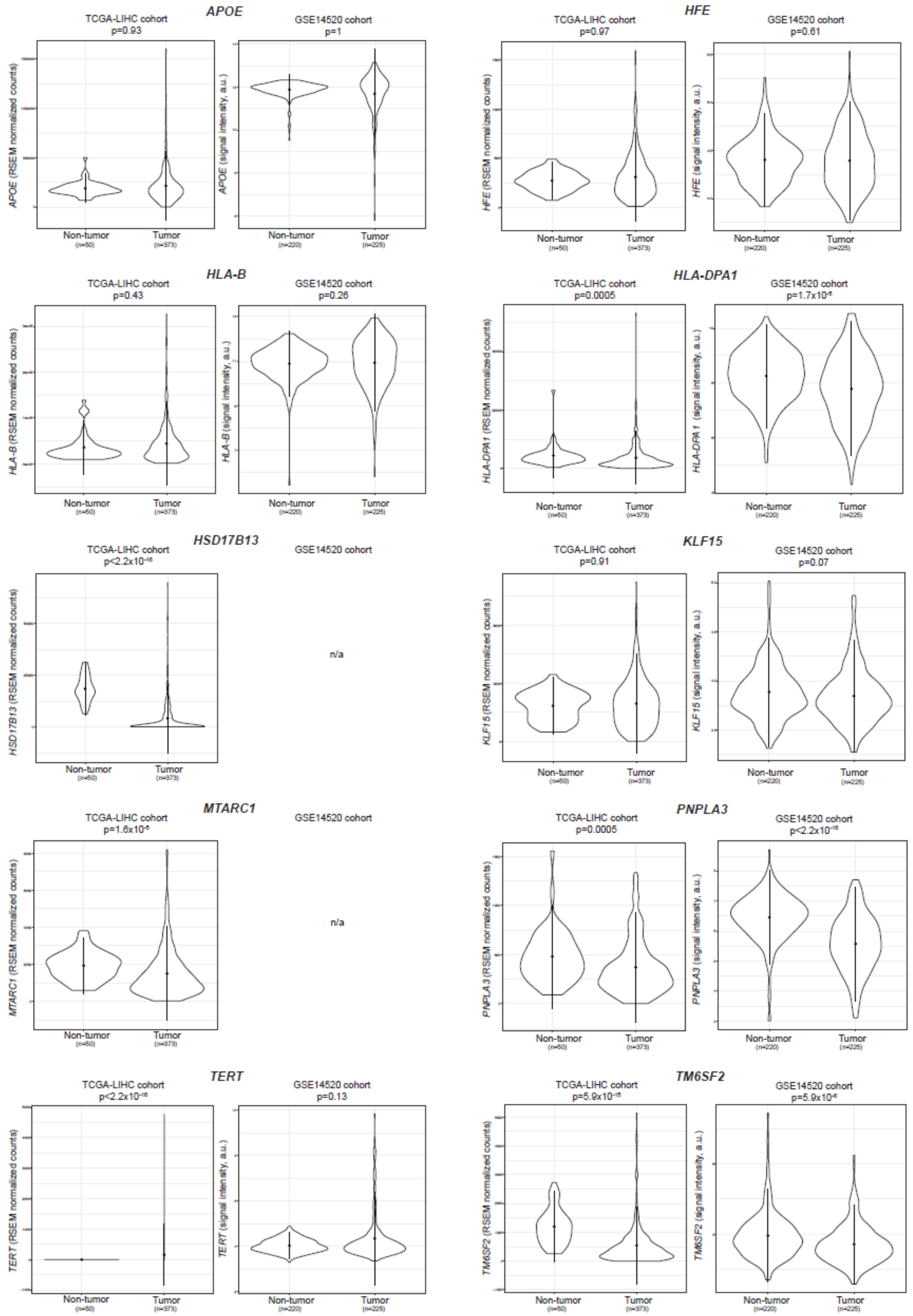

Fig. S4. Differential transcription of the ten hepatocellular carcinoma-associated genes in hepatocellular carcinomas compared to normal liver tissue.

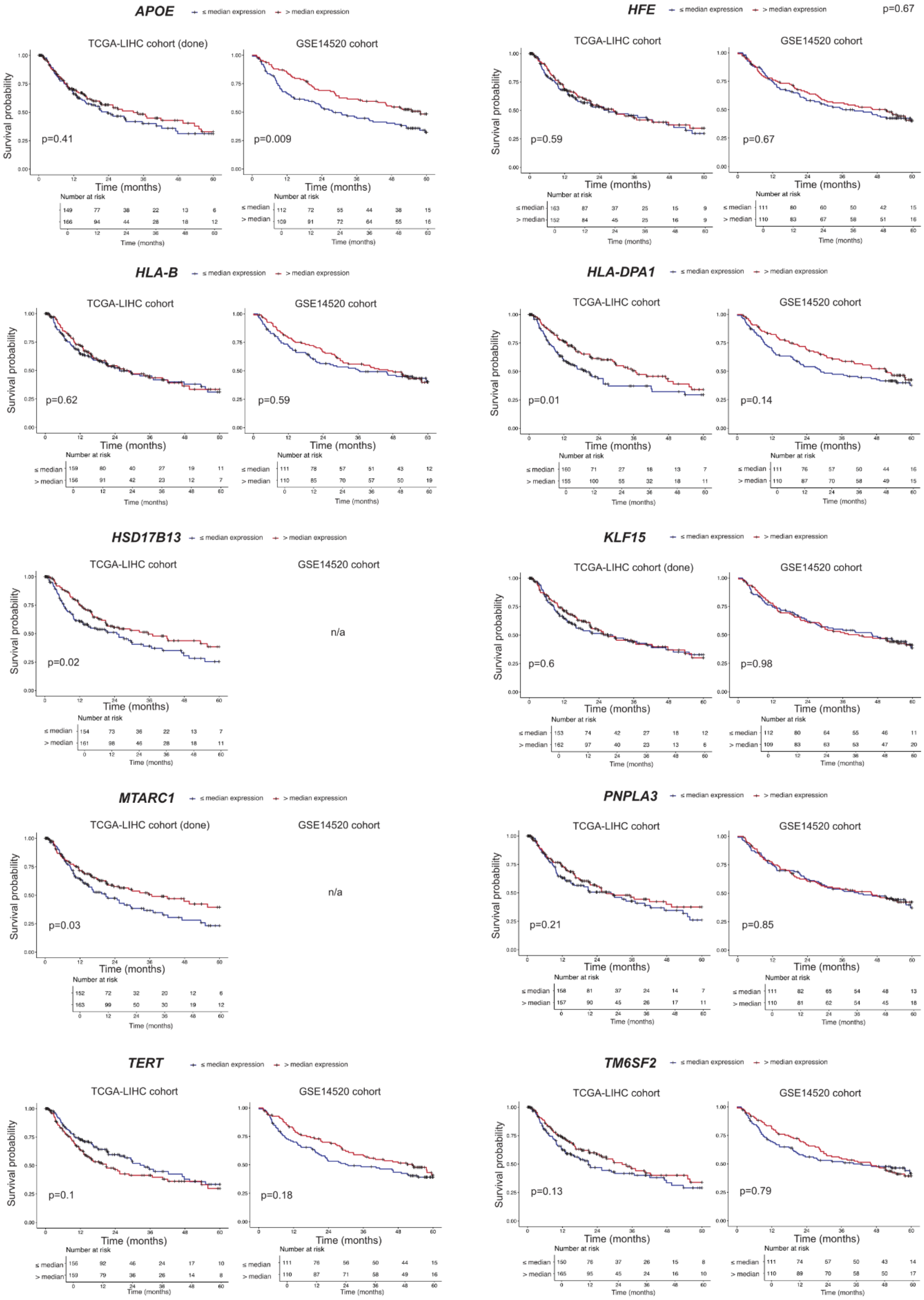

Fig. S5. Disease-free survival in hepatocellular carcinoma as a function of transcriptional level of the ten hepatocellular carcinoma-associated genes. The curves depict disease-free survival in HCC cases, stratified by RNA levels above (red) or below (blue) the median level.

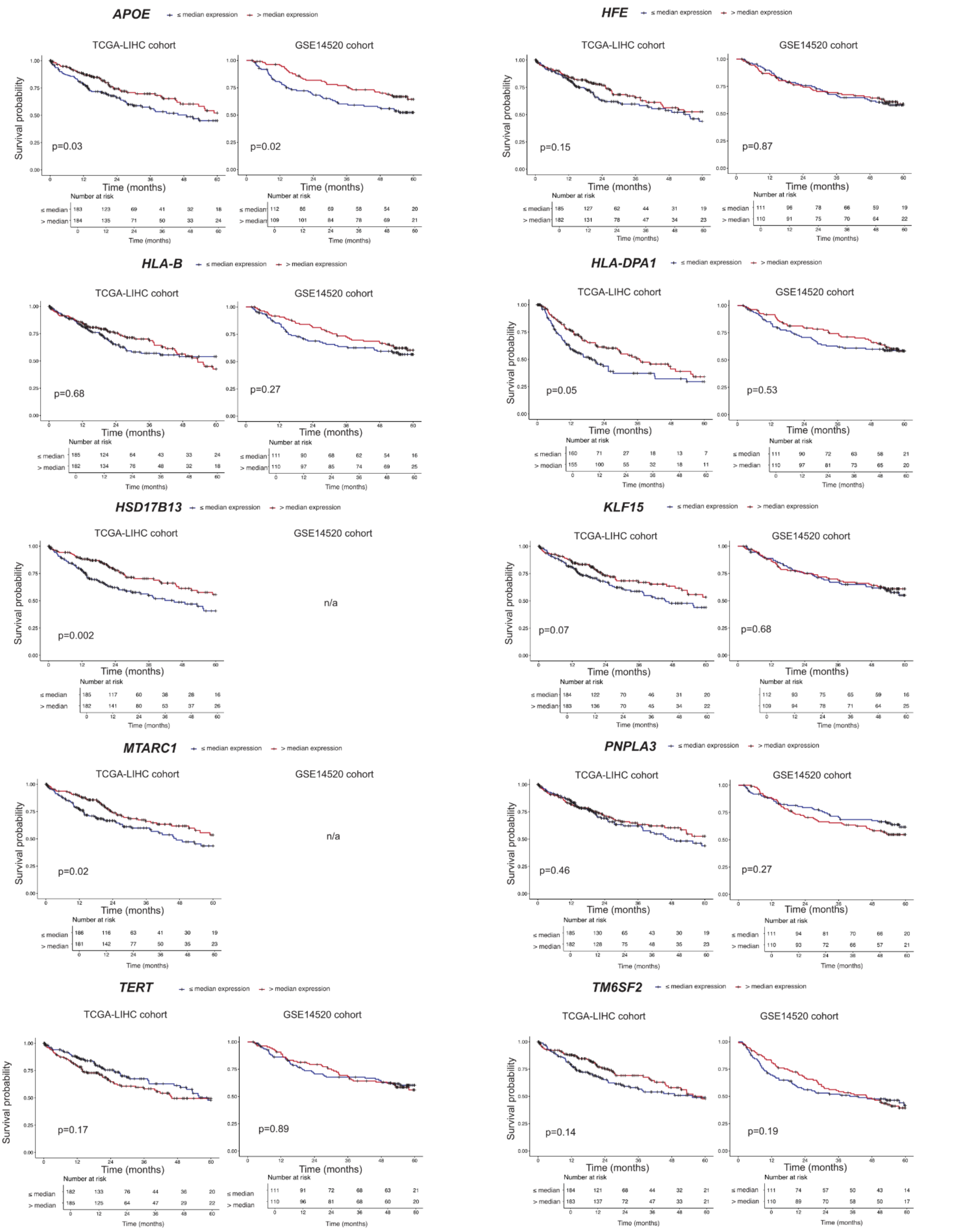

Fig. S6. Overall survival in hepatocellular carcinoma as a function of transcriptional level of the ten hepatocellular carcinoma-associated genes. The curves depict overall survival in HCC cases, stratified by RNA levels above (red) or below (blue) the median level.

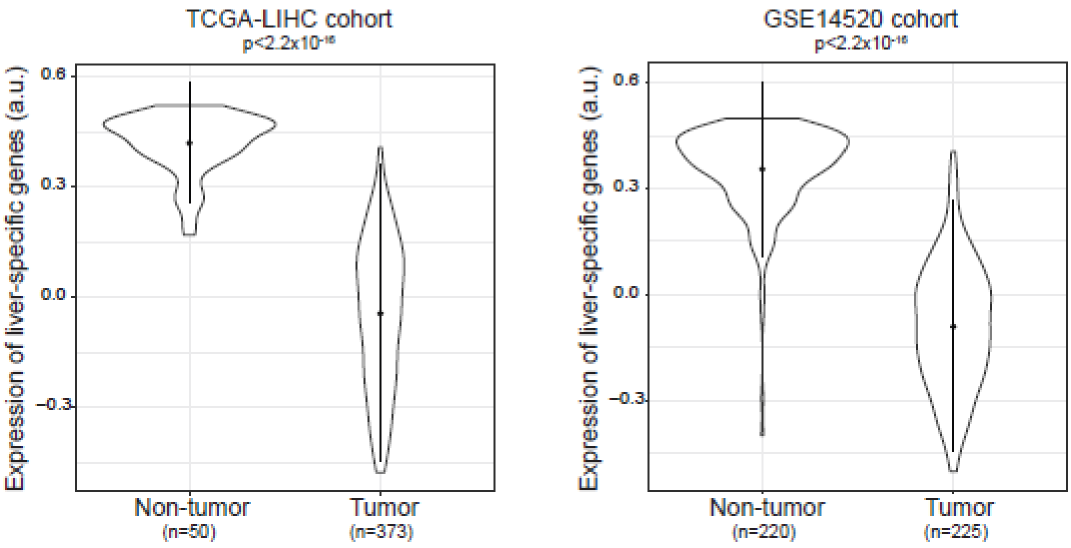

Fig. S7. Transcriptional level of 541 liver-specific genes in non-tumor and HCC. The panel of liver-specific genes was defined in Farshidfar et al[19].

Supplementary references

1. Sørensen E, Christiansen L, Wilkowski B, et al. Data Resource Profile: The Copenhagen Hospital Biobank (CHB). Int J Epidemiol 2021;50.
2. Hansen TF, Banasik K, Erikstrup C, et al. DBDS Genomic Cohort, a prospective and comprehensive resource for integrative and temporal analysis of genetic, environmental and lifestyle factors affecting health of blood donors. BMJ Open 2019;9.
3. Sveinbjornsson G, Ulfarsson MO, Thorolfsdottir RB, et al. Multiomics study of nonalcoholic fatty liver disease. Nat Genet 2022;54.
4. Sigurdardottir LG, Jonasson JG, Stefansdottir S, et al. Data quality at the Icelandic Cancer Registry: Comparability, validity, timeliness and completeness. Acta Oncol (Madr) 2012;51.
5. Kong A, Masson G, Frigge ML, et al. Detection of sharing by descent, long-range phasing and haplotype imputation. Nat Genet 2008;40.
6. Gudbjartsson DF, Helgason H, Gudjonsson SA, et al. Large-scale whole-genome sequencing of the Icelandic population. Nat Genet 2015;47.
7. Sudlow C, Gallacher J, Allen N, et al. UK Biobank: An Open Access Resource for Identifying the Causes of a Wide Range of Complex Diseases of Middle and Old Age. PLoS Med 2015;12.
8. Leitsalu L, Haller T, Esko T, et al. Cohort profile: Estonian biobank of the Estonian genome center, university of Tartu. Int J Epidemiol 2015;44.
9. Anon. Genomic data in the All of Us Research Program. Nature 2024;627.
10. Trépo E, Caruso S, Yang J, et al. Common genetic variation in alcohol-related hepatocellular carcinoma: a case-control genome-wide association study. Lancet Oncol 2022;23.
11. Machiela MJ, Huang WY, Wong W, et al. GWAS Explorer: an open-source tool to explore, visualize, and access GWAS summary statistics in the PLCO Atlas. Sci Data 2023;10.
12. Nagai A, Hirata M, Kamatani Y, et al. Overview of the BioBank Japan Project: Study design and profile. J Epidemiol 2017;27.
13. Sakaue S, Kanai M, Tanigawa Y, et al. A cross-population atlas of genetic associations for 220 human phenotypes. Nat Genet 2021;53.
14. Walters RG, Millwood IY, Lin K, et al. Genotyping and population characteristics of the China Kadoorie Biobank. Cell Genomics 2023;3.
15. Kurki MI, Karjalainen J, Palta P, et al. FinnGen provides genetic insights from a well-phenotyped isolated population. Nature 2023;613.
16. Song C, Lv J, Yu C, et al. Adherence to Healthy Lifestyle and Liver cancer in Chinese: a prospective cohort study of 0.5 million people. Br J Cancer 2022;126:815–821.
17. Ukawa S, Okada E, Nakamura K, et al. Characteristics of patients with liver cancer in the BioBank Japan project. J Epidemiol 2017;27:S43–S48.

18. MacParland SA, Liu JC, Ma X-Z, et al. Single cell RNA sequencing of human liver reveals distinct intrahepatic macrophage populations. Nat Commun 2018;9:4383.

19. Farshidfar F, Zheng S, Gingras M-C, et al. Integrative Genomic Analysis of Cholangiocarcinoma Identifies Distinct IDH-Mutant Molecular Profiles. Cell Rep 2017;18:2780–2794.

Consortia members

Danish Blood Donor Study (DBDS) genomic consortium

n	Academic degrees	First name	Surname	Email	Affiliation 1	Affiliation 2	Banner
1	PhD	Karina	Banasik	karina.banasik@cpr.ku.dk	Novo Nordisk Foundation Center for Protein Research, Faculty of Health and Medical Sciences, University of Copenhagen, Copenhagen, Denmark		DBDS GC (Banner)
2	PhD	Jakob	Bay	jabay@regionsjaelland.dk	Department of Clinical Immunology, Zealand University Hospital, Køge, Denmark		DBDS GC (Banner)
3	MSc	Jens Kjærgaard	Boldsen	jenbol@rm.dk	Department of Clinical Immunology, Aarhus University Hospital, Aarhus, Denmark		DBDS GC (Banner)
4	PhD	Thorsten	Brodersen	thobr@regionsjaelland.dk	Department of Clinical Immunology, Zealand University Hospital, Køge, Denmark		DBDS GC (Banner)
5	PhD	Søren	Brunak	soren.brunak@cpr.ku.dk	Novo Nordisk Foundation Center for Protein Research, Faculty of Health and Medical Sciences, University of Copenhagen, Copenhagen, Denmark		DBDS GC (Banner)
6	PhD	Alfonso	Buil Demur	alfonso.buil.demur@regionh.dk	Institute of Biological Psychiatry, Mental Health Centre, Sct. Hans, Copenhagen University Hospital, Roskilde, Denmark		DBDS GC (Banner)
7	**PhD**	Lea Arregui Nordahl	Christoffersen	lea.arregui.nordahl.christoffersen@regionh.dk	Department of Clinical Immunology, Zealand University Hospital, Køge, Denmark		
8	PhD	Maria	Didriksen	maria.didriksen@regionh.dk	Department of Clinical Immunology, Copenhagen University Hospital, Rigshospitalet, Copenhagen, Denmark		DBDS GC (Banner)
9	PhD	Khoa Manh	Dinh	khoadinh@rm.dk	Department of Clinical Immunology, Aarhus University Hospital, Aarhus, Denmark		DBDS GC (Banner)
10	PhD	Joseph	Dowsett	joseph.dowsett@regionh.dk	Department of Clinical Immunology, Copenhagen University Hospital, Rigshospitalet, Copenhagen, Denmark		DBDS GC (Banner)
11	PhD	Christian	Erikstrup	christian.erikstrup@skejby.rm.dk	Department of Clinical Immunology, Aarhus University Hospital, Aarhus, Denmark	Department of Clinical Medicine, Health, Aarhus University, Aarhus, Denmark	DBDS GC (Banner)
12	PhD	Bjarke	Feenstra	Bjarke Feenstra <FEE@ssi.dk>	Department of Clinical Immunology, Copenhagen University Hospital, Rigshospitalet, Copenhagen, Denmark	Department of Epidemiology Research, Statens Serum Institut, Copenhagen, Denmark	DBDS GC (Banner)
13	PhD	Frank	Geller	FGE@ssi.dk	Department of Clinical Immunology, Copenhagen University Hospital, Rigshospitalet, Copenhagen, Denmark	Department of Epidemiology Research, Statens Serum Institut, Copenhagen, Denmark	DBDS GC (Banner)
14	PhD	Daniel	Gudbjartsson	Daniel.Gudbjartsson@decode.is	deCODE Genetics, Reykjavik, Iceland		DBDS GC (Banner)
15	PhD	Thomas Folkmann	Hansen	thomas.hansen@regionh.dk	Danish Headache Center, Department of Neurology, Copenhagen University Hospital, Rigshospitalet-Glostrup, Copenhagen, Denmark		DBDS GC (Banner)
16	PhD	Dorte	Helenius Mikkelsen	dorte.helenius.mikkelsen@regionh.dk	Institute of Biological Psychiatry, Mental Health Centre, Sct. Hans, Copenhagen University Hospital, Roskilde, Denmark		DBDS GC (Banner)

17	MSc	Lotte	Hindhede	LOTHIN@rm.dk	Department of Clinical Immunology, Aarhus University Hospital, Aarhus, Denmark		DBDS GC (Banner)
18	PhD	Henrik	Hjalgrim	HHJ@cancer.dk	Danish Cancer Society Research Center, Copenhagen, Denmark	Department of Epidemiology Research, Statens Serum Institut, Copenhagen, Denmark	DBDS GC (Banner)
19	PhD	Jakob	Hjorth von Stemann	jakob.hjorth.von.stemann@regionh.dk	Department of Clinical Immunology, Copenhagen University Hospital, Rigshospitalet, Copenhagen, Denmark		DBDS GC (Banner)
20	MD	Bitten Aagaard	Jensen	biaaj@rn.dk	Department of Clinical Immunology, Aalborg University Hospital, Aalborg, Denmark		DBDS GC (Banner)
21	PhD	Andrew	Joseph Schork	andrew.joseph.schork@regionh.dk	Institute of Biological Psychiatry, Mental Health Centre, Sct. Hans, Copenhagen University Hospital, Roskilde, Denmark		DBDS GC (Banner)
22	PhD	Kathrine	Kaspersen	kathkasp@rm.dk	Department of Clinical Immunology, Aarhus University Hospital, Aarhus, Denmark		DBDS GC (Banner)
23	MSc	Bertram Dalskov	Kjerulff	berkje@rm.dk	Department of Clinical Immunology, Aarhus University Hospital, Aarhus, Denmark		DBDS GC (Banner)
24	PhD	Mette	Kongstad	mette.kongstad.01@regionh.dk	Department of Clinical Immunology, Copenhagen University Hospital, Rigshospitalet, Copenhagen, Denmark		DBDS GC (Banner)
25	PhD	Susan	Mikkelsen	susanmke@rm.dk	Department of Clinical Immunology, Aarhus University Hospital, Aarhus, Denmark		DBDS GC (Banner)
26	MD	Christina	Mikkelsen	christina.mikkelsen@regionh.dk	Department of Clinical Immunology, Copenhagen University Hospital, Rigshospitalet, Copenhagen, Denmark		DBDS GC (Banner)
27	PhD	Ioanna	Nissen	ioanna.nissen@regionh.dk	Department of Clinical Immunology, Copenhagen University Hospital, Rigshospitalet, Copenhagen, Denmark		DBDS GC (Banner)
28	PhD	Mette	Nyegaard	nyegaard@hst.aau.dk	Department of Health Science and Technology, Faculty of Medicine, Aalborg Univeristy, Aalborg, Denmark		DBDS GC (Banner)
29	DMSc	Sisse Rye	Ostrowski	sisse.rye.ostrowski@regionh.dk	Department of Clinical Immunology, Copenhagen University Hospital, Rigshospitalet, Copenhagen, Denmark	Department of Clinical Medicine, Faculty of Health and Medical Sciences, University of Copenhagen, Copenhagen, Denmark	DBDS GC (Banner)
30	PhD	Ole Birger	Pedersen	olbp@regionsjaelland.dk	Department of Clinical Immunology, Zealand University Hospital, Køge, Denmark	Department of Clinical Medicine, Faculty of Health and Medical Sciences, University of Copenhagen, Copenhagen, Denmark	DBDS GC (Banner)
31	PhD	Liam James Elgaard	Quinn	liaq@regionsjaelland.dk	Department of Clinical Immunology, Zealand University Hospital, Køge, Denmark		DBDS GC (Banner)
32	PhD	Þórunn	Rafnar	Thorunn.Rafnar@decode.is	deCODE Genetics, Reykjavik, Iceland		DBDS GC (Banner)
33	PhD	Palle Duun	Rohde	palledr@hst.aau.dk	Department of Health Science and Technology, Faculty of Medicine, Aalborg Univeristy, Aalborg, Denmark		DBDS GC (Banner)

34	PhD	Klaus	Rostgaard	klar@cancer.dk	Danish Cancer Society Research Center, Copenhagen, Denmark	Department of Epidemiology Research, Statens Serum Institut, Copenhagen, Denmark	DBDS GC (Banner)
35	PhD	Michael	Schwinn	michael.schwinn@regionh.dk	Department of Clinical Immunology, Copenhagen University Hospital, Rigshospitalet, Copenhagen, Denmark		DBDS GC (Banner)
36	PhD	Erik	Sørensen	Erik.Soerensen@regionh.dk	Department of Clinical Immunology, Copenhagen University Hospital, Rigshospitalet, Copenhagen, Denmark		DBDS GC (Banner)
37	PhD	Kari	Stefansson	kari.stefansson@decode.is	deCODE Genetics, Reykjavik, Iceland		DBDS GC (Banner)
38	PhD	Hreinn	Stefánsson	hreinn.stefansson@decode.is	deCODE Genetics, Reykjavik, Iceland		DBDS GC (Banner)
39	PhD	Lise Wegner	Thørner	Lise.Wegner.Thoerner@regionh.dk	Department of Clinical Immunology, Copenhagen University Hospital, Rigshospitalet, Copenhagen, Denmark		DBDS GC (Banner)
40	PhD	Unnur	Þorsteinsdóttir	Unnur.Thorsteinsdottir@decode.is	deCODE Genetics, Reykjavik, Iceland		DBDS GC (Banner)
41	MD	Mie	Topholm Bruun	mie.topholm.bruun@rsyd.dk	Department of Clinical Immunology, Odense University Hospital, Odense, Denmark		DBDS GC (Banner)
42	PhD	Henrik	Ullum	HEUL@ssi.dk	Statens Serum Institut, Copenhagen, Denmark		DBDS GC (Banner)
43	PhD	Thomas	Werge	thomas.werge@regionh.dk	Institute of Biological Psychiatry, Mental Health Centre, Sct. Hans, Copenhagen University Hospital, Roskilde, Denmark	Department of Clinical Medicine, Faculty of Health and Medical Sciences, University of Copenhagen, Copenhagen, Denmark	DBDS GC (Banner)
44	PhD	David	Westergaard	david.westergaard@cpr.ku.dk	Novo Nordisk Foundation Center for Protein Research, Faculty of Health and Medical Sciences, University of Copenhagen, Copenhagen, Denmark		DBDS GC (Banner)

Estonian Biobank

Name	Affiliation(s)
Lili Milani	Estonian Genome Centre, Institute of Genomics, University of Tartu, Tartu, Estonia
Andres Metspalu	Estonian Genome Centre, Institute of Genomics, University of Tartu, Tartu, Estonia
Tõnu Esko	Estonian Genome Centre, Institute of Genomics, University of Tartu, Tartu, Estonia
Mari Nelis	Estonian Genome Centre, Institute of Genomics, University of Tartu, Tartu, Estonia
Georgi Hudjashov	Estonian Genome Centre, Institute of Genomics, University of Tartu, Tartu, Estonia
