# Supplementary material for: Genome-wide meta-analysis identifies nine loci associated with higher risk of hepatocellular carcinoma development
Source: JHEP Rep. 2025 Jun 11;7(9):101485. doi: 10.1016/j.jhepr.2025.101485 (PMC12355075; doi:10.1016/j.jhepr.2025.101485)
Supplement: Multimedia component 4 [file mmc4.pdf]

JHEP|Reports

Genome-wide meta-analysis identifies nine loci associated with higher risk of hepatocellular carcinoma development

Jonas Ghouse[1,2,†], **Helene Gellert-Kristensen**[3,†], Colm J. O'Rourke[4], Anne-Sofie Seidelin[3], Gudmar Thorleifsson[5], Gardar Sveinbjörnsson[5], Vinicius Tragante[5], Chigoziri Konkwo[6,7,8], Joseph Brancale[6,7,8], Silvia Vilarinho[6,7,8], Tim M. Eyrich[3], Gustav Ahlberg[1,2], Johan S. Bundgaard[1], Søren A. Rand[1], Pia R. Lundegaard[2], Erik Sørensen[9], Christina Mikkelsen[9,10], Jacob Træholt[9], Christian Erikstrup[11], Khoa M. Dinh[11], Mie T. Bruun[12], Bitten Aa. Jensen[13], Jakob T. Bay[14], Søren Brunak[15], Karina Banasik[15,16], Henrik Ullum[17], DBDS Genomic Consortium, Estonian Biobank Research Team, Triin Laisk[18], Reedik Mägi[18], Lincoln D. Nadauld[19], Kirk U. Knowlton[19], Stacey Knight[19], Lise L. Gluud[20,21], Kirsten Vistisen[22], Einar S. Björnsson[23,24], Magnus O. Ulfarsson[5,25], Patrick Sulem[5], Hilma Holm[5], Ole B. Pedersen[14,21], Sisse R. Ostrowski[7,21], Daniel F. Gudbjartsson[5,26], Thorunn Rafnar[5], Kari Stefansson[5], Ulrik Lassen[21,27], Hans-Christian Pommergaard[21,28], Jens G. Hillingsø[21,28], Jesper B. Andersen[4], Henning Bundgaard[1,21], Stefan Stender[3,21,*]

JHEP Reports **2025**. vol. 7 | 1–10

Check for updates

Background & Aims: The genetic underpinnings of hepatocellular carcinoma (HCC) remain largely unknown. Thus, we aimed to identify new genetic risk loci for HCC.

Methods: We performed a genome-wide association study (GWAS) meta-analysis of 11 cohorts with validation in two independent cohorts. The identified variants were tested for effects on other hepatobiliary endpoints, and on incident HCC stratified by underlying risk factors. Mendelian randomization was used to assess the causal effects of a range of traits on the risk of HCC.

Results: In meta-analyses totaling 6,540 cases and 2,096,759 controls, we identified 10 associations with HCC, of which five (in *KLF15*, *HSD17B13*, *APOE*, *HFE*, and *MTARC1*) have not previously been implicated in HCC at genome-wide statistical significance. Known associations in *PNPLA3*, *TM6SF2*, *TERT*, *IFNL4*, and *HLA-DP1* were confirmed. All associations except *KLF15* were validated in independent cohorts totaling 7,630 cases and 733,689 controls. The largest per-allele effect was seen for *TM6SF2* (beta = 0.61) followed by *PNPLA3* (0.55), *HFE* (0.45), *IFNL4* (0.31), *APOE* (0.27), *HSD17B13*, *HLA-DP1*, and *TERT* (all 0.21), and *MTARC1* (0.17). The identified variants had comparable effects on incident HCC in individuals with prevalent obesity, a high alcohol intake, diabetes, or cirrhosis. Mendelian randomization analyses confirmed the causal role of obesity in HCC. We found strong correlations between genetic effects on HCC and hepatic steatosis (r^2 = 0.75), and HCC and cirrhosis (r^2 = 0.69), whereas only three loci (*APOE*, *HFE*, and *TERT*) had concordant effects on HCC and biliary tract cancer.

Conclusions: We identified and validated nine genetic variants associated with an increased risk of HCC development.

© 2025 The Author(s). Published by Elsevier B.V. on behalf of European Association for the Study of the Liver (EASL). This is an open access article under the CC BY license (http://creativecommons.org/licenses/by/4.0/).

Introduction

Hepatocellular carcinoma (HCC) is the third most common cause of cancer-related mortality in the world.[1,2] The development of HCC occurs most often in cirrhotic livers, major causes of which include chronic viral hepatitis infection, alcohol consumption, and metabolic dysfunction-associated steatotic liver disease (MASLD).[2] The prognosis of patients with HCC depends on the size and malignancy grade of the tumor and whether it has metastasized locally in the liver or to adjacent tissues and organs, as well as the severity of underlying liver disease.[3] Overall, the 5-year survival rate in patients with HCC is <20%.[2]

In addition to environmental factors, genetics also has an important role in HCC.[4] Several rare monogenic diseases confer a substantially higher risk of HCC, including α-1 antitrypsin deficiency, hemochromatosis, and glycogen storage disease, among others.[4] Moreover, common genetic variants have been linked to HCC through genome-wide association studies (GWAS) conducted over the past decade.[5–8] The most recent and largest of these, which included 1,872 HCC cases and 2,907 controls in the discovery cohort, identified variants in five different genetic regions to be associated with HCC.[5] However, the number of common genetic variants found to be associated with the risk of HCC remains low compared with the multitude of risk variants identified for other cancer types.[9]

A better understanding of the genetic factors that affect the risk of HCC could yield new insights into the pathology of the disease. In addition, whether genetic variants, individually or combined into polygenic risk scores (PRS), can be used to predict the onset or prognosis of HCC is a topic of major clinical interest.[10–12]

* Corresponding author. Address: Department of Clinical Biochemistry, Rigshospitalet, Copenhagen University Hospital, Denmark.
E-mail address: stefan.stender@regionh.dk (S. Stender).
† Shared first author.
https://doi.org/10.1016/j.jhepr.2025.101485

In this study, we aimed to identify genetic associations with HCC through GWAS meta-analysis of 11 cohorts, which, in total, included 6,540 cases with HCC and 2.1 million controls.

Methods

Cohorts, association testing, and meta-analysis

Cases were defined using hospital or registry records (International Classification of Diseases [ICD]-9 or ICD-10). Controls were defined as individuals without a known history of HCC. A full description of the cohorts and case and control definitions is provided in the supplementary data online and Table S1. Details on genotyping methods are also provided in Table S1. Each study performed a GWAS of HCC using logistic regression with at least age (or year of birth), sex, and principal components (PCs) used as covariates. We conducted three fixed-effect inverse-variance weighted (IVW) meta-analyses using METAL. The first included individuals of European ancestry from nine studies, the second from two cohorts of East Asian ancestry, and, finally, a cross-ancestry meta-analysis. Genomic inflation factors were calculated for each cohort and the full meta-analyses. Genome-wide significance was set at $p < 5\text{x}10^{-8}$.

Validation

To validate our findings in independent cohorts, we looked up associations with HCC in the publicly available data from the Million Veteran Program (MVP) cohort[13] and the Taiwan Precision Medicine Initiative (TPMI) cohort.[14] Variants identified in the European ancestry GWAS were sought validated in European ancestry participants from MVP, and variants identified in the East Asian ancestry GWAS were sought validated in TPMI. Summary statistics for the outcome 'Phe_155_1: Malignant neoplasm of liver, primary' in MVP were from https://ftp.ncbi.nlm.nih.gov/dbgap/studies/phs002453/analyses/GIA/. Associations for the same outcome were looked up in the TPMI (https://pheweb.ibms.sinica.edu.tw/). All GWAS-identified variants except rs12971396 were available. A proxy variant in high linkage disequilibrium (rs1042434, r^2 = 0.94 with rs12971396) was used for validating rs12971396 in TPMI. Successful validation was defined as $p < 0.05$ and consistent direction of effect with that observed in the discovery GWAS meta-analysis.

Concordance between genetic effects on steatosis, cirrhosis, biliary tract cancer, and HCC

Steatotic liver disease and cirrhosis are known risk factors for HCC, while biliary tract cancer is located within or in direct connection with the liver. Intrahepatic biliary tract cancer is strongly associated with cirrhosis.[15] We investigated the concordance of variant effects between steatosis, cirrhosis, biliary tract cancer, and HCC. For steatosis, we used GWAS results on MRI-measured proton density fat fraction (PDFF) from European ancestry participants in the UK Biobank (UKB).[16] For cirrhosis, we used data from a recent cirrhosis cross-ancestry GWAS meta-analysis.[17] In each GWAS, the beta-coefficients and 95% CIs for lead single nucleotide polymorphisms (SNPs) were extracted. When the GWASs and HCC results collectively had multiple SNPs at a locus, we included the lead SNP from the HCC GWAS. If a variant was missing from one of the GWASs, we used a proxy in high linkage disequilibrium (LD; r^2 >0.8) when available. No GWAS exists for biliary tract cancer. Instead, we extracted genetic associations with biliary tract cancer in European ancestry participants from UKB, defined by ICD10 codes C22.1 (intrahepatic cholangiocarcinoma), C23 (cancer of gallbladder), C24 (cancer of the extrahepatic bile duct), or ICD9 codes 1551 (intrahepatic cholangiocarcinoma), or 1561/1562/1568/1569/1560 (cancer of gallbladder or extrahepatic bile duct). The effect of each HCC variant on risk of biliary tract cancer was attained through logistic regression adjusted for age, sex, and PCs 1–10. The effects of the genetic variants on steatosis, cirrhosis, and biliary tract cancer were then plotted against their effects on HCC. Heterogeneity of effects was assessed using Cochran's Q. To provide more detail on potential biological reasons underlying the discordant and concordant effects, we examined the cellular expression patterns of the HCC-associated genes in publicly available single cell RNA-seq data from five human livers.[18]

Genetic effects on incident HCC in at-risk subgroups

To assess the effect of the genetic variants identified in the HCC GWAS in individuals with different prevalent risk factors for cirrhosis and/or HCC, we selected individuals of white European ancestry with prevalent obesity (BMI >30 kg/m^2), high alcohol intake (>21/14 units per week in men/women), type 2 diabetes (ICD10: E11, ICD9: 250), cirrhosis (ICD10: K70.3, K4.6; ICD9: 5712, 5715), chronic HBV (CHB) or HCV (ICD10: B17.0, B17.1, B18.2; ICD9: 07021, 07022, 07023, 07031, 07032, 07033, 07041, 07042, 07044, 07051, 07052, 07054, 07070, 07071), or chronic HCV (ICD10: B17.1, B18.2; ICD9: 07041, 07044, 07051, 07054, 07070, 07071) at the time of inclusion into the UKB. In each of these subgroups, Cox regression (adjusted for sex, age, and 10 PCs) was used to test associations of the eight variants identified in the European ancestry HCC GWAS.

Mendelian randomization

We investigated the potential causal role of 37 plasma biomarkers, as well as BMI and alcohol intake, on the risk of HCC. To avoid overlapping samples, we conducted a meta-analysis on all available HCC cohorts except the UKB, because all the exposure traits were either completely or partly derived from the UKB. We evaluated instrument strength by calculating the F-statistic. To ensure comparable LD structure between exposure and outcome datasets, only exposures derived from samples of European ancestry were used. We selected independent variants with genome-wide significance ($p < 5\text{x}10^{-8}$), r^2 <0.001 and non-missing rs-identifiers to serve as instrumental variables (IVs) for our MR analyses using the clumping method internal to the TwoSampleMR software and LD estimates from the European samples from the 1000G project.[19,20] We used two different Mendelian randomization (MR) methods: the IVW model as our primary model and the weighted median model as a sensitivity analysis. MR-Egger-intercept was used to test for pleiotropy. To test whether the results were driven by individual variants, we conducted leave-one-out analyses. Only associations that passed $p < 1.3\text{x}10^{-3}$ (0.05/39 traits) in the primary analyses (IVW) and had a $p < 0.05$ in our sensitivity analysis (weighted median) were considered significant. We

performed multivariable MR (MVMR) to further investigate significantly associated biomarkers, aiming to assess whether the association might be secondary to cirrhosis. In the MVMR analysis, cirrhosis was included as an exposure alongside the biomarker, and we calculated the conditional F-statistic, Q-statistic, and IVW-estimate adjusting for sample overlap in exposures with a phenotypic correlation matrix estimated from summary statistics.[21] The MVMR IVW-estimate is the remaining effect of the biomarker on HCC when accounting for cirrhosis.

Polygenic risk scores

We generated a PRS to investigate its potential to identify individuals at higher risk of progressing from cirrhosis to HCC. We also investigated whether individuals with HCC and a high PRS had a worse prognosis compared with those with a lower PRS. We created a weighted score based on the eight variants that reached genome-wide significance in the European ancestry meta-analysis. The PRS was weighted using effect estimates derived from meta-analysis excluding the CHB cohort, in which we evaluated the PRS. We used two models to evaluate disease progression: (1) from cirrhosis to HCC; and (2) from HCC to death from HCC. For each model, we estimated 10-year risks using Fine-Gray regression, which accounts for the competing risk of death from non-liver cancer causes. Time zero corresponded to the first occurrence of the exposure, and individual follow-up time ended in case of the event of interest, death, or end of follow-up.

Gene expression analyses

We analyzed transcriptomic data from HCC and non-tumor liver tissues using publicly available data from the Cancer Genome Atlas Program Liver Hepatocellular Carcinoma (TCGA-LIHC) cohort[22] and a cohort of HCC cases from China, referred to here as GSE14520.[23] For the TCGA-LIHC cohort, we downloaded level 3 RNA-seq data from Broad GDAC Firehose (https://gdac.broadinstitute.org/). Normalized transcriptome data calculated by RNA-Seq by Expectation Maximization (RSEM) software were available for 373 tumor and 50 non-tumor tissues collected during surgical resection in the USA. Clinical data were downloaded from cBioPortal.[24] Disease-free and overall survival data were available for 313 and 365 patients, respectively. For the GSE14520 cohort, normalized expression array (Affymetrix) data were downloaded from the Gene Expression Omnibus.[23] Probes were collapsed to individual gene values based on median signal intensity, quantified in arbitrary units. Transcriptome data were available for 225 tumor and 220 paired non-tumor hepatic tissues collected during surgical resection in a predominantly Asian population. Disease-free and overall survival data were available for 221 patients. Differentially expressed genes between groups were identified using Wilcoxon rank-sum test with continuity correction. Tumors were stratified into high (>median) and low (≤median) gene expression groups. Associations with disease-free and overall survival were determined by log-rank test, with visualization as Kaplan-Meier curves ('survival' and 'survminer' packages in R).

Results

Genome-wide association results

We included nine European ancestry GWAS and two East Asian ancestry GWAS in our meta-analyses (Fig. 1). Baseline characteristics of cases from the included studies are shown in Table S2. In the European ancestry GWAS meta-analysis (n = 9

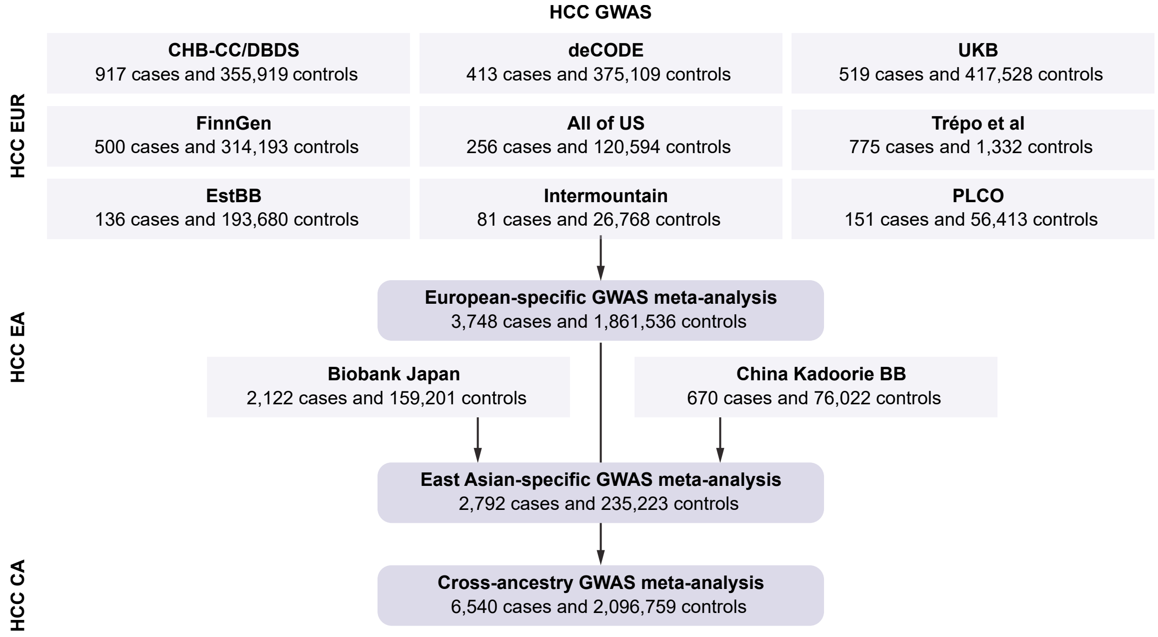

Fig. 1. Overview of the study design. CA, cross-ancestry; CHB-CC/DBDS, Copenhagen Hospital Biobank Cancer Cohort and Danish Blood Donor Study; EA, East Asian ancestry; EUR, European ancestry; HCC, hepatocellular carcinoma; UKB, UK Biobank; EstBB, Estonian Biobank; PLCO, The Prostate, Lung, Colorectal and Ovarian Cancer Screening Trial.

studies, 3,748 cases, and 1,861,536 controls), we identified eight genome-wide significant variants (Fig. 2 and Table 1), of which five (*KLF15*, *HFE*, *HSD17B13*, *APOE*, and *MTARC1*) have not previously been implicated in HCC at genome-wide statistical significance. In the East Asian ancestry meta-analysis (n = 2 studies, 2,792 cases, and 235,223 controls), we identified two variants that reached genome-wide significance (in or near *HLA-DPA1* and *IFNL4*). A cross-ancestry

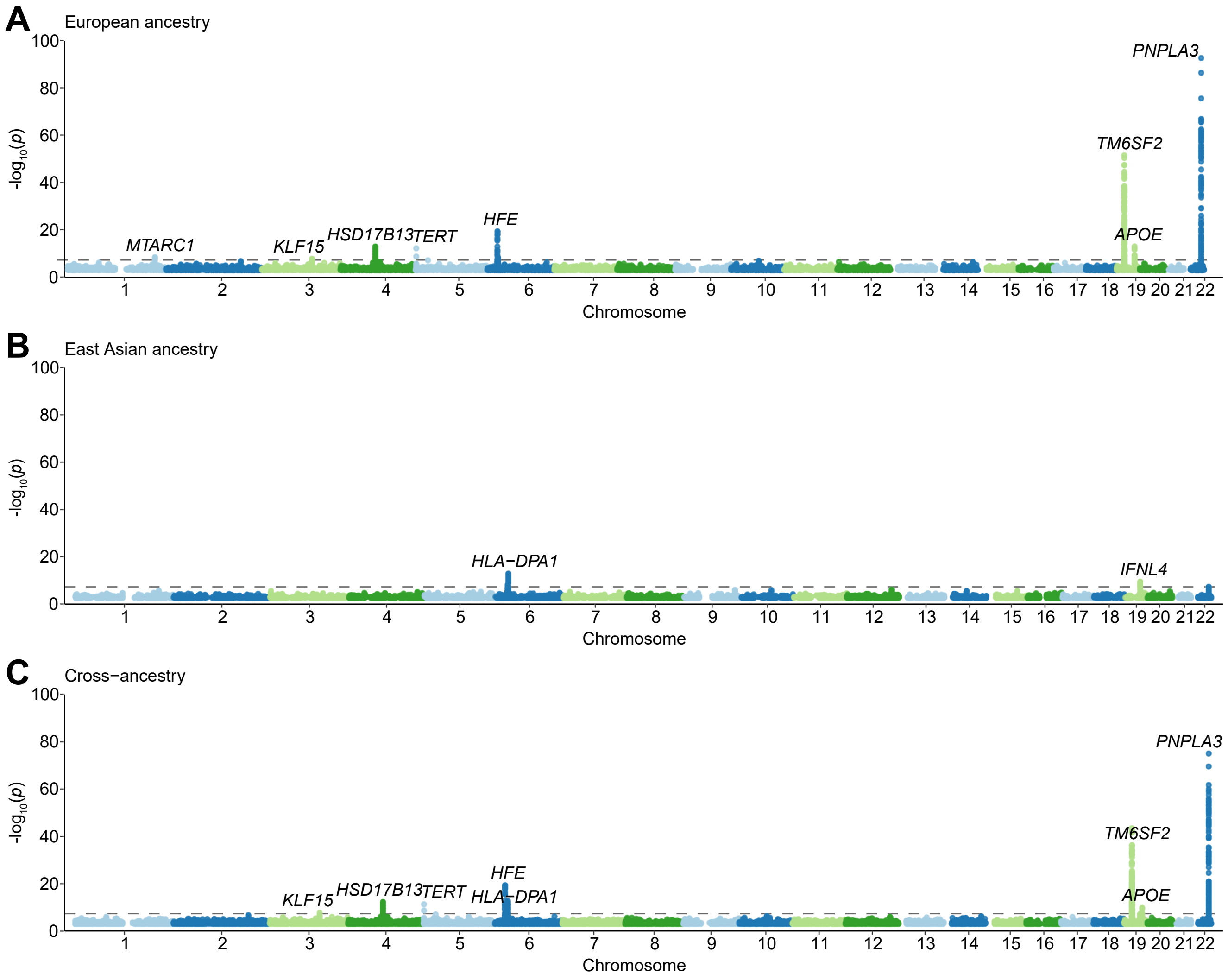

Fig. 2. Manhattan plots for HCC GWAS meta-analyses in individuals with European ancestry (A), East Asian ancestry (B), and cross-ancestry (C). The dashed horizontal lines depict the threshold for genome-wide significance, $p < 5\times10^{-8}$. GWAS, genome-wide association study; HCC, hepatocellular carcinoma.

Table 1. Risk loci for HCC identified in the GWAS meta-analyses.

Chr	Position	Gene	Location	rs number	EA	NEA	Beta	SE	*p* value
**European ancestry GWAS meta-analysis of 3,748 cases and 1,861,536 controls**									
1	220973563	*MTARC1*	Intronic	rs2642442	T	C	0.1735	0.0295	$3.98E^{-09}$
3	126095012	*KLF15*	Intergenic	rs7628416	C	G	0.1701	0.0302	$1.77E^{-08}$
4	88221345	*HSD17B13*	Intergenic	rs4089	C	G	0.2121	0.0286	$1.29E^{-13}$
5	1279790	*TERT*	Intronic	rs10069690	T	C	-0.2085	0.029	$6.88E^{-13}$
6	26072992	*HFE*	Intergenic	rs144861591	T	C	0.4533	0.0494	$4.37E^{-20}$
19	19379549	*TM6SF2*	Exonic	rs58542926	T	C	0.6074	0.04	$3.36E^{-52}$
19	45411941	*APOE*	Exonic	rs429358	T	C	0.2691	0.0362	$1.07E^{-13}$
22	44324730	*PNPLA3*	Exonic	rs738408	T	C	0.5538	0.027	$2.69E^{-93}$
**East Asian ancestry GWAS meta-analysis of 2,792 cases and 235,223 controls**									
6	33035974	*HLA-DPA1*	UTR3	rs3179778	A	G	0.2084	0.0281	$1.34E^{-13}$
19	39737866	*IFNL4*	Exonic	rs12971396	C	G	0.314	0.05	$3.40E^{-10}$

Positions are on human genome build 37 (GRCh37). Chr, chromosome; EA, effect allele; GWAS, genome-wide association study; HCC, hepatocellular carcinoma; NEA, non-effect allele.

meta-analysis that combined all studies (6,540 cases and 2,096,759 controls) did not yield any additional loci. We observed no evidence of genomic inflation in the European or East Asian ancestry meta-analyses (λ_{GC_EUR} 1.03 and λ_{GC_EA} 1.00). We found that the SNP-based heritability estimates in Europeans and Asians were 3.4% (SE: 1.1%) and 0.7% (SE: 1.0%), respectively.

Of the 10 unique variants identified in the European or East Asian specific meta-analysis, eight were mainly driven by associations in Europeans, whereas two were only observed in East Asians (Table 1). The lead variant rs144861591 at the *HFE* locus was nearly 100-fold more common in European populations compared with individuals of East Asian ancestry (Table S3). The variant rs12971396 in *IFNL4* is in LD (r^2 = 1.0 in East Asian populations) with another variant at the same locus (rs12979860) that has been strongly associated with chronic HCV infection, a main driver of both cirrhosis and HCC in East Asia.[25] *IFNL4* rs12971396 was associated with HCC in Biobank Japan (p = 2.9x10^{-12}) but not in the China Kadoorie Biobank (p = 0.88).[26] The lead variants at *PNPLA3*, *TM6SF2*, and *APOE* were either coding or in high LD with a coding variant and have all been previously implicated in the full spectrum of MASLD, including cirrhosis and HCC.[8,11] The lead *TERT*-variant rs10069690 is in LD (r^2 = 0.64 in Europeans) with rs2242652, a variant that has previously been associated with HCC.[8] The rs2242652-variant was also associated with HCC in our study (p = 2.1x10^{-9}; Table S4). We also re-evaluated eight variants that have been associated with HCC in previous GWAS or candidate gene studies and not detected in the present GWAS (Table S4). Of these eight variants, three were associated with HCC: *SERPINA1* rs28929474 (p = 5.5x10^{-7}), *MBOAT7* rs641738 (p = 1.5x10^{-4}), and *HLA-DQB1* rs9275224 (p = 6.3x10^{-3}).

Validation

A total of 10 loci were identified through HCC GWAS meta-analyses. We sought to validate the associations in independent cohorts, namely the MVP (n = 2,852 cases and 447,587 controls) for the eight loci identified in European ancestry GWAS, and the TPMI cohort (n = 4,778 cases and 286,102 controls) for the two loci identified in individuals of East Asian ancestry. Of the 10 associations in total, all except the one near *KLF15* were replicated with consistent direction of effect and p <0.05 (Table S5). Seven of the 10 associations (all but *KLF15*, *MTARC1*, and *IFNL4*) were replicated with a more stringent Bonferroni corrected threshold of p <0.005 (0.05/10 tested variants).

Comparison of genetic effects on steatosis, cirrhosis, biliary tract cancer, and HCC

Among 18 distinct variants (15 previously associated with steatosis, eight associated with HCC in European-ancestry individuals), we found a strong correlation (r^2 = 0.75, p = 2x10^{-6}) between effects on steatosis and HCC (Fig. 3A). Concordantly, using a set of 16 unique variants previously associated with cirrhosis (15 variants) and HCC (10 variants) in cross-ancestry analyses, we observed a strong correlation (r^2 = 0.69, p = 2x10^{-5}, Fig. 3B). We observed some notable differences in variant effects. For instance, five variants in or near *TM6SF2*, *PNPLA3*, *HFE*, *APOE*, and *MTARC1* had comparably larger effects on HCC than on hepatic steatosis (p <0.05 by Cochran's Q test), while HCC-associated variants in *HSD17B13, KLF15,* and *TERT* did not significantly associate with steatosis. In the comparison between cirrhosis and HCC, we found that variants in *HFE, TM6SF2,* and *TERT* displayed larger effects on HCC compared with their effects on cirrhosis, while the *KLF15* variant had no effect on cirrhosis and only affected the risk of HCC. There was no significant overlap between genetic effects on HCC and biliary tract cancer (r^2 = -0.035, p = 0.42), with three exceptions, namely *TERT*, *HFE*, and *APOE*, which all had concordant effects on both HCC and biliary tract cancer (p = 0.004, 0.008, and 0.003, respectively, for association with biliary tract cancer) (Fig. 3C). To shed more light on the discordant and concordant effects between HCC and biliary tract cancer, we analyzed single cell RNA-seq data from five human livers (Table S6). No clear differences in cellular expression patterns emerged. *APOE,* which affects both HCC and biliary tract cancer, was primarily expressed in hepatocytes. *TERT* and *HFE*, which also affect both cancers, were expressed at low levels across hepatic cell types.

Genetic effects on incident HCC in at-risk subgroups

We sought to assess whether the effect of the genetic variants on HCC differed by underlying risk factors (Table S7). Each of the eight variants identified in the European ancestry HCC GWAS was tested for association with incident HCC in UKB participants with prevalent obesity, high alcohol consumption, type 2 diabetes, cirrhosis, or chronic hepatitis. Across the 40 tests (eight variants tested in five subgroups), there was a high (35/40 or 88%) concordance of effects with those seen in the GWAS. Of the 40 tests, 21 were nominally statistically significant (p <0.05) and nine were significant using a more stringent Bonferroni-adjusted threshold of 0.001 (0.05/40 tests). The magnitude of the effect estimates tended to be larger in the at-risk subgroups compared with the GWAS. Of the 40 tested associations, 27 (68%) had numerically higher effects than the GWAS estimate for that variant. Associations in individuals with hepatitis C specifically were similar to those seen for any chronic hepatitis (Table S7).

Mendelian randomization

We used MR to evaluate the potential causal effects of a range of biochemical, metabolic, and behavioral exposures on the risk of HCC. We first tested a panel of 37 blood biomarkers (Fig. S1). Of these, increasing levels of alanine transaminase (ALT), aspartate transaminase (AST), and gamma-glutamyl transferase (GGT) were associated with HCC (p <1.3x10^{-3} [0.05/39 tests]), but ALT and AST showed evidence of pleiotropy (Egger intercept p <0.05). Cirrhosis explained most of the effects of ALT, AST, and GGT on HCC, with overall strong attenuation in effect estimates when accounting for the genetic effects of cirrhosis in MVMR analyses (Table S8).

We then tested the effect of metabolic and behavioral exposures on the risk of HCC. A higher BMI increased the risk of HCC (Fig. 4). We also found a nominally significant association between alcohol intake and risk of HCC (p = 0.03). In sensitivity analyses, the association with alcohol was found to be largely driven by *AHD1B* rs1229984, which affects alcohol intake, but with consistent direction of effects after exclusion of the

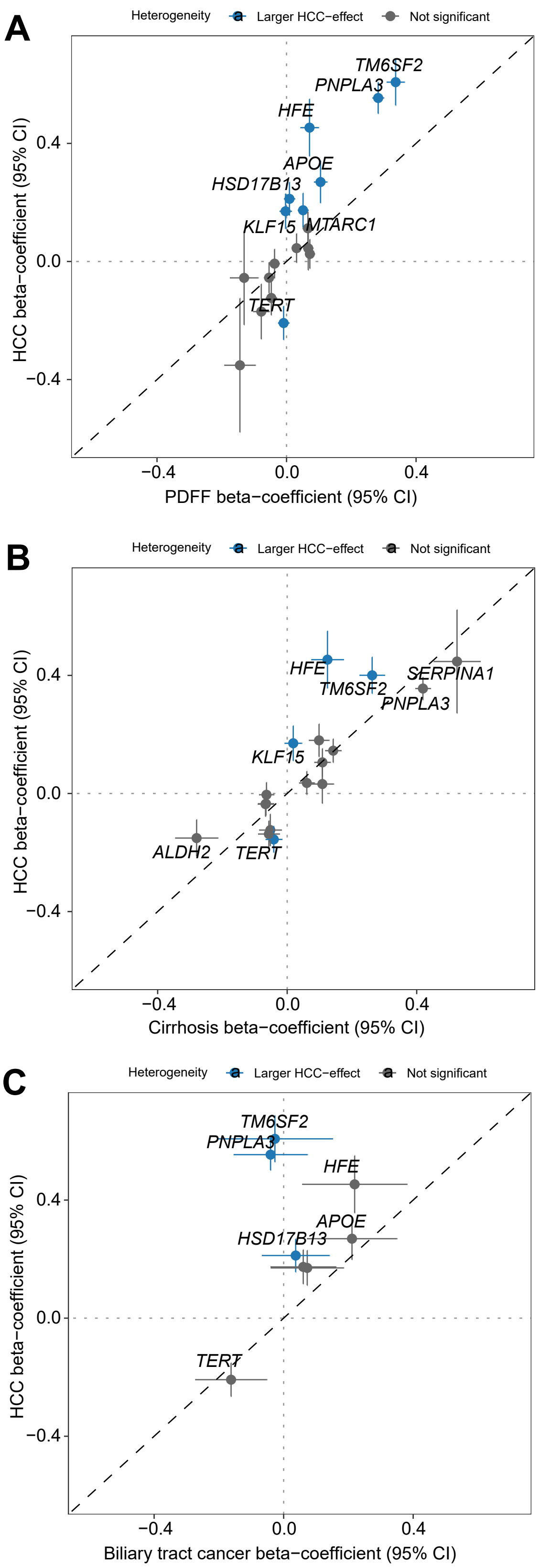

Fig. 3. Comparison of genetic associations with HCC, hepatic steatosis, cirrhosis, and biliary tract cancer. Variants that had stronger effects (p <0.05 by Cochran's Q test) on HCC compared with steatosis, cirrhosis, or biliary tract cancer, respectively, are in blue. In all analyses, HCC effects were derived from the cross-ancestry meta-analysis in the present study. (A) Effects of 15 previously reported hepatic steatosis variants and eight HCC variants identified in this study, totaling 18 distinct signals. The proton density fat fraction (PDFF) effects were derived from hepatic magnetic resonance imaging (MRI) in UKB.[16] (B) Effects of 15 previously reported cirrhosis variants and 10 HCC variants from this study, totaling 16 distinct signals. The cirrhosis effects were derived from a previous GWAS.[17] (C) Effects of eight HCC variants identified in the present study. Effects on biliary tract cancer were derived from European-ancestry UKB participants (893 cases and 458,134 controls). The dashed identity line ($y = x$) is shown for reference in each panel. GWAS, genome-wide association study; HCC, hepatocellular carcinoma; UKB, UK Biobank.

variant (Fig. S2). Variant-level effect plots for ALT, AST, GGT, BMI, and alcohol are shown in Fig. S3.

Polygenic risk scores and HCC

We evaluated whether a PRS could aid in identifying individuals with cirrhosis who were more likely to progress to HCC, as well as those with poorer survival once diagnosed with HCC. Among 4,258 individuals with cirrhosis in CHB, 315 developed HCC during follow-up. A higher PRS was associated with an increased risk of HCC after the diagnosis of cirrhosis. Individuals with cirrhosis and a high PRS (top 20%) had a 10-year HCC risk of 13.0% (95% CI 11.0–15.0) compared with 6.9% (95% CI 6.0–7.8; p for difference <0.001) for individuals in the bottom 80% of the PRS. However, we found no significant association between a higher PRS and HCC survival. Among 918 individuals with HCC, 395 died from HCC during follow-up, with individuals in the top 20% of the PRS having a similar 10-year risk (53.0%, 95% CI 45.0–61.0) to those in the bottom 80% (48.0%, 95% CI 44.0–52.0; p for difference = 0.60).

Gene expression analyses

We sought to determine whether expression of genes harboring or located near HCC-associated SNPs was affected in HCC, and whether transcriptional activity of these genes may yield prognostic value. To assess this, we conducted analyses of transcriptome data of liver tissue from two cohorts of patients with HCC, totaling 373 tumors and 50 non-tumoral tissues, and 225 tumors and 220 non-tumoral tissues, respectively.[22,23] First, we assessed differences in baseline expression levels of the nine genes with available data (all but *IFNL4*). *HSD17B13*, *TM6SF2*, *PNPLA3*, *MTARC1*, and *HLA-DP1* exhibited lower expression in HCC compared with non-tumor liver tissues (Fig. S4). Second, we tested whether the overall or disease-free survival differed by transcriptional level. The only significant association was seen for *HSD17B13,* for which RNA levels below compared with above the median were associated with lower overall survival (Bonferroni corrected p <0.005; Figs S5 and S6). For comparison, we also tested a panel of 541 genes with liver-specific expression and found that these were, on average, substantially downregulated in HCC compared with normal liver tissue (Fig. S7).

Discussion

This study identified 10 variants associated with HCC based on data from GWAS meta-analyses including more than 6,500 cases and 2 million controls. Nine of the associations were validated in independent cohorts totaling 7,630 cases

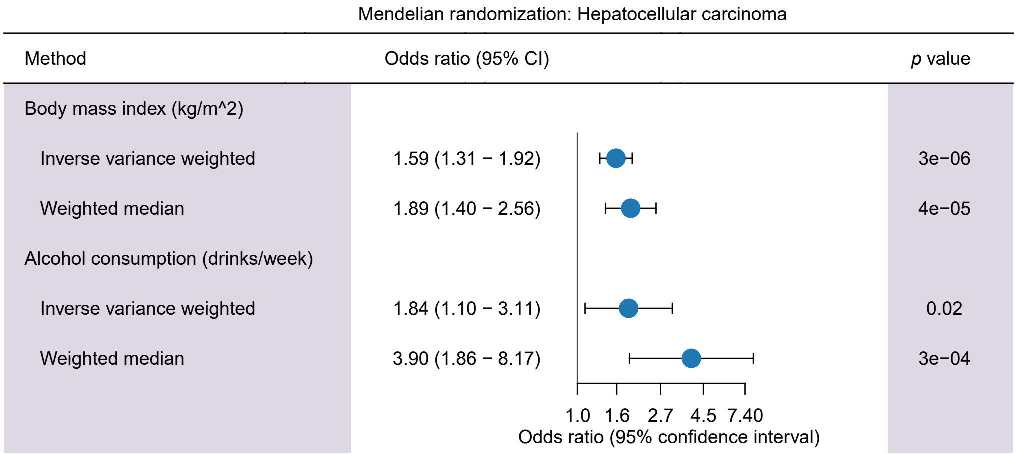

Fig. 4. Mendelian randomization analyses of the effect of BMI and alcohol intake on HCC. The HCC odds ratios are for a genetically proxied 1-SD increase in BMI (top) or alcohol consumption (bottom), respectively. HCC, hepatocellular carcinoma.

and 733,689 controls. Most of the risk variants were found to also associate with the risk of cirrhosis, a major predisposing factor for the development of HCC. The identified variants had comparable effects on incident HCC in individuals with prevalent obesity, a high alcohol intake, diabetes, or cirrhosis.

Our findings align with those from previous HCC GWASs. The most recent of these studies included 1,872 HCC cases and 2,907 controls of European descent and identified five loci in or near *PNPLA3*, *TM6SF2*, *TERT*, *HLA*, and *MOBP* to be associated with HCC.[5] Another study of 1,866 HCC cases and 197,745 controls from Biobank Japan identified an association at the *IFNL4* locus.[26] We replicated associations in *PNPLA3*, *TM6SF2*, *TERT*, *HLA*, and *IFNL4*, but could not confirm associations at the *MOBP* locus. We also report five variants near genes that have not previously reached genome-wide significance for association with HCC: *KLF15*, *HFE*, *HSD17B13*, *APOE*, and *MTARC1*. Finally, we confirm associations with HCC for the candidate genes *SERPINA1* and *MBOAT7*.

The lead variant at the *HFE* locus is in perfect LD (r^2 = 1.0) with *HFE* p.Cys282Tyr, the most common cause of hemochromatosis and a known risk factor for HCC.[27] In other words, although *HFE* has not been implicated in previous HCC GWASs, it should be viewed as a known risk locus for this cancer. The variant at *HSD17B13* affects the risk of steatohepatitis and cirrhosis and has also previously been associated with HCC, albeit at sub-genome-wide significance.[7,28] Variation at *APOE*, *MTARC1,* and *MBOAT7* has been found to confer risk of hepatic steatosis and cirrhosis.[29,30] The effects of these loci on HCC are likely secondary to their effects on cirrhosis. The *SERPINA1* variant is a known cause of α-1 antitrypsin deficiency, which confers a higher risk of cirrhosis and, in turn, HCC. The lead variant at *TERT*, rs10069690, differs from the lead variant in previous studies, rs2242652. While the two variants are in moderate linkage (r^2 = 0.62), recent functional studies indicate that they exert distinct biological effects, with rs10069690 (but not rs2242652) causing a change in *TERT* splicing.[31] The association between the *IFNL4* locus and HCC was only observed in the East Asian samples from Biobank Japan.[26] A potential reason for this is that *IFNL4* mediates its risk-increasing effect on HCC via its primary effect on increased susceptibility to chronic HCV.[25] Approximately 50% of the HCC cases in Biobank Japan were positive for HCV, whereas HBV was the predominant form of hepatitis in cases from the China Kadoorie Biobank. Intriguingly, Hassan *et al.* did not detect associations with *IFNL4* in their recent HCC GWAS of non-Hispanic white Americans, despite 39% of the HCC cases in that study having HCV.[5] Therefore, it remains possible that the *IFNL4* locus exerts effects on HCC that are distinct from its effects on HCV. Other studies have reported complex relationships between *IFNL4* and liver disease. Eslam *et al.*[32] found that *IFNL4* variants associated with higher IFN treatment response rates were also associated with increased inflammation and fibrosis regardless of liver disease etiology. In other words, *IFNL4* variants that increase HCV clearance may also increase fibrosis. Thus, the risk-increasing allele may differ depending on what outcome the study is focusing on. *KLF15* encodes Kruppel-like factor 15, a transcription factor that is highly expressed in liver tissue.[33,34] The *KLF15* association was not replicated in the MVP cohort and, therefore, should be viewed as preliminary, requiring replication in other cohorts. Taken together, the GWAS associations reported here highlight the importance of MASLD, cirrhosis, hemochromatosis, α-1 antitrypsin deficiency, and chronic viral hepatitis in the development of HCC.

The genetic effects on steatosis and cirrhosis were generally concordant with their effects on HCC, with some heterogeneity. For example, *TM6SF2* and *HFE* had larger effects on HCC than on steatosis and cirrhosis, while *TERT* and *KLF15* had no effects on steatosis and only small or no effects on cirrhosis. By contrast, there was remarkably little overlap between effects on HCC and biliary tract cancer.

The two loci with the strongest effects on HCC, *PNPLA3* and *TM6SF2*, did not show any evidence of association with biliary tract cancer. Overall, there was no correlation between genetic effects on HCC and cholangiocarcinoma. Interestingly, the variants in *HFE*, *APOE*, and *TERT* displayed concordant effects on HCC and biliary tract cancer. The lead variant at the *HFE* locus is a known cause of hemochromatosis, a disorder that has been associated with both HCC and biliary tract

cancer.[35] *APOE* encodes an apolipoprotein that binds to circulating very low-density lipoprotein (VLDL) cholesterol. *TERT* encodes telomerase, which is known to have a key role in carcinogenesis across tumor types. TERT, and telomeres, has been implicated in both HCC and biliary tract cancer in previous studies.[36–38]

Most HCC cases develop in cirrhotic livers, major causes of which include chronic viral hepatitis, a high intake of alcohol, and MASLD. We found that the risk variants had effects on incident HCC that were similar in individuals with cirrhosis, obesity, type 2 diabetes, or a high alcohol intake. The variants were not associated with HCC in chronic viral hepatitis cases, but the small sample size of this subgroup limited the statistical power. The magnitude of the effects on HCC tended to be larger in these at-risk subgroups than in the overall GWAS meta-analyses, in alignment with previous findings of gene–environment synergistic effects influencing the entire spectrum of MASLD, including HCC.[17]

MR analyses supported a causal effect of adiposity on higher risk of HCC. Although not reaching the threshold for statistical significance, the estimates for alcohol intake were also consistent with a causal, risk-increasing effect on HCC. Moreover, we found that genetically proxied higher liver enzymes were associated with a higher risk of HCC. However, when accounting for the genetic effects of liver cirrhosis, the associations of liver enzymes with HCC were substantially attenuated. These findings underline that plasma liver enzymes *per se* do not cause HCC, but that the association is driven by chronic liver disease. We did not detect evidence supporting a causal association with HCC for a range of other circulating biomarkers, including plasma lipids and lipoproteins, C-reactive protein, and glucose.

We found that individuals with cirrhosis and a PRS in the top 20% had a twofold higher risk of HCC compared with a PRS in the lowest 80%. Whether patients with cirrhosis and a high PRS should undergo screening for HCC at shorter intervals compared with patients with similar environmental risk factors and a low PRS warrants further investigation.

Several of the genes identified in the present GWAS had lower transcriptional activity in HCC tissue than in non-tumor hepatic tissues. This was most notable for *HSD17B13*. Other genes with lower expression in HCC included *MTARC1*, *TM6SF2*, and *PNPLA3*. It is unclear whether these associations reflect causality. We speculate that at least some of the associations reflect that HCC leads to loss of normal hepatocyte function. Of the downregulated genes, *HSD17B13* is exclusively expressed in the liver, while *PNPLA3*, *MTARC1*, and *TM6SF2* have high liver expression. All four genes have a role in hepatic lipid metabolism. The HCC-associated downregulation of these four genes is likely to reflect loss of normal metabolic functions due to the dedifferentiation of hepatocytes to cancer cells in HCC. In support of this notion, we found that a panel of 541 liver-specific genes had substantially lower transcriptional activity in HCC compared with normal liver tissue.

Our study has limitations that should be considered. First, the heterogenous nature of the included cohorts and HCC cases is a limitation. This was particularly evident in the comparison between European-ancestry and Asian cohorts, where MASLD and alcohol were major risk factors in the former, while chronic hepatitis predominated in the latter. This fundamental difference was reflected in the HCC-associated genetic loci identified, with established MASLD loci enriched in Europeans and loci associated with susceptibility to chronic viral hepatitis enriched in Asians. We did not have access to information about antiviral treatment status for the participants with chronic hepatitis. Given that participants in the cohorts were enrolled from ~2000 to 2025, and that cases could be defined retrospectively via registry codes, some of the chronic hepatitis cases are likely to have been diagnosed before the advent of modern antiviral drugs. Another source of heterogeneity is that the designs of the cohorts ranged from prospective general population cohorts to cross-sectional hospital-based cohorts to case-control studies. A limitation of the risk-group analyses in the UKB was that some of the subgroups were relatively small, limiting statistical power. This was most notable for the chronic hepatitis subgroup (n = 202). The lack of association between risk alleles and increased HCC risk in patients with viral hepatitis should be interpreted cautiously and may reflect insufficient power, rather than true heterogeneity. However, Hassan *et al.* also found that variants in *TM6SF2* and *PNPLA3* were not significantly associated with HCC in patients with HCV.[5] Misclassification of cases and controls is an inherent limitation to ICD-defined case definitions. Moreover, individuals defined as controls in the prospective cohorts may have developed HCC if followed for longer. These limitations related to misclassification are likely minor, considering that HCC is a hard clinical endpoint with well-defined diagnostic criteria and the long follow-up in the included cohorts (*i.e.* UKB currently has a median follow-up of 14 years). In any case, misclassification would bias associations toward the null hypothesis and, thus, cannot explain the positive associations reported here. Finally, while our GWAS represents one of the largest HCC GWASs to date, the number of HCC cases, although substantial, still limits the statistical power to detect variants with smaller effects, as well as the effects of rare variants. Therefore, future studies with larger sample sizes are required.[39] Notably, the overall heritability of HCC remains unclear. Hassan *et al.* reported heritability estimates of HCV-negative and HCV-positive HCC of 26% and 30%, respectively.[5] Another study reported an estimated heritability of 6.3% in HBV-associated HCC.[40] Among 124 monozygotic twins of patients with HCC, none developed the disease, suggesting a modest contribution of germline genetic variation to this cancer. However, the small number of cases and absent information on shared exposures limit the interpretation of this twin study.[41]

In conclusion, we identified and validated nine genetic variants to be associated with HCC. These results expand the catalog of genes to interrogate mechanistically in future studies. A deeper insight into the genetic factors that underpin HCC may improve our ability to predict and ultimately prevent or treat this deadly cancer.

Affiliations

[1]Department of Cardiology, Rigshospitalet, Copenhagen University Hospital, Copenhagen, Denmark; [2]Cardiac Genetics Group, Department of Biomedical Sciences, University of Copenhagen, Copenhagen, Denmark; [3]Department of Clinical Biochemistry, Rigshospitalet, Copenhagen University Hospital, Copenhagen, Denmark; [4]Biotech Research & Innovation Centre (BRIC), Department of Health and Medical Sciences, University of Copenhagen, Copenhagen, Denmark; [5]deCODE genetics/Amgen, Inc, Reykjavik, Iceland; [6]Section of Digestive Diseases, Department of Internal Medicine, Yale School of Medicine, New Haven, CT, USA; [7]Department of Genetics, Yale School of Medicine, New Haven, CT, USA; [8]Department of Pathology, Yale School of Medicine, New Haven, CT, USA; [9]Department of Clinical Immunology, Rigshospitalet, Copenhagen University Hospital, Copenhagen, Denmark; [10]Novo Nordisk Foundation Center for Basic Metabolic Research, Faculty of Health and Medical Science, Copenhagen University, Copenhagen, Denmark; [11]Department of Clinical Immunology, Aarhus University Hospital, Aarhus, Denmark; [12]Department of Clinical Immunology, Odense University Hospital, Odense, Denmark; [13]Department of Clinical Immunology, Aalborg University Hospital, Aalborg, Denmark; [14]Department of Clinical Immunology, Zealand University Hospital, Køge, Denmark; [15]Translational Disease Systems Biology, Novo Nordisk Foundation Center for Protein Research, Faculty of Health and Medical Sciences, University of Copenhagen, Copenhagen, Denmark; [16]Department of Obstetrics and Gynaecology, Copenhagen University Hospital Hvidovre, Hvidovre, Denmark; [17]Statens Serum Institut, Copenhagen, Denmark; [18]Estonian Genome Centre, Institute of Genomics, University of Tartu, Tartu, Estonia; [19]Intermountain Health, Salt Lake City, UT 84111, USA; [20]Gastro Unit, Copenhagen University Hospital Hvidovre, Hvidovre, Denmark; [21]Department of Clinical Medicine, University of Copenhagen, Copenhagen, Denmark; [22]Department of Oncology, Copenhagen University Hospital-Herlev and Gentofte, Herlev, Denmark; [23]Faculty of Medicine, University of Iceland, Reykjavik, Iceland; [24]Internal Medicine and Emergency Services, Landspitali – The National University Hospital of Iceland, Reykjavik, Iceland; [25]Faculty of Electrical and Computer Engineering, University of Iceland, Reykjavik, Iceland; [26]School of Engineering and Natural Sciences, University of Iceland, Reykjavik, Iceland; [27]Department of Oncology, Rigshospitalet, Copenhagen University Hospital, Copenhagen, Denmark; [28]Hepatic Malignancy Surgical Research Unit (HEPSURU), Department of Surgery and Transplantation, Rigshospitalet, Copenhagen University Hospital, Copenhagen, Denmark

Abbreviations

ALT, alanine transaminase; AST, aspartate transaminase; CA, cross-ancestry; CHB-CC/DBDS, Copenhagen Hospital Biobank Cancer Cohort and Danish Blood Donor Study; CHB, chronic HBV; Chr, chromosome; EA, East Asian ancestry; EA, effect allele; EstBB, Estonian Biobank; EUR, European ancestry; GGT, gamma-glutamyl transferase; GWAS, genome-wide association study; HCC, hepatocellular carcinoma; HCC, hepatocellular carcinoma; IVs, instrumental variables; IVW, inverse-variance weighted; LD, linkage disequilibrium; MASLD, metabolic dysfunction-associated steatotic liver disease; MR, Mendelian randomization; MRI, magnetic resonance imaging; MVMR, multivariable Mendelian randomization; PCs, principal components; PDFF, proton density fat fraction; PLCO, The Prostate, Lung, Colorectal and Ovarian Cancer Screening Trial; PRS, polygenic risk score; RNA-seq, RNA-sequencing; RSEM, RNA-Seq by Expectation Maximization; SNP, single nucleotide polymorphism; UKB, UK Biobank; VLDL, very low-density lipoprotein.

Financial support

This research was conducted using the UKB resource under application 43247. This work was supported by BRIDGE—Translational Excellence Program (NNF20SA0064340 to JG), Beckett Fonden (23-2-10636 to JG), Independent Research Fund Denmark (9060-00012B to SS), The Innovation Fund Denmark (PM Heart to HB), NordForsk (to HB), Villadsen Family Foundation (to HB), The Arvid Nilsson Foundation, and Novo Nordisk Foundation (grants NNF17OC0027594 and NNF14CC0001 to KB and SB; NNF22OC0074956 to JBA; NNF22OC0075038 to SS). The All of Us Research Program is supported by the National Institutes of Health (NIH), Office of the Director: Regional Medical Centers: 1 OT2 OD026549; 1 OT2 OD026554; 1 OT2 OD026557; 1 OT2 OD026556; 1 OT2 OD026550; 1 OT2 OD 026552; 1 OT2 OD026553; 1 OT2 OD026548; 1 OT2 OD026551; 1 OT2 OD026555; IAA #: AOD 16037; Federally Qualified Health Centers: HHSN 263201600085U; Data and Research Center: 5 U2C OD023196; Biobank: 1 U24 OD023121; The Participant Center: U24 OD023176; Participant Technology Systems Center: 1 U24 OD023163; Communications and Engagement: 3 OT2 OD023205; 3 OT2 OD023206; and Community Partners: 1 OT2 OD025277; 3 OT2 OD025315; 1 OT2 OD025337; 1 OT2 OD025276. The research was also supported by NIH HHS under grant 5T32GM136651-03 (to JB). This work was also supported by the NIH/NIDDK (R01 DK131033-01A1 to SV). The Estonian Biobank was funded by the European Union through the European Regional Development Fund (project 2014-2020.4.01.15-0012 GENTRANSMED) and by the Estonian Research Council (grant PRG1911). Computations were performed in the High-Performance Computing Center, University of Tartu. The funders had no role in any of the following: analysis or interpretation of data, design of the study, writing of the manuscript, or the decision to submit it for publication.

Conflicts of interest

The authors who are affiliated with deCODE genetics/Amgen declare competing financial interests as employees. JG has received lecture fee from Illumina. SV has served as consultant for Albireo and received research funding from Moderna Therapeutics, with no relevance to this study. SB is a board member for Proscion A/S and Intomics A/S. JBA has received consulting fees from AstraZeneca (Nordic), QED Therapeutics, and Flagship Pioneering as well as project funding from Incyte Corp and ADCendo (not related to this study). HB receives lecture fees from Bristol-Myers Squibb, Merck Sharp and Dohme. SS has served as consultant for Regeneron and received a lecture fee from Amgen. All other authors have no conflict of interest to declare.

Please refer to the accompanying ICMJE disclosure forms for further details.

Authors' contributions

JG, HG, TR, HB, SS: conceived the study. JG, HG, GT, JB, CK, TL, EF, DFG, SS: performed analyses in the respective cohorts. TR, SV, TL, JBA, HB, SS: supervised analyses in their respective cohorts. JG performed meta-analyses. JG, HG, CJO, AS, SS: performed analyses and created figures and tables. JG, HG, SS: drafted the manuscript. JG, HG, CJO, AS, GT, GS, VT, JB, CK, SV, TME, GA, JSB, SAR, PRL, ES, CM, JT, CE, KMD, MTB, BAJ, JTB, SB, KB, HU, TL, RM, LDN, KUK, SK, LLG, KV, ESB, MOU, PS, HH, OBP, SRO, DFG, TR, KS, UL, HP, JGH, JBA, HB, SS: interpreted the results, and reviewed and commented on the manuscript. JG, HG, SS: directly accessed and verified the underlying data reported in the manuscript.

Data availability statement

Summary association results will be made freely available to all others via the GWAS catalog (www.ebi.ac.uk/gwas/) upon publication.

Acknowledgements

We acknowledge the participants and investigators of All of Us, FinnGen, BioBank Japan, China Kadoorie Biobank, Taiwan Precision Medicine Cohort, and Million Veteran Program cohort. Expression analyses are supported, in part, by data generated by the TCGA Research Network (www.cancer.gov/tcga).

Supplementary data

Supplementary data to this article can be found online at https://doi.org/10.1016/j.jhepr.2025.101485.

References

Author names in bold designate shared co-first authorship

[1] Bray F, Laversanne M, Sung H, et al. Global cancer statistics 2022: GLOBOCAN estimates of incidence and mortality worldwide for 36 cancers in 185 countries. CA Cancer J Clin 2024;74:229–263.

[2] Vogel A, Meyer T, Sapisochin G, et al. Hepatocellular carcinoma. Lancet 2022;400:1806–1807.

[3] Reig M, Forner A, Rimola J, et al. BCLC strategy for prognosis prediction and treatment recommendation: the 2022 update. J Hepatol 2022;76:681–693.

[4] Dragani TA. Risk of HCC: genetic heterogeneity and complex genetics. J Hepatol 2010;52:252–257.

[5] Hassan MM, Li D, Han Y, et al. Genome-wide association study identifies high-impact susceptibility loci for hepatocellular carcinoma in North America. Hepatology 2024;80:87–101.

[6] Li S, Qian J, Yang Y, et al. GWAS identifies novel susceptibility loci on 6p21.32 and 21q21.3 for hepatocellular carcinoma in chronic hepatitis B virus carriers. PLoS Genet 2012;8:e1002791.
[7] **Trépo E**, **Caruso S**, **Yang J**, et al. Common genetic variation in alcohol-related hepatocellular carcinoma: a case-control genome-wide association study. Lancet Oncol 2022;23:161–171.
[8] **Buch S**, **Innes H**, Lutz PL, et al. Genetic variation in TERT modifies the risk of hepatocellular carcinoma in alcohol-related cirrhosis: results from a genome-wide case-control study. Gut 2023;72:381–391.
[9] Sato G, Shirai Y, Namba S, et al. Pan-cancer and cross-population genome-wide association studies dissect shared genetic backgrounds underlying carcinogenesis. Nat Commun 2023;14:3671.
[10] Nahon P, Bamba-Funck J, Layese R, et al. Integrating genetic variants into clinical models for hepatocellular carcinoma risk stratification in cirrhosis. J Hepatol 2023;78:584–595.
[11] Gellert-Kristensen H, Richardson TG, Davey Smith G, et al. Combined effect of PNPLA3, TM6SF2, and HSD17B13 variants on risk of cirrhosis and hepatocellular carcinoma in the general population. Hepatology 2020;72:845–856.
[12] **Bianco C**, **Jamialahmadi O**, **Pelusi S**, et al. Non-invasive stratification of hepatocellular carcinoma risk in non-alcoholic fatty liver using polygenic risk scores. J Hepatol 2021;74:775–782.
[13] Verma A, Huffman JE, Rodriguez A, et al. Diversity and scale: genetic architecture of 2068 traits in the VA Million Veteran Program. Science 2024;385:eadj1182.
[14] **Yang H-C**, **Kwok P-Y**, **Li L-H**, et al. The Taiwan precision medicine initiative: a cohort for large-scale studies. bioRxiv; 2024. https://doi.org/10.1101/2024.10.14.616932. Published online October 17.
[15] Izquierdo-Sanchez L, Lamarca A, Casta A La, et al. Cholangiocarcinoma landscape in Europe: diagnostic, prognostic and therapeutic insights from the ENSCCA Registry. J Hepatol 2022;76:1109–1121.
[16] **Sveinbjörnsson G**, **Ulfarsson MO**, Thorolfsdottir RB, et al. Multiomics study of nonalcoholic fatty liver disease. Nat Genet 2022;54:1652–1663.
[17] Ghouse J, Sveinbjörnsson G, Vujkovic M, et al. Integrative common and rare variant analyses provide insights into the genetic architecture of liver cirrhosis. Nat Genet 2024;56:827–837.
[18] MacParland SA, Liu JC, Ma X-Z, et al. Single cell RNA sequencing of human liver reveals distinct intrahepatic macrophage populations. Nat Commun 2018;9:4383.
[19] Hemani G, Tilling K, Davey Smith G. Orienting the causal relationship between imprecisely measured traits using GWAS summary data. PLoS Genet 2017;13:e1007081.
[20] Hemani G, Zheng J, Elsworth B, et al. The MR-base platform supports systematic causal inference across the human phenome. Elife 2018;7:e34408.
[21] Sanderson E, Spiller W, Bowden J. Testing and correcting for weak and pleiotropic instruments in two-sample multivariable Mendelian randomization. Stat Med 2021;40:5434–5435.
[22] Cancer Genome Atlas Research Network. Comprehensive and integrative genomic characterization of hepatocellular carcinoma. Cell 2017;169:1327–1341.
[23] Roessler S, Long EL, Budhu A, et al. Integrative genomic identification of genes on 8p associated with hepatocellular carcinoma progression and patient survival. Gastroenterology 2012;142:957–966.
[24] Cerami E, Gao J, Dogrusoz U, et al. The cBio cancer genomics portal: an open platform for exploring multidimensional cancer genomics data. Cancer Discov 2012;2:401–404.
[25] Thomas DL, Thio CL, Martin MP, et al. Genetic variation in IL28B and spontaneous clearance of hepatitis C virus. Nature 2009;461:798–801.
[26] Ishigaki K, Akiyama M, Kanai M, et al. Large-scale genome-wide association study in a Japanese population identifies novel susceptibility loci across different diseases. Nat Genet 2020;52:669–679.
[27] Natarajan Y, Patel P, Chu J, et al. Risk of hepatocellular carcinoma in patients with various HFE genotypes. Dig Dis Sci 2023;68:312–322.
[28] Abul-Husn NS, Cheng X, Li AH, et al. A protein-truncating HSD17B13 variant and protection from chronic liver disease. N Engl J Med 2018;378:1096–1106.
[29] Jamialahmadi O, Mancina RM, Ciociola E, et al. Exome-wide association study on alanine aminotransferase identifies sequence variants in the GPAM and APOE associated with fatty liver disease. Gastroenterology 2021;160:1634–1646.
[30] Emdin CA, Haas ME, Khera AV, et al. A missense variant in mitochondrial amidoxime reducing component 1 gene and protection against liver disease. PLoS Genet 2020;16:e1008629.
[31] Florez-Vargas O, Ho M, Hogshead MH, et al. Genetic regulation of TERT splicing affects cancer risk by altering cellular longevity and replicative potential. Nat Commun 2025;16:1676.
[32] Eslam M, Hashem AM, Leung R, et al. Interferon-λ rs12979860 genotype and liver fibrosis in viral and non-viral chronic liver disease. Nat Commun 2015;6:6422.
[33] Teshigawara K, Ogawa W, Mori T, et al. Role of Krüppel-like factor 15 in PEPCK gene expression in the liver. Biochem Biophys Res Commun 2005;327:920–926.
[34] Uchida S, Tanaka Y, Ito H, et al. Transcriptional regulation of the CLC-K1 promoter by myc-associated zinc finger protein and kidney-enriched Krüppel-like factor, a novel zinc finger repressor. Mol Cell Biol 2000;20:7319–7331.
[35] Petrick JL, Yang B, Altekruse SF. Risk factors for intrahepatic and extrahepatic cholangiocarcinoma in the United States: a population-based study in SEER-Medicare. PLoS ONE 2017;12:e0186643.
[36] Hwang I, Kang SY, Kim DG, et al. Clinicopathologic and genomic characteristics of biliary tract carcinomas with TERT promoter mutations among East Asian population. Pathol Res Pract 2025;266:155806.
[37] Dong R, Najjar G, Günes C, et al. Aberrant TERT expression: linking chronic inflammation to hepatocellular carcinoma. J Pathol 2025;266:130–133.
[38] Gellert-Kristensen H, Bojesen SE, Tybjærg Hansen A, et al. Telomere length and risk of cirrhosis, hepatocellular carcinoma, and cholangiocarcinoma in 63,272 individuals from the general population. Hepatology 2024;79:857–868.
[39] Vujkovic M, Kaplan D, Ghouse J, et al. Novel genetic architecture and metabolic interactions for all-cause cirrhosis and hepatocellular carcinoma defined in multi-ancestry meta-analyses. Hepatology 2024;80:S1.
[40] Jiang D, Deng J, Dong C, et al. Knowledge-based analyses reveal new candidate genes associated with risk of hepatitis B virus related hepatocellular carcinoma. BMC Cancer 2020;20:403.
[41] Mucci LA, Hjelmborg JB, Harris JR, et al. Familial risk and heritability of cancer among twins in Nordic countries. JAMA 2016;315:68–76.

Keywords: HCC; Cirrhosis; MASLD; Biliary tract cancer; PNPLA3.
Received 29 November 2024; received in revised form 4 June 2025; accepted 5 June 2025; Available online 11 June 2025

Journal of Hepatology, Volume *7*

Supplemental information

Genome-wide meta-analysis identifies nine loci associated with higher risk of hepatocellular carcinoma development

Jonas Ghouse, Helene Gellert-Kristensen, Colm J. O'Rourke, Anne-Sofie Seidelin, Gudmar Thorleifsson, Gardar Sveinbjörnsson, Vinicius Tragante, Chigoziri Konkwo, Joseph Brancale, Silvia Vilarinho, Tim M. Eyrich, Gustav Ahlberg, Johan S. Bundgaard, Søren A. Rand, Pia R. Lundegaard, Erik Sørensen, Christina Mikkelsen, Jacob Træholt, Christian Erikstrup, Khoa M. Dinh, Mie T. Bruun, Bitten Aa. Jensen, Jakob T. Bay, Søren Brunak, Karina Banasik, Henrik Ullum, DBDS Genomic Consortium, Estonian Biobank Research Team, Triin Laisk, Reedik Mägi, Lincoln D. Nadauld, Kirk U. Knowlton, Stacey Knight, Lise L. Gluud, Kirsten Vistisen, Einar S. Björnsson, Magnus O. Ulfarsson, Patrick Sulem, Hilma Holm, Ole B. Pedersen, Sisse R. Ostrowski, Daniel F. Gudbjartsson, Thorunn Rafnar, Kari Stefansson, Ulrik Lassen, Hans-Christian Pommergaard, Jens G. Hillingsø, Jesper B. Andersen, Henning Bundgaard, and Stefan Stender

Genome-wide meta-analysis identifies nine loci associated with higher risk of hepatocellular carcinoma development

Jonas Ghouse, Helene Gellert-Kristensen, Colm J. O'Rourke, Anne-Sofie Seidelin, Gudmar Thorleifsson, Gardar Sveinbjörnsson, Vinicius Tragante, Chigoziri Konkwo, Joseph Brancale, Silvia Vilarinho, Tim M. Eyrich, Gustav Ahlberg, Johan S. Bundgaard, Søren A. Rand, Pia R. Lundegaard, Erik Sørensen, Christina Mikkelsen, Jacob Træholt, Christian Erikstrup, Khoa M. Dinh, Mie T. Bruun, Bitten Aa. Jensen, Jakob T. Bay, Søren Brunak, Karina Banasik, Henrik Ullum, DBDS Genomic Consortium, Estonian Biobank Research Team, Triin Laisk, Reedik Mägi, Lincoln D. Nadauld, Kirk U. Knowlton, Stacey Knight, Lise L. Gluud, Kirsten Vistisen, Einar S. Björnsson, Magnus O. Ulfarsson, Patrick Sulem, Hilma Holm, Ole B. Pedersen, Sisse R. Ostrowski, Daniel F. Gudbjartsson, Thorunn Rafnar, Kari Stefansson, Ulrik Lassen, Hans-Christian Pommergaard, Jens G. Hillingsø, Jesper B. Andersen, Henning Bundgaard, Stefan Stender

Table of contents

Supplementary materials and methods..2

Supplementary tables...6

Supplementary figures...14

Supplementary references..21

Consortia members..23

Supplementary materials and methods

Cohort information, case-control ascertainment, ethics and genotyping and imputation details

Copenhagen Hospital Biobank Cancer Cohort (CHB-CC) and Danish Blood Donor Study (DBDS)

Case-control ascertainment: The Copenhagen Hospital Biobank (CHB) Cancer Cohort (CHB-CC) includes genome-wide genotype data for ~319,000 subjects admitted to general hospitals in the capital area of Denmark between 2009 and 2020[1]. The Danish Blood Donor Study is a cohort including blood donors from Denmark.[2] As of 2024, ~114,000 individuals with genome-wide genotype data were available. HCC cases were defined using the following ICD-10 codes: C22.0 (Liver cell carcinoma) or C22.9 (Malignant neoplasm of liver, not specified as primary or secondary).

Ethics: Since the biological samples stored in CHB-CC were based on leftover material from routine blood analyses, the patients were not asked for informed consent before inclusion. However, patients were informed by electronic mail (E-Boks) or normal mail about the research and the opt-out possibility to have their biological specimens excluded from use in research in the research conducted or in general. Thus, since 2004 a national Register on Tissue Application (Vævsanvendelsesregistret) lists all individuals who have chosen to opt out and whose samples cannot be used for research purposes. Before initiating this study, individuals listed in the Register on Tissue Application were excluded. For DBDS, informed consent was obtained from all participants. Both CHB- CC and DBDS are approved by the National Committee on Health Research Ethics (NVC 1708829 and NVC 1700407) and the Danish Data Protection Agency (P-2019-93 and P-2019-99).

Genotyping and imputation: Samples from 276,114 Danes from the CHB and DBDS were genotyped using Illumina Global Screening Array chips and long-range phased together with ~238,000 genotyped samples from North-western Europe using Eagle. Samples and variants with less than 98% yield were excluded. A haplotype reference panel was prepared in the same manner as for the Icelandic data (see below) by phasing whole-genome sequence genotypes of 15,576 individuals from Scandinavia, the Netherlands, and Ireland using the phased chip data. Graphtyper was used to call the genotypes which were subsequently imputed into the phased chip data. Whole genome sequencing, chip-typing, quality control, long-range phasing, and imputation from which the data for this analysis were generated was performed at deCODE genetics.

deCODE

Case-control ascertainment: Data from the deCODE study included 413 HCC cases and 375,109 controls[3]. Cases were defined using the ICD-10 code C22.0. The information on HCC diagnosis is obtained from the Icelandic Cancer Registry (ICR).[4] In this study we utilize registered diagnoses from 1955 to 2022.

Ethics: The study was approved by the National Bioethics Committee (VSN-18-148) and the Icelandic Data Protection Authority. Written informed consent was obtained from all participants who donated blood samples to research. All sample identifiers were encrypted in accordance with the regulations of the Icelandic Data Protection Authority.

Genotyping and imputation: The genome of the Icelandic population was characterized by whole-genome sequencing of 49,708 Icelanders using Illumina standard TruSeq methodology to a mean depth of 35x (SD 8x) with subsequent long-range phasing,[5] and imputing the information into 166,281 individual chip-genotyped employing multiple Illumina platforms.[6] Using genealogic information, we further imputed sequence variants into 285,664 relatives of the genotyped individuals to increase the sample sizes and power to detect associations. In total, we characterized up to 35.3 million variants in the Icelandic population with imputation score > 0.8.

Intermountain Healthcare

Case-control ascertainment: The HerediGene Population study is a large-scale collaboration between Intermountain Healthcare, deCODE genetics, and Amgen, Inc.[3] Participants in the HerediGene Population study are voluntary US residents over the age of 18 years, who gave permission to link anonymized genotypic data with EHRs. Cases were defined using the following ICD-10 codes: C22.0. The remaining individuals of the Intermountain Healthcare cohort were included as controls. In total, 81 cases and 26,768 controls were identified.

Ethics: The Intermountain Healthcare Institutional Review Board approved this study, and all participants provided written informed consent prior to enrollment.

Genotyping and imputation: The Intermountain dataset was derived from a cohort created by whole-genome sequencing of 16,661 Americans of European ancestry living in Utah, to an average coverage of at least 20x, performed at deCODE genetics. These samples served as a reference panel for long-range phasing and imputation of 60,397 chip-typed individuals enrolled at multiple Intermountain Healthcare facilities.

UK Biobank

Case-control ascertainment: The UK Biobank (UKB) is a prospective cohort of more than 500,000 individuals living in the United Kingdom who were 40-79 years of age at recruitment (2006-2010).[7] The median age at enrollment was 58 years and median follow-up time in 2024 was ~14 years. Cases were defined using the following ICD-10 codes: C22.0. All other participants were set as controls. Data from the UKB included 519 HCC cases and 417,528 controls.

Ethics: The UK Biobank cohort has been approved by the Northwest Multicenter Research Ethics Committee, UK (Ref: 16/NW/0274). Written informed consent has been obtained from all study participants.

Genotyping and imputation: Genotypic data were available for 488,380 individuals and were imputed to the HRC, UK10K and 1,000 Genomes Phase 3 reference panels using IMPUTE4 to identify ≈ 93M variants for 487,409 individuals. Using the genotyped SNPs, persons were excluded if they had: high levels of missingness or heterozygosity, SNP genotype call rate < 98%, or if phenotypic and genotypic gender information was discordant.

Estonian Biobank

Case-control ascertainment: The Estonian Biobank is a volunteer-based sample of the Estonian adult population aged ≥18 years[8]. Baseline measurements included a standardized health examination, health-related questionnaires, blood samples for DNA, white blood cells and plasma tests, and clinical diagnoses defined by ICD-10 codes. HCC cases were defined using the following ICD-10 codes: C22.0 or C22.9 extracted from health records. Median follow-up was ~10 years in 2024.

Ethics: Analyses in the Estonian Biobank were carried out under ethical approval 1.1-12/624 from the Estonian Committee on Bioethics and Human Research (Estonian Ministry of Social Affairs), using data according to Estonian Biobank release 6-1/GI/47. All participants provided written informed consent.

Genotyping and imputation: At present, more than 200,000 participants have undergone genotyping by a genome-wide SNP array which includes more than 700,000 SNPs (Illumina GSA microchip).

FinnGen Freeze 10

Case-control ascertainment: The summary statistics for 500 cases and 314,193 controls from Finngen were downloaded from https://www.finngen.fi/en/access_results. Cases were defined using the following ICD-10 codes: ICD10: C22.0.

Ethics: Patients and control subjects in FinnGen provided informed consent for biobank research, based on the Finnish Biobank Act. Separate research cohorts, gathered before the Finnish Biobank Act was initialized (in September 2013) and start of FinnGen (August 2017), were collected based on study-specific consents and later assigned to the Finnish biobanks after approval by Fimea, the National Supervisory Authority for Welfare and Health. The Coordinating Ethics

Committee of the Hospital District of Helsinki and Uusimaa (HUS) approved the FinnGen study protocol Nr HUS/990/2017. The FinnGen study is approved by Finnish Institute for Health and Welfare (THL), approval number THL/2031/6.02.00/2017, amendments THL/1101/5.05.00/2017, THL/341/6.02.00/2018, THL/2222/6.02.00/2018, THL/283/6.02.00/2019, THL/1721/5.05.00/2019, Digital and population data service agency VRK43431/2017-3, VRK/6909/2018-3, VRK/4415/2019-3 the Social Insurance Institution (KELA) KELA 58/522/2017, KELA 131/522/2018, KELA 70/522/2019, KELA 98/522/2019, and Statistics Finland TK-53-1041-17.7

Genotyping and imputation: A custom-made FinnGen ThermoFisher Axiom array (>650,000 SNPs) was used to genotype FinnGen samples. Genotype calls were made with AxiomGT1 algorithm. Individuals with ambiguous gender, high genotype missingness (>5%), excess heterozygosity (±4 SD), and non-Finnish ancestry were excluded. Variants with high missingness (>2%), low Hardy-Weinberg equilibrium ($< 1\times10^{-6}$), or low minor allele count (<3) were excluded. High coverage (25–30×) WGS data were used to develop the Finnish population-specific SISu v3 imputation reference panel with Beagle 4.1. More than 16 million variants have been imputed (https://finngen.gitbook.io/documentation/methods/genotype-imputation).

All of US

Case-control ascertainment: A total of 256 cases with cirrhosis and 120,594 controls were included from the All o fUS cohort[9]. HCC cases were defined using SNOMED-25370001.

Ethics: Ethical approval was received from National Institute of Health All of Us Institutional Review Board. Participants provided written informed consent.

Genotyping and imputation: Genotyping was by whole genome sequencing (Illumina Whole Genome Sequencing. All of Us Research and Data Center whole genome sequencing Hail Matrix Table (v.6) was imported into Hail (v. 0.2.107). Samples were stratified by self-reported race or ethnicity into Black, Hispanic, and White subgroups. Only individuals of White European ancestry were included in our study owing to too few samples in the other subgroups. Samples were filtered for heterozygosity, sex mismatch, and genetic-ancestry outliers.

French/Belgian HCC GWAS (Trépo et al)

Case-control ascertainment: The summary statistics from a previously published GWAS[10] of 775 HCC cases and 1332 controls (all self-reported French or Belgian ancestry) were downloaded from the GWAS catalog at https://www.ebi.ac.uk/gwas/studies/GCST90092003. Cases were defined as patients with HCC based on imaging or histology on a background of alcohol-related cirrhosis. Control individuals had alcohol-related liver disease but no HCC.

Ethics: Patients gave written, informed consent and the study received approval from the ethics committees of all participating centers.

Genotyping and imputation: Genotyping was done with the Global Screening Array, version 1.0 (Illumina; San Diego, CA, USA), which contains 618,564 variants before quality control. Samples that had genetic sex discordance, genotype call rates less than 95% or outlying heterozygosity for autosomal chromosomes (i.e., ±3 SDs away from the sample mean), or had an estimated identity by descent value of more than 0·1875 were excluded. Ancestry was identified by principal component analysis, and individuals with non-European ancestry were excluded because of small numbers. SNPs with a genotype call rate lower than 95%, a minor allele frequency less than 0·1%, different missing genotype rates in cases and controls (p<10E−5), and that did not meet Hardy-Weinberg equilibrium (p<10E−6) in controls were excluded. Based on the genotyped SNPs that passed the above-described quality control, a total of 7,962,325 SNPs were imputed.

PLCO

Case-control ascertainment: The summary statistics from 151 HCC cases and 56,413 controls from The Prostate, Lung, Colorectal and Ovarian (PLCO) Cancer Screening Trial[11] were downloaded from https://exploregwas.cancer.gov/plco-atlas/#/downloads

Ethics: The PLCO Cancer Screening Trial is a randomized, controlled trial to determine whether certain screening exams reduce mortality from prostate, lung, colorectal and ovarian cancer. Approximately 155,000 participants were enrolled between November 1993 and July 2001. PLCO has the following five ClinicalTrials.gov registration numbers: NCT00002540 (Prostate), NCT01696968 (Lung), NCT01696981 (Colorectal), NCT01696994 (Ovarian), and NCT00339495 (EEMS). All participants provided written informed consent.

Genotyping and imputation: Genotyping was by Illumina arrays: Global Screening Array (GSA) OncoArray, Omni2.5 M, or OmniExpress (OmniX). Quality control included filtering for high missingness of phenotypes, low genotype call rate, and checks for sex mismatches, contamination, assay concordance, duplicates, relatedness, and abnormal heterozygosity[11]. The TopMed reference panel was used for genotype imputation.

Biobank Japan

Case-control ascertainment: The summary statistics for 2,122 cases and 159,201 controls from Biobank Japan[12] were downloaded from https://pheweb.jp/pheno/HepC. Cases were defined using ICD-10 code C22.0 or phecode 155.1, as described in Sakaue et al.[13]

Ethics: Biobank Japan received ethical approval from Research ethics committees at the Institute of Medical Science, the University of Tokyo, the RIKEN Yokohama Institute, and the 12 cooperating hospitals. All participants provided written informed consent.

Genotyping and imputation: Genotyping was done using the Illumina HumanOmniExpressExome BeadChip or a combination of the Illumina HumanOmniExpress and HumanExome BeadChips. Quality control (QC) of samples included exclusion of those with call rate < 0.98 and outliers from East Asian clusters identified by principal component analysis. For QC of genotypes, variants meeting any of the following criteria were excluded: (i) call rate < 99%, (ii) P value for HWE < 1.0×10^{-6}, and (iii) number of heterozygotes less than five. Using 939 samples whose genotypes were also analyzed by whole genome sequencing (WGS), additional QC was done based on the concordance rate between genotyping array and WGS. Variants with a concordance rate < 99.5% or a non-reference discordance rate ≥ 0.5% were excluded. Imputation was by SHAPEIT (v2.778) and minimac3 (v2.0.1) and used data from 1000 Genomes Project Phase 3 (version 5) as a reference.

China Kadoorie Biobank

Case-control ascertainment: The summary statistics from 670 cases with HCC and 76,022 controls from China Kadoorie Biobank[14] were downloaded from https://pheweb.ckbiobank.org/pheno/c22. Cases were defined by ICD10 code C22.

Ethics: All participants provided written informed consent. Ethical approval was obtained from the Oxford Tropical Research Ethics Committee, the Ethical Review Committees of the Chinese Center for Disease Control and Prevention, Chinese Academy of Medical Sciences, and the Institutional Review Board (IRB) at Peking University. The Chinese Ministry of Health approved the study at the start in 2004 (including export of plasma samples to Oxford) and approved electronic linkage to health insurance records in 2011. Raw genotyping data were exported from China to the Oxford CKB International Coordinating Center under Data Export Approvals 2014-13 and 2015-39 from the Office of Chinese Human Genetic Resource Administration.

Genotyping and imputation: Genotyping was done by a custom-designed Affymetrix Axiom array. Genotyping and QC followed Affymetrix best practice workflow. Imputation was performed based on EAS population data in the 1000 Genomes Phase 3 reference.

Supplementary tables

Table S1. Cohorts, phenotyping, genotyping, statistical analysis, and references.

Cohort	N cases	N controls	HCC definition	Genotyping platform	Statistical analysis			Ref.
					Software	Model	Adjustments	
CHB-CID/DBDS	917	355919	ICD10: C22.0 or C22.9	Illumina Human Global Screening Array and Illumina Human Omni Screening Array	Regenie v2.2.4	Logistic	Year of birth, sex and first 10 PCs	1
deCODE genetics	413	375109	ICD10: C22.0	Illumina HumanHap300, HumanCNV370, HumanHap610, HumanHap1M, HumanHap660, Omni-1, Omni 2.5 or Omni Express bead chips	deCODE's	Logistic	Sex, year of birth, country, lifespan, first 10 PCs	3
UK Biobank	519	417528	ICD10: C22.0	UK BiLEVE Axiom Array/ UK Biobank Axiom Array	Regenie v2.2.4	Logistic	Year of birth, sex and first 10 PCs	7
Finngen R10	500	314193	ICD10: C22.0	Illumina chip arrays<br>Affymetrix chip arrays	SAIGE 0.36.3.2	Logistic	Age, sex, genotype batch and first 10 PCs	15
AllofUS (European)	256	120594	ICD10: C22.0, C22.8, C22.9	Illumina Whole Genome Sequencing	Hail	Logistic	Age, sex, first 10 PCs	9
Trépo et al	775	1332	Imaging or histology	Illumina Human Global Screening Array	Plink v. 1.9	Logistic	First 10 PCs	10
Estonian Biobank	136	193680	ICD10: C22.0 or C22.9	Illumina Human Global Screening Array	Regenie v3.0.3	Logistic	Age at enrollment, sex, 10 PCs	8
Intermountain	81	77000	ICD10: C22.0	Illumina Global Screening array family	deCODE's	Logistic	Year of birth, sex and first 10 PCs	3
PLCO	151	56413	ICD10: C22.0	OncoArray, Omni2.5 M, OmniExpress, and Illumina Global Screening Array	SAIGE 0.43.2	Logistic	Age, sex, study center, and top 20 PCs	11
BioBank Japan	2122	159201	ICD10: C22.0<br>phecode: 155.1	Illumina HumanOmniExpressExome v.1.0/v.1.2, HumanOmniExpress v.1.0 and Human Exome BeadChip v.1.0/v.1.1	Plink v. 2.0.0	Logistic	Age, sex and first 20 PCs	12
Kadoorie Biobank	670	76022	ICD10: C22	Custom-designed Affymetrix Axiom	SAIGE 0.42.1	Logistic	Array, sex, age, age^2, region, first 11 PCs	14

Abbreviations: HCC; Hepatocellular carcinoma, Ref; reference, CHB-CC/DBDS; Copenhagen Hospital Biobank Cancer Cohort and Danish Blood Donor Study, PLCO; The Prostate, Lung, Colorectal and Ovarian (PLCO) Cancer Screening Trial.

Table S2. Baseline characteristics of hepatocellular carcinoma cases in the included cohorts.

Cohort	N cases	Male (%)	Age (y)	Obese (%)	Smoking (%)	Alcohol drinker (%)	Chronic viral hepatitis (%)
CHB-CID/DBDS	917	78	73	-	-	-	-
deCODE genetics	413	69	-	47	79	-	5
UK Biobank	519	76	70	49	73	97	7.5
Finngen R10	500	88	73	-	-	-	~1
AllofUS (European)	256	57	64	26	29	22	30
Trépo et al	775	90	65	-	-	100	0
Estonian Biobank	136	50	-	-	-	-	17
Intermountain	81	-	-	-	-	-	-
PLCO	151	89	-	-	-	-	-
BioBank Japan	2122	76	68	23	M: 82, F: 24	M: 79, F: 34	HBV: M: 12, F: 6<br>HCV: M: 44, F: 53
Kadoorie Biobank	670	63	~60	4	52	31	22 (HBsAg+)

Grey cells indicate that the information was not available. Smoking and alcohol was defined as current smoking or current alcohol consumer. Obese was defined as participants with a body mass index of more than 30 kg/m^2. Chronic viral hepatitis was defined by ICD-code except in BioBank Japan and Kadoorie Biobank, where serology was used. Baseline information of HCC cases in BioBank Japan and Kadoorie Biobank was extracted from published studies[16,17].

Table S3. Frequencies of the hepatocellular carcinoma variants in individuals of European and East Asian ancestry.

CHR	Position	Gene	Location	rsnumber	EA	NEA	EAF_EUR	EAF_EA	EAF_EUR / EAF_EA
**European ancestry GWAS meta-analysis**									
1	220973563	*MTARC1*	Intronic	rs2642442	T	C	0.6974	0.8083	0.86
3	126095012	*KLF15*	Intergenic	rs7628416	C	G	0.2413	0.6232	0.39
4	88221345	*HSD17B13*	Intergenic	rs4089	C	G	0.7252	0.6516	1.11
5	1279790	*TERT*	Intronic	rs10069690	T	C	0.2704	0.1553	1.74
6	26072992	*HFE*	Intergenic	rs144861591	T	C	0.0588	0.0006	98.0
19	19379549	*TM6SF2*	Exonic	rs58542926	T	C	0.0753	0.0697	1.08
19	45411941	*APOE*	Exonic	rs429358	T	C	0.8361	0.9115	0.91
22	44324730	*PNPLA3*	Exonic	rs738408	T	C	0.2239	0.3812	0.59
**East Asian ancestry GWAS meta-analysis**									
6	33035974	*HLA-DPA1*	UTR3	rs3179778	A	G	0.1626	0.6088	0.27
19	39737866	*IFNL4*	Exonic	rs12971396	C	G	0.1993	0.0909	2.19

Abbreviations: CHR, chromosome; EA, effect allele; NEA, non-effect allele; EAF_EUR, effect allele frequency in Europeans; EAF_EA, effect allele frequency in East Asians; EAF_EUR / EAF_EA, the ratio between EAF_EUR and EAF_EA.

Table S4. Look-up of genetic variants previously implicated in hepatocellular carcinoma and not identified in the present GWAS.

CHR	Position	Rs-number	Effect allele	Non-effect allele	Effect allele frequency	Gene	Beta	Standard error	P-value
1	228192753	rs708113	A	T	0.65	*WNT3A-WNT9A*	0.01	0.02	0.61
2	27730940	rs1260326	T	C	0.5	*GCKR*	0.04	0.022	0.07
3	39600177	rs9842969	T	C	0.09	*MOBP*	-0.03	0.045	0.53
4	103188709	rs13107325	T	C	0.06	*SLC39A8*	-0.1	0.059	0.10
5	1280028	rs2242652	A	G	0.21	*TERT*	-0.19	0.03	**2.1×10^{-9}**
6	32659878	rs9275224	A	G	0.37	*HLA-DQB1*	0.08	0.03	**6.3×10^{-3}**
8	9183596	rs4841132	A	G	0.09	*PPP1R3B*	-0.1	0.056	0.07
14	94844947	rs28929474	T	C	0.02	*SERPINA1*	0.45	0.089	**5.5×10^{-7}**
19	54676763	rs641738	T	C	0.42	*MBOAT7*	0.11	0.028	**1.5×10^{-4}**

Abbreviations: CHR, chromosome.

Table S5. Replication of HCC loci in the Million Veteran Program Cohort and the Taiwan Precision Medicine Initiative cohort.

						**GWAS meta-analysis (EUR)** 3,748 cases 1,861,536 controls			**Million Veteran Program (EUR)** 2,852 cases 447,587 controls				
**CHR**	**Position**	**Gene**	**rsnumber**	**EA**	**NEA**	**Beta**	**SE**	**P-val**	**Beta**	**95% CI lower**	**95% CI upper**	**P-val**	**Directional concordance?**
1	220973563	*MTARC1*	rs2642442	T	C	0.1735	0.0295	3.98E-09	0.063	0.006	0.119	0.03	yes
3	126095012	*KLF15*	rs7628416	C	G	0.1701	0.0302	1.77E-08	-0.014	-0.081	0.0526	0.68	no
4	88221345	*HSD17B13*	rs4089	C	G	0.2121	0.0286	1.29E-13	0.191	0.130	0.252	7.4E-10	yes
5	1279790	*TERT*	rs10069690	T	C	-0.2085	0.029	6.88E-13	-0.113	-0.176	-0.053	2.6E-4	yes
6	26072992	*HFE*	rs144861591	T	C	0.4533	0.0494	4.37E-20	0.218	0.121	0.316	1.2E-5	yes
19	19379549	*TM6SF2*	rs58542926	T	C	0.6074	0.04	3.36E-52	0.389	0.305	0.474	4.9E-18	yes
19	45411941	*APOE*	rs429358	T	C	0.2691	0.0362	1.07E-13	0.203	0.122	0.284	7.5E-7	yes
22	44324730	*PNPLA3*	rs738408	T	C	0.5538	0.027	2.69E-93	0.403	0.345	0.460	5.4E-40	yes
						**GWAS meta-analysis (East Asian)** 2,792 cases 235,223 controls			**Taiwan Precision Medicine Initiative** 4,778 cases 286,102 controls				
6	33035974	*HLA-DPA1*	rs3179778	A	G	0.2084	0.0281	1.34E-13	0.17	0.121	0.219	1.2E-12	yes
19	39737866	*IFNL4*	rs12971396*	C	G	0.314	0.05	3.40E-10	0.095	0.003	0.187	0.04	yes

The summary statistics for the outcome 'Phe_155_1: Malignant neoplasm of liver, primary' in the Million Veteran Program (MVP) cohort were downloaded from https://ftp.ncbi.nlm.nih.gov/dbgap/studies/phs002453/analyses/GIA/. Associations for the same endpoint were looked up in the Taiwan Precision Medicine Initiative Cohort at https://pheweb.ibms.sinica.edu.tw/. Directional concordance refers to concordance in directions of beta values between the GWAS and the validation cohort. Abbreviations: EA, effect allele; EUR, European ancestry; NEA, non-effect allele; SE, standard error; CI, confidence interval. * A proxy variant in high linkage disequilibrium (rs1042434, r^2=0.94 with rs12971396 in East Asian populations) was used for validating this variant in the Taiwan Precision Medicine Initiative cohort.

Table S6. Human liver single-cell sequencing transcriptomics of the HCC-associated genes.

Cell type	MARC1	KLF15	HSD17B13	TERT	HFE	TM6SF2	APOE	PNPLA3	HLA-DPA1	IFNL4
Hepatocyte_1	0.08	0.18	0.47	0	0.03	0.14	5.42	0.12	0.01	NA
Hepatocyte_2	0.09	0.21	0.42	0	0.03	0.01	3.58	0.13	0.01	NA
Hepatocyte_3	0.12	0.26	0.03	0	0.09	0.01	0.57	0.17	0.06	NA
Hepatocyte_4	0.11	0.25	0.41	0	0.03	0.17	5.22	0.14	0	NA
Hepatocyte_5	0.08	0.16	0.51	0	0.03	0.09	5.51	0.15	0.06	NA
Hepatocyte_6	0.12	0.09	0.13	0	0	0.04	6.09	0.04	0.07	NA
Cholangiocytes	0.01	0.06	0.01	0	0.01	0	0.08	0.04	0.11	NA
Hepatic_Stellate_Cells	0	0.01	0	0	0.03	0	1.02	0	0.34	NA
Periportal_LSECs	0.02	0.03	0.01	0	0.01	0.01	0.54	0	0.15	NA
Central_venous_LSECs	0.02	0.01	0.01	0	0.03	0	0.55	0	0.16	NA
Portal_endothelial_Cells	0	0.03	0.03	0	0.03	0	0.61	0	0.32	NA
Plasma_Cells	0.01	0.01	0	0	0.02	0	0.77	0	0.35	NA
Erythroid_Cells	0	0.01	0.13	0	0.03	0	2.84	0	0.23	NA
alpha-beta_T_Cells	0	0.01	0.01	0	0	0	0.77	0	0.45	NA
gamma-delta_T_Cells_1	0	0.01	0.02	0	0	0	0.89	0	0.32	NA
gamma-delta_T_Cells_2	0	0	0.01	0	0	0	0.53	0	0.03	NA
NK-like_Cells	0	0.01	0.01	0	0	0	0.74	0	0.14	NA
Non-inflammatory_Macrophage	0	0.02	0	0	0.16	0	1.63	0.02	2.19	NA
Mature_B_Cells	0	0	0	0	0	0	0.73	0	0.23	NA
Inflammatory_Macrophage	0.08	0.01	0.01	0	0.02	0	1.08	0	1.78	NA

The values are mean normalized single-cell transcriptomic data from MacParland et al.[18], based on 8444 cells from five human livers. *IFNL4* is not expressed in the liver and therefore not included in the dataset. The fill colors depict hepatocytes (light brown), cholangiocytes (forest green), hepatic stellate cells (yellow), endothelial cells (light green), and immune cells (light blue). LSEC: liver sinuisodal endothelial cells.

Table S7. Associations of GWAS-identified HCC-associated variants with incident HCC in at-risk subgroups in the UK Biobank.

				BMI ≥ 30 kg/m² nTotal = 98,737 nHCC = 279		High alcohol intake nTotal = 87,991 nHCC = 173		Type 2 diabetes nTotal = 6,992 nHCC = 73		Cirrhosis nTotal = 236 nHCC = 40		Hepatitis C nTotal = 167 nHCC = 20		Chronic hepatitis B + C nTotal = 202 nHCC = 22	
Genetic variant	EA	RA	GWAS OR	HR (95% CI)	P	HR (95% CI)	P	HR (95% CI)	P	HR (95% CI)	P	HR (95% CI)	P	HR (95% CI)	P
rs2642442	T	C	1.19	1.42 (1.17-1.72)	0.0003	1.4 (1.1-1.8)	0.006	1.4 (0.98-2.1)	0.06	1.33 (0.83-2.14)	0.24	0.66 (0.37-1.34)	0.25	0.76 (0.40-1.47)	0.42
rs7628416	C	G	1.19	1.24 (1.02-1.51)	0.03	1.4 (1.1-1.8)	0.005	0.84 (0.54-1.29)	0.4	0.85 (0.46-1.56)	0.60	1.36 (0.61-3.05)	0.46	1.84 (0.91-3.73)	0.09
rs4089	C	G	1.23	1.22 (1.01-1.48)	0.04	1.14 (0.89-1.4)	0.3	2.1 (1.36-3.29)	0.0009	1.87 (1.01-3.46)	0.04	1.18 (0.57-2.44)	0.65	1.1 (0.55-2.15	0.80
rs10069690	T	C	0.81	0.7 (0.56-0.86)	0.0006	0.69 (0.52-0.9)	0.006	0.77 (0.51-1.14)	0.19	0.92 (0.54-1.57)	0.76	0.77 (0.32-1.85)	0.56	0.76 (0.34-1.69)	0.50
rs144861591	T	C	1.57	1.19 (0.89-1.6)	0.2	1.43 (1.02-2.02)	0.04	1.85 (1.17-2.92)	0.0086	1.29 (0.78-2.13)	0.32	1.96 (0.76-5.05)	0.16	2.32 (0.92-5.83)	0.07
rs58542926	T	C	1.84	2.35 (1.87-2.96)	$3.3*10^{-13}$	2.59 (1.95-3.43)	$4.4*10^{-11}$	2.58 (1.71-3.91)	$7.3*10^{-6}$	2.1 (1.07-4.12)	0.03	1.27 (0.35-4.56)	0.71	1.14 (0.32-4.07)	0.84
rs429358	T	C	1.31	1.41 (1.08-1.8)	0.01	1.61 (1.13-2.28)	0.008	1.64 (0.95-2.84)	0.078	3.14 (1.22-8.08)	0.02	1.89 (0.64-5.56)	0.25	1.70 (0.63-4.58)	0.29
rs738408	T	C	1.73	2.14 (1.8-2.54)	$4.8*10^{-18}$	1.75 (1.4-2.2)	$1.03*10^{-6}$	2.26 (1.61-3.17)	$2.2*10^{-6}$	0.91 (0.57-1.47)	0.71	0.79 (0.30-2.02)	0.62	0.66 (0.26-1.68)	0.38

Blue shading indicates directional concordance with the effect from the European ancestry GWAS meta-analysis. Green shading indicates P<0.05. Abbreviations: EA, effect allele; RA, reference allele; OR, odds ratio; HR, hazard ratio; BMI, body mass index; HCC, hepatocellular carcinoma. GWAS odds ratios were calculated by exponentiating the per-allele estimates (betas). High alcohol intake was defined as men/women drinking more than 21/14 units per week.

Table S8. Estimates from multivariable Mendelian randomization (MVMR) with liver enzymes and cirrhosis as exposures and HCC as outcome.

Exposure	Beta (SE)	P-value	Conditional F-statistic	Q-statistic
Alanine aminotransferase	0.39 (0.17)	0.024	9.7	0.002
Cirrhosis	1.12 (0.07)	2×10^{-32}	22	
Aspartate aminotransferase	0.02 (0.14)	0.904	9.8	0.001
Cirrhosis	1.17 (0.07)	3×10^{-39}	38	
Gamma glutamyltransferase	0.09 (0.09)	0.297	10	0.016
Cirrhosis	1.18 (0.06)	9×10^{-52}	109	

Shown are the output from three MVMR analyses, each including a liver enzyme (alanine aminotransferase (ALT), aspartate aminotransferase (AST), or gamma glutamyltransferase (GGT)) together with cirrhosis. Betas and SEs are the effects on hepatocellular carcinoma. P-values refer to Betas and (SE); <0.05 is considered significant. F-statistic indicates strength of the genetic instrument (<10 indicates risk of weak instrument bias). Q-statistic assesses pleiotropy in the combined exposure instrument (<0.05 is significant).

Supplementary figures

MR: Effect on risk of HCC per SD

Exposure	n SNPs	IVW Beta (95% CI)	IVW P-value	Weighted median beta (95% CI)	Weighted median p-value	MR-Egger beta (95% CI)	MR-Egger p-value	Egger intercept p-value
Alanine aminotransferase	171	1.93 (1.53 - 2.33)	5.07e-21	1.37 (0.99 - 1.76)	2.06e-12	3.47 (2.76 - 4.18)	1.35e-17	1.35e-06
Aspartate aminotransferase	202	0.97 (0.61 - 1.33)	1.49e-07	0.40 (0.05 - 0.74)	0.024	2.05 (1.38 - 2.71)	7.24e-09	2.28e-04
Gamma glutamyltransferase	243	0.35 (0.19 - 0.51)	2.43e-05	0.29 (0.00 - 0.58)	0.049	0.16 (-0.10 - 0.42)	0.23	0.074
Haemoglobin concentration	239	0.57 (0.20 - 0.93)	2.28e-03	0.35 (-0.02 - 0.72)	0.063	0.76 (0.06 - 1.46)	0.034	0.52
Albumin	183	0.34 (0.09 - 0.59)	7.26e-03	0.07 (-0.27 - 0.41)	0.69	0.22 (-0.25 - 0.69)	0.36	0.54
Mean corpuscular volume	311	0.15 (0.03 - 0.28)	0.012	0.02 (-0.17 - 0.20)	0.87	0.20 (0.00 - 0.40)	0.049	0.56
Haematocrit percentage	222	0.43 (0.04 - 0.82)	0.032	0.15 (-0.21 - 0.52)	0.40	0.51 (-0.26 - 1.28)	0.20	0.80
Reticulocyte count	237	0.15 (-0.00 - 0.30)	0.056	0.04 (-0.20 - 0.28)	0.73	0.30 (0.04 - 0.57)	0.024	0.16
Cholesterol	147	-0.24 (-0.49 - 0.01)	0.063	-0.11 (-0.38 - 0.15)	0.40	-0.41 (-0.78 - -0.05)	0.027	0.19
C-reactive protein	173	0.16 (-0.03 - 0.36)	0.093	0.07 (-0.22 - 0.36)	0.64	0.20 (-0.07 - 0.47)	0.15	0.71
Apolipoprotein B	138	-0.16 (-0.36 - 0.04)	0.12	-0.06 (-0.22 - 0.09)	0.42	-0.26 (-0.49 - -0.02)	0.038	0.16
Calcium	172	0.19 (-0.07 - 0.45)	0.15	0.15 (-0.20 - 0.51)	0.40	0.31 (-0.15 - 0.78)	0.19	0.54
Triglycerides	198	-0.17 (-0.40 - 0.06)	0.16	-0.12 (-0.36 - 0.11)	0.31	-0.57 (-0.89 - -0.25)	6.91e-04	7.98e-04
LDL direct	141	-0.17 (-0.40 - 0.07)	0.16	-0.08 (-0.29 - 0.14)	0.49	-0.36 (-0.66 - -0.05)	0.024	0.062
Platelet count	362	-0.10 (-0.23 - 0.04)	0.17	-0.09 (-0.28 - 0.10)	0.37	-0.17 (-0.42 - 0.08)	0.18	0.48
Creatinine	274	-0.17 (-0.43 - 0.08)	0.18	-0.30 (-0.60 - -0.00)	0.048	-0.35 (-0.94 - 0.24)	0.25	0.52
Mean corpuscular haemoglobin concentration	84	0.22 (-0.12 - 0.57)	0.21	-0.04 (-0.44 - 0.36)	0.84	0.90 (0.26 - 1.55)	7.35e-03	0.017
Neutrophill count	250	0.11 (-0.08 - 0.29)	0.26	0.07 (-0.22 - 0.35)	0.64	0.14 (-0.25 - 0.53)	0.48	0.85
Testosterone	82	0.61 (-0.49 - 1.71)	0.28	0.38 (-0.33 - 1.10)	0.29	0.42 (-1.43 - 2.26)	0.66	0.80
IGF-1	295	-0.08 (-0.25 - 0.08)	0.33	-0.06 (-0.31 - 0.18)	0.61	-0.31 (-0.62 - 0.01)	0.056	0.100
SHBG	218	0.15 (-0.16 - 0.45)	0.34	0.14 (-0.15 - 0.43)	0.35	0.47 (-0.03 - 0.98)	0.067	0.11
Urea	134	-0.13 (-0.43 - 0.16)	0.38	-0.10 (-0.48 - 0.28)	0.60	-0.22 (-0.88 - 0.44)	0.52	0.78
Lymphocyte count	280	0.07 (-0.09 - 0.23)	0.39	0.00 (-0.26 - 0.26)	1.0	-0.02 (-0.39 - 0.35)	0.92	0.60
Urate	213	0.09 (-0.16 - 0.34)	0.49	0.17 (-0.03 - 0.36)	0.099	0.07 (-0.27 - 0.41)	0.68	0.87
Glycated haemoglobin	265	-0.05 (-0.19 - 0.09)	0.51	-0.19 (-0.44 - 0.07)	0.15	-0.35 (-0.57 - -0.12)	2.89e-03	1.06e-03
Direct bilirubin	66	0.07 (-0.17 - 0.30)	0.58	-0.01 (-0.11 - 0.08)	0.76	-0.03 (-0.28 - 0.22)	0.82	0.085
Cystatin C	261	-0.05 (-0.22 - 0.13)	0.60	0.08 (-0.10 - 0.27)	0.38	0.01 (-0.23 - 0.25)	0.91	0.47
Total protein	205	-0.04 (-0.26 - 0.17)	0.70	-0.25 (-0.55 - 0.05)	0.11	0.15 (-0.28 - 0.58)	0.49	0.31
Total bilirubin	120	0.03 (-0.15 - 0.21)	0.72	-0.01 (-0.09 - 0.07)	0.77	-0.03 (-0.22 - 0.16)	0.73	0.074
Glucose	93	0.04 (-0.21 - 0.29)	0.76	0.18 (-0.20 - 0.55)	0.36	0.06 (-0.36 - 0.48)	0.78	0.90
Apolipoprotein A	209	-0.03 (-0.25 - 0.19)	0.78	0.15 (-0.09 - 0.39)	0.23	0.33 (-0.00 - 0.66)	0.052	4.70e-03
Alkaline phosphatase	269	0.02 (-0.14 - 0.18)	0.81	-0.11 (-0.34 - 0.12)	0.35	-0.04 (-0.27 - 0.20)	0.77	0.53
Lipoprotein A	21	0.00 (-0.07 - 0.08)	0.91	-0.00 (-0.08 - 0.07)	0.92	0.05 (-0.03 - 0.14)	0.23	0.069
Red blood cell (erythrocyte) count	297	0.01 (-0.19 - 0.20)	0.93	0.03 (-0.28 - 0.34)	0.85	-0.07 (-0.43 - 0.29)	0.71	0.62
Phosphate	124	-0.01 (-0.23 - 0.22)	0.95	-0.07 (-0.42 - 0.27)	0.68	-0.01 (-0.39 - 0.37)	0.94	0.97
HDL cholesterol	231	-0.00 (-0.16 - 0.15)	0.97	0.14 (-0.08 - 0.36)	0.20	0.15 (-0.08 - 0.38)	0.20	0.070
Monocyte count	278	0.00 (-0.13 - 0.13)	0.99	0.04 (-0.19 - 0.26)	0.74	-0.09 (-0.30 - 0.12)	0.40	0.28

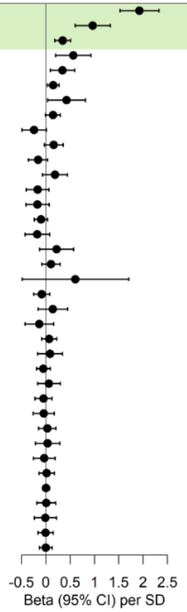

Fig. S1. Mendelian randomization analyses of the causal effect of 37 biomarkers on hepatocellular carcinoma. Genetic instruments for the biomarkers were derived from UK Biobank (UKB). Associations with hepatocellular carcinoma (HCC) were from the present study, with UKB excluded. Effect estimates are from the inverse variance weighted (IVW) method. The green bar indicates biomarkers that passed the Bonferroni-adjusted threshold for statistical significance ($P < 1.3 \times 10^{-3}$). SHBG: sex hormone binding globulin.

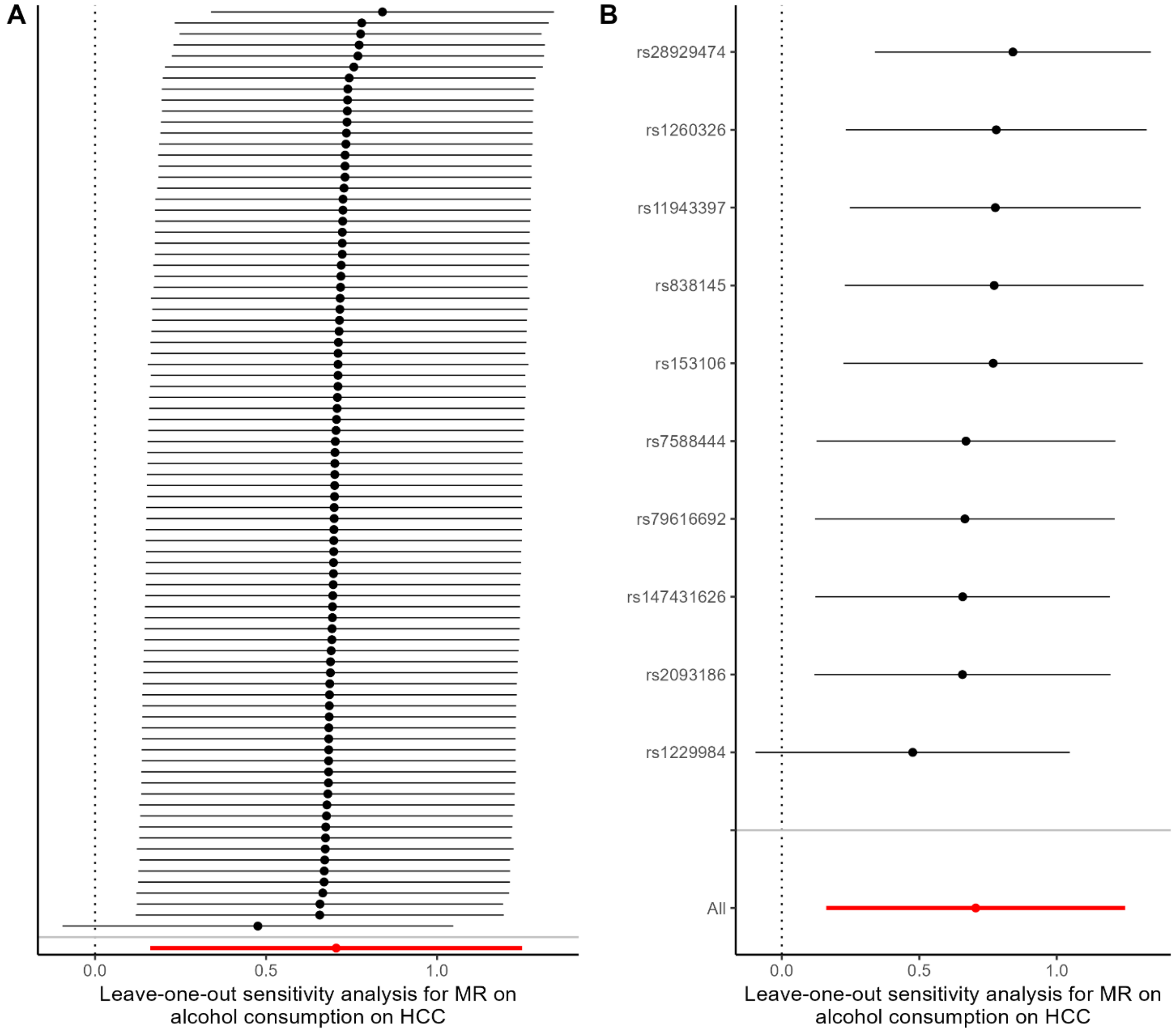

Fig. S2. Leave-one-out Mendelian randomization sensitivity analyses of alcohol consumption on hepatocellular carcinoma. The estimates are the causal estimates after leaving out one genetic variant at a time. A, shown is the leave-one-out estimates for each genetic variant included in the alcohol instrument. B, shown are the ten variants for which leaving them out of the inverse variance weighted has the largest impact.

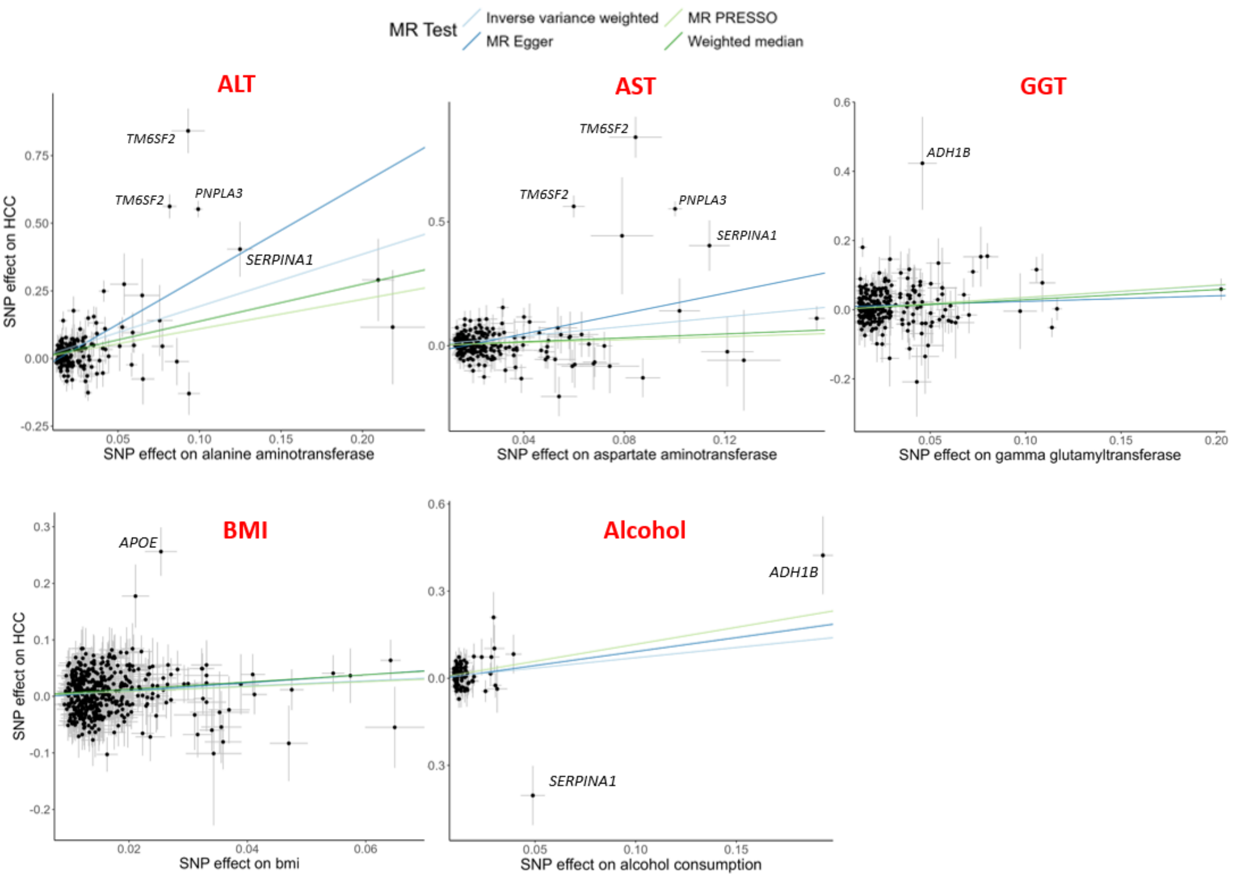

Fig. S3. Effect plots of variants included in Mendelian randomization analyses of ALT, AST, GGT, BMI, and alcohol consumption on HCC. The estimates are the estimates for the individual variants and whiskers are 95% Cis.

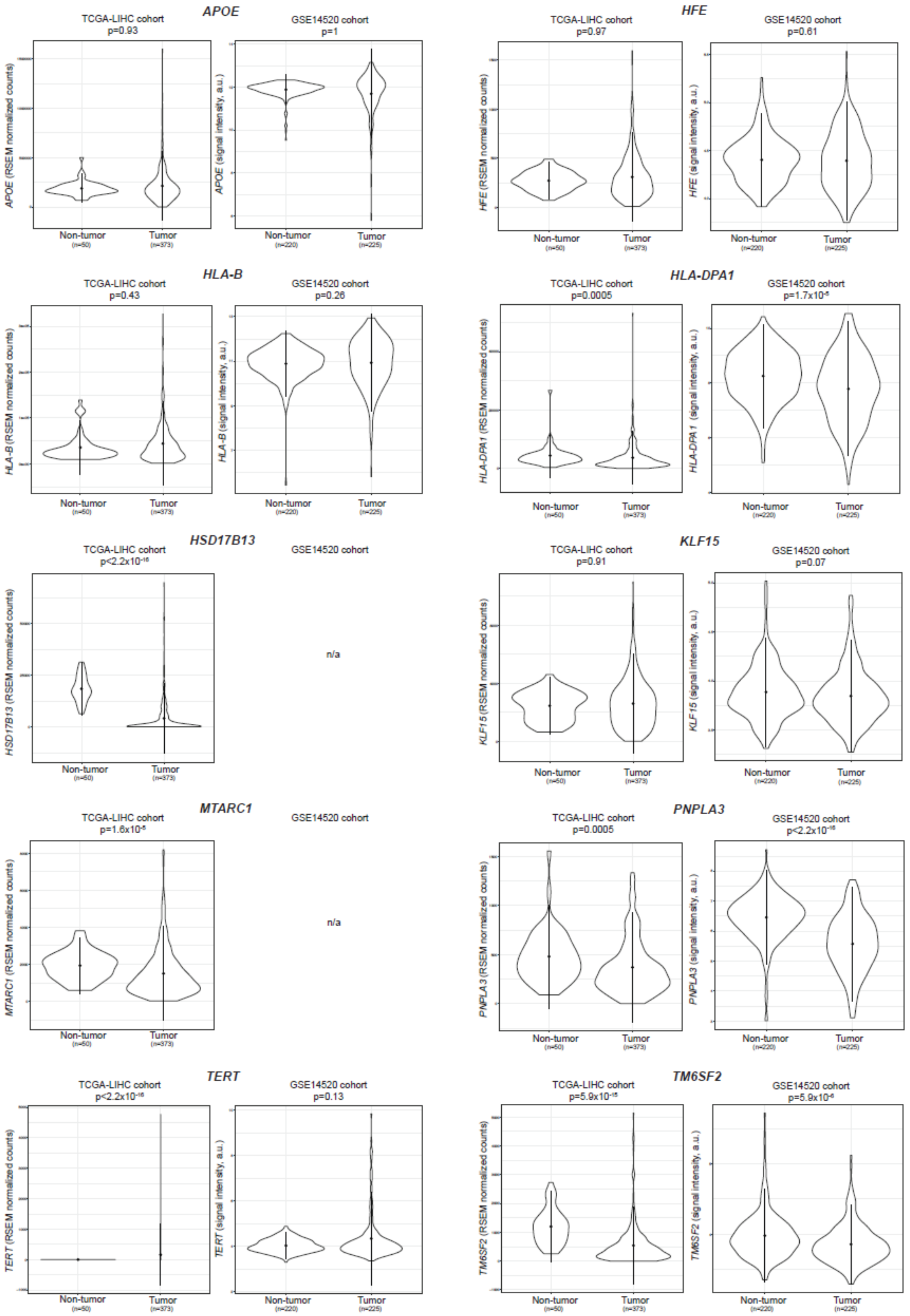

Fig. S4. Differential transcription of the ten hepatocellular carcinoma-associated genes in hepatocellular carcinomas compared to normal liver tissue.

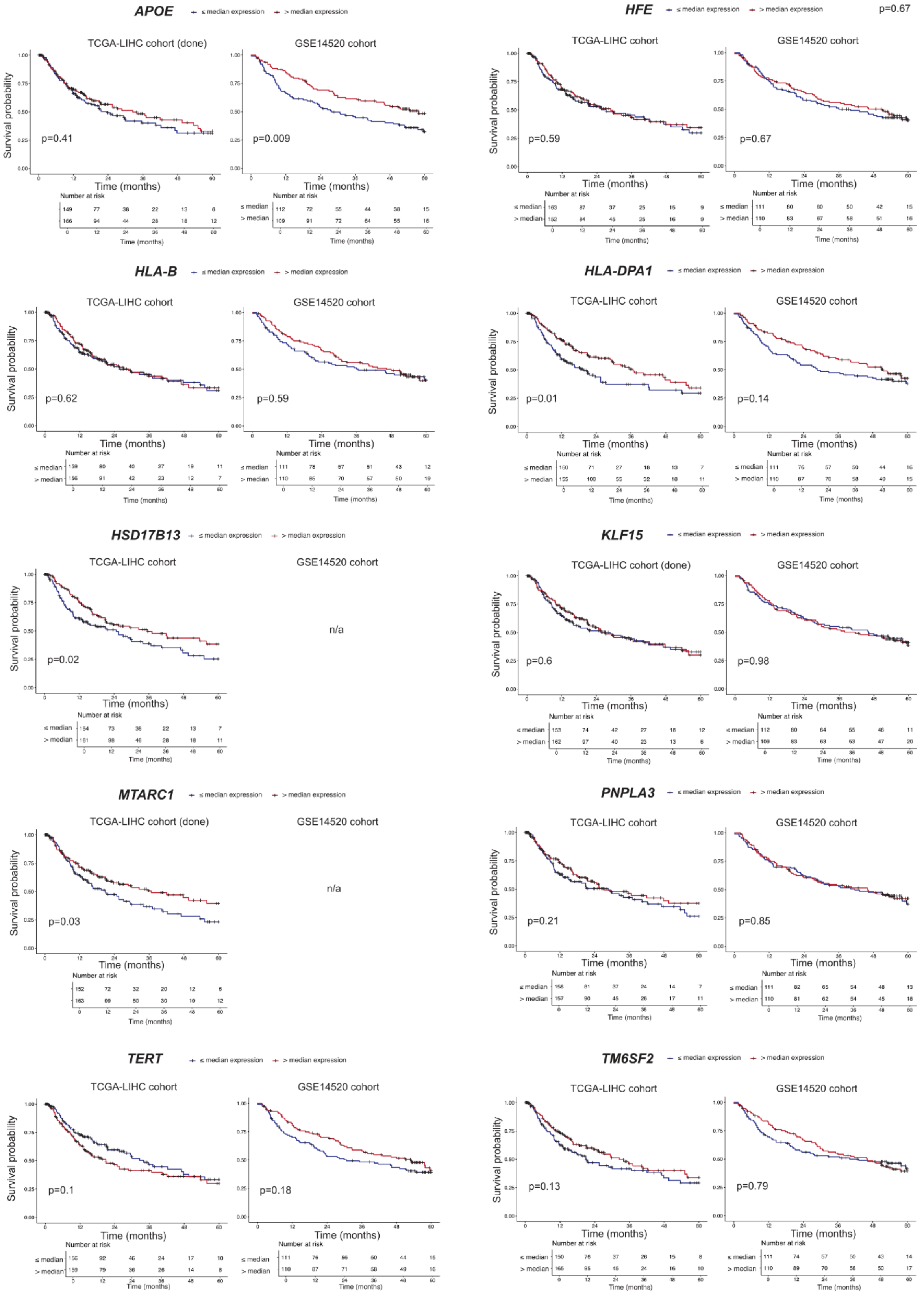

Fig. S5. Disease-free survival in hepatocellular carcinoma as a function of transcriptional level of the ten hepatocellular carcinoma-associated genes. The curves depict disease-free survival in HCC cases, stratified by RNA levels above (red) or below (blue) the median level.

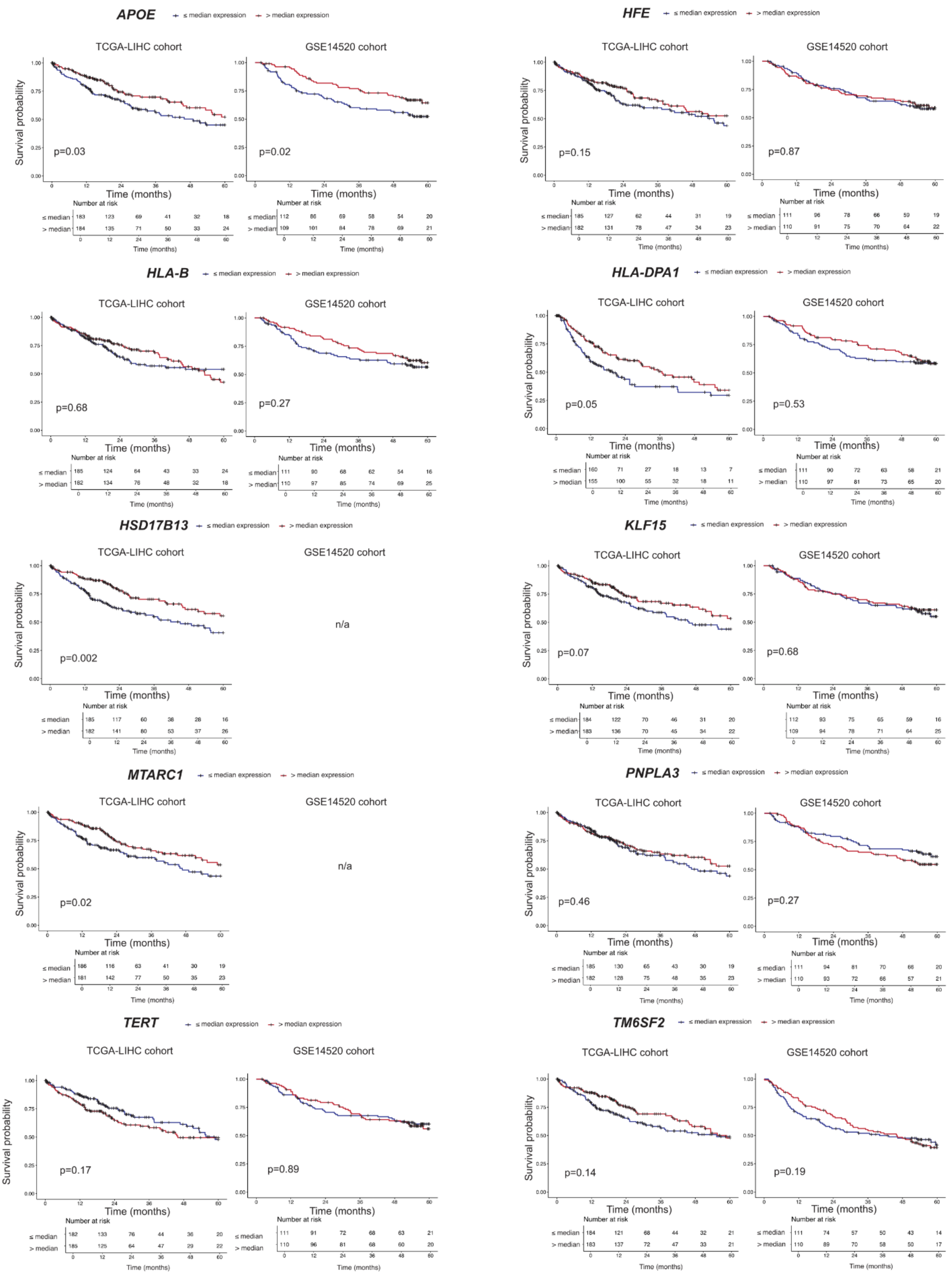

Fig. S6. Overall survival in hepatocellular carcinoma as a function of transcriptional level of the ten hepatocellular carcinoma-associated genes. The curves depict overall survival in HCC cases, stratified by RNA levels above (red) or below (blue) the median level.

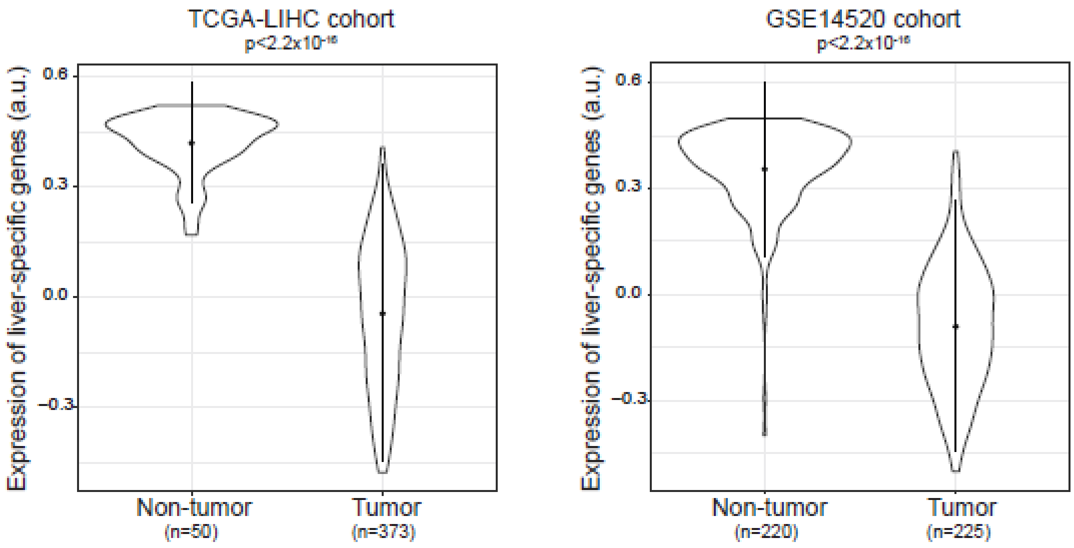

Fig. S7. Transcriptional level of 541 liver-specific genes in non-tumor and HCC. The panel of liver-specific genes was defined in Farshidfar et al[19].

Supplementary references

1. Sørensen E, Christiansen L, Wilkowski B, et al. Data Resource Profile: The Copenhagen Hospital Biobank (CHB). Int J Epidemiol 2021;50.
2. Hansen TF, Banasik K, Erikstrup C, et al. DBDS Genomic Cohort, a prospective and comprehensive resource for integrative and temporal analysis of genetic, environmental and lifestyle factors affecting health of blood donors. BMJ Open 2019;9.
3. Sveinbjornsson G, Ulfarsson MO, Thorolfsdottir RB, et al. Multiomics study of nonalcoholic fatty liver disease. Nat Genet 2022;54.
4. Sigurdardottir LG, Jonasson JG, Stefansdottir S, et al. Data quality at the Icelandic Cancer Registry: Comparability, validity, timeliness and completeness. Acta Oncol (Madr) 2012;51.
5. Kong A, Masson G, Frigge ML, et al. Detection of sharing by descent, long-range phasing and haplotype imputation. Nat Genet 2008;40.
6. Gudbjartsson DF, Helgason H, Gudjonsson SA, et al. Large-scale whole-genome sequencing of the Icelandic population. Nat Genet 2015;47.
7. Sudlow C, Gallacher J, Allen N, et al. UK Biobank: An Open Access Resource for Identifying the Causes of a Wide Range of Complex Diseases of Middle and Old Age. PLoS Med 2015;12.
8. Leitsalu L, Haller T, Esko T, et al. Cohort profile: Estonian biobank of the Estonian genome center, university of Tartu. Int J Epidemiol 2015;44.
9. Anon. Genomic data in the All of Us Research Program. Nature 2024;627.
10. Trépo E, Caruso S, Yang J, et al. Common genetic variation in alcohol-related hepatocellular carcinoma: a case-control genome-wide association study. Lancet Oncol 2022;23.
11. Machiela MJ, Huang WY, Wong W, et al. GWAS Explorer: an open-source tool to explore, visualize, and access GWAS summary statistics in the PLCO Atlas. Sci Data 2023;10.
12. Nagai A, Hirata M, Kamatani Y, et al. Overview of the BioBank Japan Project: Study design and profile. J Epidemiol 2017;27.
13. Sakaue S, Kanai M, Tanigawa Y, et al. A cross-population atlas of genetic associations for 220 human phenotypes. Nat Genet 2021;53.
14. Walters RG, Millwood IY, Lin K, et al. Genotyping and population characteristics of the China Kadoorie Biobank. Cell Genomics 2023;3.
15. Kurki MI, Karjalainen J, Palta P, et al. FinnGen provides genetic insights from a well-phenotyped isolated population. Nature 2023;613.
16. Song C, Lv J, Yu C, et al. Adherence to Healthy Lifestyle and Liver cancer in Chinese: a prospective cohort study of 0.5 million people. Br J Cancer 2022;126:815–821.
17. Ukawa S, Okada E, Nakamura K, et al. Characteristics of patients with liver cancer in the BioBank Japan project. J Epidemiol 2017;27:S43–S48.

18. MacParland SA, Liu JC, Ma X-Z, et al. Single cell RNA sequencing of human liver reveals distinct intrahepatic macrophage populations. Nat Commun 2018;9:4383.

19. Farshidfar F, Zheng S, Gingras M-C, et al. Integrative Genomic Analysis of Cholangiocarcinoma Identifies Distinct IDH-Mutant Molecular Profiles. Cell Rep 2017;18:2780–2794.

Consortia members

Danish Blood Donor Study (DBDS) genomic consortium

n	Academic degrees	First name	Surname	Email	Affiliation 1	Affiliation 2	Banner
1	PhD	Karina	Banasik	karina.banasik@cpr.ku.dk	Novo Nordisk Foundation Center for Protein Research, Faculty of Health and Medical Sciences, University of Copenhagen, Copenhagen, Denmark		DBDS GC (Banner)
2	PhD	Jakob	Bay	jabay@regionsjaelland.dk	Department of Clinical Immunology, Zealand University Hospital, Køge, Denmark		DBDS GC (Banner)
3	MSc	Jens Kjærgaard	Boldsen	jenbol@rm.dk	Department of Clinical Immunology, Aarhus University Hospital, Aarhus, Denmark		DBDS GC (Banner)
4	PhD	Thorsten	Brodersen	thobr@regionsjaelland.dk	Department of Clinical Immunology, Zealand University Hospital, Køge, Denmark		DBDS GC (Banner)
5	PhD	Søren	Brunak	soren.brunak@cpr.ku.dk	Novo Nordisk Foundation Center for Protein Research, Faculty of Health and Medical Sciences, University of Copenhagen, Copenhagen, Denmark		DBDS GC (Banner)
6	PhD	Alfonso	Buil Demur	alfonso.buil.demur@regionh.dk	Institute of Biological Psychiatry, Mental Health Centre, Sct. Hans, Copenhagen University Hospital, Roskilde, Denmark		DBDS GC (Banner)
7	**PhD**	Lea Arregui Nordahl	Christoffersen	lea.arregui.nordahl.christoffersen@regionh.dk	Department of Clinical Immunology, Zealand University Hospital, Køge, Denmark		
8	PhD	Maria	Didriksen	maria.didriksen@regionh.dk	Department of Clinical Immunology, Copenhagen University Hospital, Rigshospitalet, Copenhagen, Denmark		DBDS GC (Banner)
9	PhD	Khoa Manh	Dinh	khoadinh@rm.dk	Department of Clinical Immunology, Aarhus University Hospital, Aarhus, Denmark		DBDS GC (Banner)
10	PhD	Joseph	Dowsett	joseph.dowsett@regionh.dk	Department of Clinical Immunology, Copenhagen University Hospital, Rigshospitalet, Copenhagen, Denmark		DBDS GC (Banner)
11	PhD	Christian	Erikstrup	christian.erikstrup@skejby.rm.dk	Department of Clinical Immunology, Aarhus University Hospital, Aarhus, Denmark	Department of Clinical Medicine, Health, Aarhus University, Aarhus, Denmark	DBDS GC (Banner)
12	PhD	Bjarke	Feenstra	Bjarke Feenstra <FEE@ssi.dk>	Department of Clinical Immunology, Copenhagen University Hospital, Rigshospitalet, Copenhagen, Denmark	Department of Epidemiology Research, Statens Serum Institut, Copenhagen, Denmark	DBDS GC (Banner)
13	PhD	Frank	Geller	FGE@ssi.dk	Department of Clinical Immunology, Copenhagen University Hospital, Rigshospitalet, Copenhagen, Denmark	Department of Epidemiology Research, Statens Serum Institut, Copenhagen, Denmark	DBDS GC (Banner)
14	PhD	Daniel	Gudbjartsson	Daniel.Gudbjartsson@decode.is	deCODE Genetics, Reykjavik, Iceland		DBDS GC (Banner)
15	PhD	Thomas Folkmann	Hansen	thomas.hansen@regionh.dk	Danish Headache Center, Department of Neurology, Copenhagen University Hospital, Rigshospitalet-Glostrup, Copenhagen, Denmark		DBDS GC (Banner)
16	PhD	Dorte	Helenius Mikkelsen	dorte.helenius.mikkelsen@regionh.dk	Institute of Biological Psychiatry, Mental Health Centre, Sct. Hans, Copenhagen University Hospital, Roskilde, Denmark		DBDS GC (Banner)

17	MSc	Lotte	Hindhede	LOTHIN@rm.dk	Department of Clinical Immunology, Aarhus University Hospital, Aarhus, Denmark		DBDS GC (Banner)
18	PhD	Henrik	Hjalgrim	HHJ@cancer.dk	Danish Cancer Society Research Center, Copenhagen, Denmark	Department of Epidemiology Research, Statens Serum Institut, Copenhagen, Denmark	DBDS GC (Banner)
19	PhD	Jakob	Hjorth von Stemann	jakob.hjorth.von.stemann@regionh.dk	Department of Clinical Immunology, Copenhagen University Hospital, Rigshospitalet, Copenhagen, Denmark		DBDS GC (Banner)
20	MD	Bitten Aagaard	Jensen	biaaj@rn.dk	Department of Clinical Immunology, Aalborg University Hospital, Aalborg, Denmark		DBDS GC (Banner)
21	PhD	Andrew	Joseph Schork	andrew.joseph.schork@regionh.dk	Institute of Biological Psychiatry, Mental Health Centre, Sct. Hans, Copenhagen University Hospital, Roskilde, Denmark		DBDS GC (Banner)
22	PhD	Kathrine	Kaspersen	kathkasp@rm.dk	Department of Clinical Immunology, Aarhus University Hospital, Aarhus, Denmark		DBDS GC (Banner)
23	MSc	Bertram Dalskov	Kjerulff	berkje@rm.dk	Department of Clinical Immunology, Aarhus University Hospital, Aarhus, Denmark		DBDS GC (Banner)
24	PhD	Mette	Kongstad	mette.kongstad.01@regionh.dk	Department of Clinical Immunology, Copenhagen University Hospital, Rigshospitalet, Copenhagen, Denmark		DBDS GC (Banner)
25	PhD	Susan	Mikkelsen	susanmke@rm.dk	Department of Clinical Immunology, Aarhus University Hospital, Aarhus, Denmark		DBDS GC (Banner)
26	MD	Christina	Mikkelsen	christina.mikkelsen@regionh.dk	Department of Clinical Immunology, Copenhagen University Hospital, Rigshospitalet, Copenhagen, Denmark		DBDS GC (Banner)
27	PhD	Ioanna	Nissen	ioanna.nissen@regionh.dk	Department of Clinical Immunology, Copenhagen University Hospital, Rigshospitalet, Copenhagen, Denmark		DBDS GC (Banner)
28	PhD	Mette	Nyegaard	nyegaard@hst.aau.dk	Department of Health Science and Technology, Faculty of Medicine, Aalborg Univeristy, Aalborg, Denmark		DBDS GC (Banner)
29	DMSc	Sisse Rye	Ostrowski	sisse.rye.ostrowski@regionh.dk	Department of Clinical Immunology, Copenhagen University Hospital, Rigshospitalet, Copenhagen, Denmark	Department of Clinical Medicine, Faculty of Health and Medical Sciences, University of Copenhagen, Copenhagen, Denmark	DBDS GC (Banner)
30	PhD	Ole Birger	Pedersen	olbp@regionsjaelland.dk	Department of Clinical Immunology, Zealand University Hospital, Køge, Denmark	Department of Clinical Medicine, Faculty of Health and Medical Sciences, University of Copenhagen, Copenhagen, Denmark	DBDS GC (Banner)
31	PhD	Liam James Elgaard	Quinn	liaq@regionsjaelland.dk	Department of Clinical Immunology, Zealand University Hospital, Køge, Denmark		DBDS GC (Banner)
32	PhD	Þórunn	Rafnar	Thorunn.Rafnar@decode.is	deCODE Genetics, Reykjavik, Iceland		DBDS GC (Banner)
33	PhD	Palle Duun	Rohde	palledr@hst.aau.dk	Department of Health Science and Technology, Faculty of Medicine, Aalborg Univeristy, Aalborg, Denmark		DBDS GC (Banner)

34	PhD	Klaus	Rostgaard	klar@cancer.dk	Danish Cancer Society Research Center, Copenhagen, Denmark	Department of Epidemiology Research, Statens Serum Institut, Copenhagen, Denmark	DBDS GC (Banner)
35	PhD	Michael	Schwinn	michael.schwinn@regionh.dk	Department of Clinical Immunology, Copenhagen University Hospital, Rigshospitalet, Copenhagen, Denmark		DBDS GC (Banner)
36	PhD	Erik	Sørensen	Erik.Soerensen@regionh.dk	Department of Clinical Immunology, Copenhagen University Hospital, Rigshospitalet, Copenhagen, Denmark		DBDS GC (Banner)
37	PhD	Kari	Stefansson	kari.stefansson@decode.is	deCODE Genetics, Reykjavik, Iceland		DBDS GC (Banner)
38	PhD	Hreinn	Stefánsson	hreinn.stefansson@decode.is	deCODE Genetics, Reykjavik, Iceland		DBDS GC (Banner)
39	PhD	Lise Wegner	Thørner	Lise.Wegner.Thoerner@regionh.dk	Department of Clinical Immunology, Copenhagen University Hospital, Rigshospitalet, Copenhagen, Denmark		DBDS GC (Banner)
40	PhD	Unnur	Þorsteinsdóttir	Unnur.Thorsteinsdottir@decode.is	deCODE Genetics, Reykjavik, Iceland		DBDS GC (Banner)
41	MD	Mie	Topholm Bruun	mie.topholm.bruun@rsyd.dk	Department of Clinical Immunology, Odense University Hospital, Odense, Denmark		DBDS GC (Banner)
42	PhD	Henrik	Ullum	HEUL@ssi.dk	Statens Serum Institut, Copenhagen, Denmark		DBDS GC (Banner)
43	PhD	Thomas	Werge	thomas.werge@regionh.dk	Institute of Biological Psychiatry, Mental Health Centre, Sct. Hans, Copenhagen University Hospital, Roskilde, Denmark	Department of Clinical Medicine, Faculty of Health and Medical Sciences, University of Copenhagen, Copenhagen, Denmark	DBDS GC (Banner)
44	PhD	David	Westergaard	david.westergaard@cpr.ku.dk	Novo Nordisk Foundation Center for Protein Research, Faculty of Health and Medical Sciences, University of Copenhagen, Copenhagen, Denmark		DBDS GC (Banner)

Estonian Biobank

Name	Affiliation(s)
Lili Milani	Estonian Genome Centre, Institute of Genomics, University of Tartu, Tartu, Estonia
Andres Metspalu	Estonian Genome Centre, Institute of Genomics, University of Tartu, Tartu, Estonia
Tõnu Esko	Estonian Genome Centre, Institute of Genomics, University of Tartu, Tartu, Estonia
Mari Nelis	Estonian Genome Centre, Institute of Genomics, University of Tartu, Tartu, Estonia
Georgi Hudjashov	Estonian Genome Centre, Institute of Genomics, University of Tartu, Tartu, Estonia
